# ﻿A review of *Eupholidoptera* (Orthoptera, Tettigoniidae) from Crete, Gavdos, Gavdopoula, and Andikithira

**DOI:** 10.3897/zookeys.1151.97514

**Published:** 2023-03-01

**Authors:** Luc Willemse, Jos Tilmans, Nefeli Kotitsa, Apostolos Trichas, Klaus-Gerhard Heller, Dragan Chobanov, Baudewijn Odé

**Affiliations:** 1 Naturalis, PO Box 9517, 2300, RA Leiden, Netherlands Naturalis Leiden Netherlands; 2 Herkenboscher Strasse 33, 41849, Wassenberg-Rothenbach, Germany Unaffiliated Wassenberg-Rothenbach Germany; 3 Natural History Museum Crete, University of Crete, P.O.Box 2208, 71409, Heraklion, Crete, Greece University of Crete Crete Greece; 4 Institute of Biodiversity and Ecosystem Research, Bulgarian Academy of Sciences, 1 Tsar Osvoboditel blvd., 1000, Sofia, Bulgaria Institute of Biodiversity and Ecosystem Research, Bulgarian Academy of Sciences Sofia Bulgaria; 5 Triesdorf Bahnhof 8, 91732, Merkendorf, Germany Unaffiliated Merkendorf Germany; 6 Riethorsterweg 12, 6586, AC Plasmolen, Netherlands Unaffiliated Plasmolen Netherlands

**Keywords:** Bioacoustics, faunistics, Greece, new species, phylogeography, systematics, traps

## Abstract

Being nocturnal, hiding in prickly bushes and shrubs during the day, *Eupholidoptera* species in Crete and its neighbouring islands are easily overlooked, and until now our knowledge about their distribution was based on some thirty sightings across 11 species. In this paper results are presented of a study of *Eupholidoptera* specimens collected between 1987 and 2020 by hand-catches and pitfall and fermenting traps on the Greek islands of Crete, Gavdos, Gavdopoula, and Andikithira. Diagnostic features of all known species are presented and illustrated with stacked images. An updated key to all species is provided. *Eupholidopterafrancisae* Tilmans & Odé, **sp. nov.** from Andikithira and southwestern Crete and *Eupholidopteramarietheresae* Willemse & Kotitsa, **sp. nov.** from Mt. Dikti are described. Female *E.cretica*, *E.gemellata*, and *E.mariannae* are described, and the female of *E.astyla* is redescribed. Bioacoustics for *E.francisae* Tilmans & Odé, **sp. nov**., *E.giuliae*, and *E.jacquelinae* are presented for the first time. *Eupholidopterasmyrnensis* is reported for the first time from Crete. A substantial amount of new distribution data for *Eupholidoptera* species on Crete is presented. The current distribution pattern and first analyses of phylogeny based on molecular data of *Eupholidoptera* species on Crete are discussed in relation to paleogeographical events.

## ﻿Introduction

**Figure 1.**
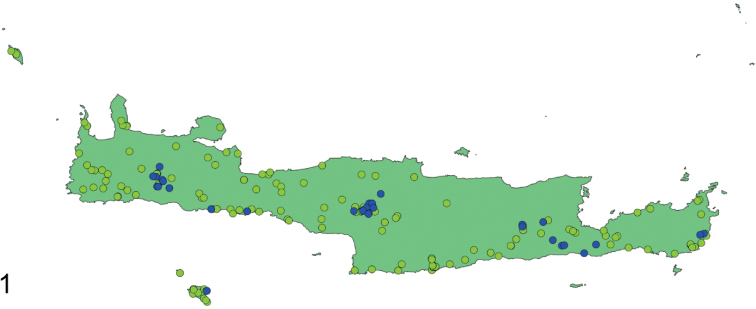
Occurrence records *Eupholidoptera* from Crete, Gavdos, Gavdopoula, and Andikithira. Blue dots: published locations; green dots: new locations.

*Eupholidoptera* is a Mediterranean bush-cricket genus with 54 species belonging to the tribe Pholidopterini (Çiplak et al. 2021). It is distributed from southern France to Turkey and the Middle East (Çiplak et al. 2009). Most species are found in Greece and Turkey, each country listing more than 20 species. With an amazing eleven species, Crete, with its satellite islands, forms a hotspot for *Eupholidoptera* in Greece. Besides mesophytic vegetation, *Eupholidoptera* species are often associated with maquis and phrygana vegetation. This also applies to Crete where *Eupholidoptera* species live in thorny, spiny shrubs and bushes. These can be tall shrubs like *Rubus* and *Calicotome* on which they can be found sunbathing in the early morning, as well as low shrubs like *Sarcopoterium*. *Eupholidoptera* are nocturnal, hiding during the day inside shrubs and bushes. Males start singing late in the afternoon. The spiny and dense vegetation and the overall nocturnal activity pattern make it quite challenging to actually see *Eupholidoptera* during the day, let alone to collect them. In addition, *Eupholidoptera* can only be found during certain parts of the summer season. In lowlands, adult *Eupholidoptera* appear in May or early June and disappear again late July or August whereas at higher altitudes adults appear from late June onwards and may be found up to October. Table 1 presents the total number of all locations published up to now for *Eupholidoptera* species from Crete and its satellite islands. Because of the limited number of known locations, little was known in detail about distribution patterns of *Eupholidoptera* species in Crete. Previously known locations presented in Table 1 and Fig. 1 (blue dots) suggest that *Eupholidoptera* species are restricted to specific altitudinal ranges and absent from large parts of Crete, especially some lowland areas. Based on published locations and known threats, all Cretan species of *Eupholidoptera* were assessed in one of the IUCN threatened categories in 2016 (Hochkirch et al. 2016). In order to gain a better understanding of distribution patterns of *Eupholidoptera* as well as their phenology and habitat preferences, Crete and the islands of Andikithira (32 km northwest of Crete) and Gavdos (36.5 km south of southwestern Crete) were visited by the Dutch and German authors on various occasions between 1987 and 2020. In the same period and parallel to the efforts to collect *Eupholidoptera* using hand catches, *Eupholidoptera* species have also been trapped extensively as by-catch in pitfall and fermenting traps, across Crete and its satellite islands. This paper summarises the systematic and faunistic results based on specimens collected between 1987 and 2020. An updated key is presented. For each species, diagnostic features are given illustrated with stacked images, supplemented with a differential diagnosis and updated information on distribution, habitat, and phenology. In addition, comments are made on morphological variation found in the specimens studied.

**Table 1.** Published record of *Eupholidoptera* species from Crete and Gavdos.

Species	Number of locations	Altitude (m)	Reference
*E.annamariae*	2	50–200	Nadig 1985; Çiplak et al. 2009
*E.astyla*	10	800–1850	Ramme 1927; Çiplak et al. 2009
*E.cretica*	1	not indicated	Ramme 1951
*E.feri*	1	1100	Willemse and Heller 2001
*E.forcipata*	3	1700–1850	Willemse and Kruseman 1976; Çiplak et al. 2009
*E.gemellata*	1	1650	Willemse and Kruseman 1976
*E.giuliae*	3	0–175	Massa 1999; Çiplak et al. 2009
*E.jacquelinae*	1	50	Tilmans 2002; Çiplak et al. 2009
*E.latens*	7	500–1800	Willemse and Kruseman 1976; Çiplak et al. 2009
*E.mariannae*	4^(1)^	500–700	Willemse and Heller 2001; Ramme 1927
*E.pallipes*	1	1600–1800	Willemse and Kruseman 1976

^1^: includes two locations of paratypes described by Ramme (1927) under *E.astyla*.

## ﻿Materials and methods

### ﻿Hand catches

Hand catches included scanning larger shrubs (*Rubus*, *Calicotome*) in the morning to check for sunbathing individuals, walking through a terrain, and checking for individuals hiding in spiny bushes during the day. Additionally, singing males can be located using audio equipment late in the afternoon and in combination with a (head) light in the first half of the night. Specimens were killed using ethylacetate or potassium cyanide, eviscerated, the belly filled with cotton wool, and airdried.

### ﻿Rearing

Specimens found as nymphs were reared to adults. For this, cylindrical plastic containers with small twigs were used and nymphs were fed with oat flakes. In some cases, however, very young nymphs that, based on their dark colour pattern, were assumed to be *Eupholidoptera* turned out to belong to different genera (for instance *Incertana* Zeuner, 1941).

### ﻿Traps

The traps were part of MSc and PhD studies aimed at the epigeal fauna of Crete and environmental monitoring programs, carried out by the Natural History Museum of the University of Crete (Trichas et al. 2008). The main research goals of those studies were at first the description of diversity, phenology, and biogeography of several ground-dwelling beetle families (Carabidae, Tenebrionidae, Staphylinidae, etc.), while in the middle of the 1990s, Gnaphosidae and other ground spider families were added, and in the late 2000s chilopods, diplopods, and isopods were included. Details on trap protocols (active time of trapping, years, trap dimensions, chemicals, etc.) and trap efficiency are discussed in publications linked to these and other studies (Trichas et al. 2008; Kaltsas and Simaiakis 2012; Kaltsas et al. 2013; Salata et al. 2020).

Two types of traps were used, pitfall traps dug into the soil and fermenting traps placed higher up in the vegetation.

**Figures 2, 3.**
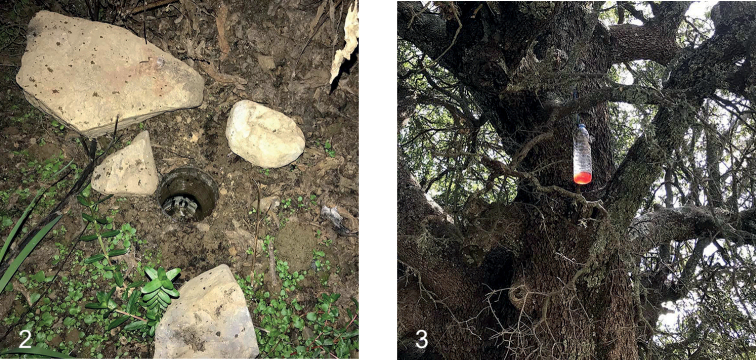
Traps **2** pitfall trap **3** fermenting trap.

Pitfall traps (Fig. 2) used between 1988 and 2019 consisted of standardised sized cups (9.5 cm diameter, 12 cm depth) placed in a standardised pattern (15 per sampling station, stations always of homogenous biotope appearance) (Trichas et al. 2008; Kaltsas et al. 2013). Ethylene glycol was used as a killing/preserving agent being replaced by undiluted propylene glycol during the last ten years. Occasionally, a small quantity of attractants like vinegar, or surface tension reducers like liquid soap, were used. Traps were placed as far away as possible from visible ant nests, as ants tend to gather in masses in the container cups, preventing many of the targeted arthropods to be successfully caught. Sampling stations were active for one-year-round (occasionally with an additional two months in the next year) and were sampled bimonthly (Kaltsas and Simaiakis 2012), six (or seven) times in total. At elevations above 1500 m on the mountains, which were covered with snow from November or December till late April of the next year, usually only three samples per year were collected. For instance, traps set in April were emptied and refilled with propylene glycol in June, emptied and refilled again in August, etc., all year round. Locations with pitfall stations were usually remote from each other, as the biogeography of Crete was always one of the experimental targets. The numbers of *Eupholidoptera* specimens recovered from the contents of a single trapping event were usually fewer than ten, but quite regularly traps contained several dozens of specimens. The highest numbers recorded in a single trapping event were 189 specimens of *E.astyla* and 513 specimens of *E.annamariae*.

Fermenting traps (Fig. 3) were only used between March 2018 and August 2019 primarily aimed at saproxylic beetles. Not all sampling stations were active for the entire duration of the study. In total, 24 stations around Crete were sampled (Bolanakis 2019; Bolanakis and Trichas 2019). The fermenting traps consisted of plastic water bottles (1.5 L) with an entrance hole in the top half containing a mix of alcoholic ingredients, sugars and some preservative (as in Galford 1980; Nageleisen and Bouget 2009). The mix that lured the *Eupholidoptera* specimens consisted of vinegar, sugar, salt, alcohol, yeast, glucose and rotten banana (Bolanakis 2019). Ingredients were stirred manually until the sugar and salt were dissolved and 0.2–0.3 L of the mix was poured in each bottle. Bottles were hanged inside the foliage and tied on tree or bush branches with a string. Trap stations were revisited every one or two months to replace traps and take the collected samples, after adding pure alcohol, to the laboratory (Bolanakis 2019). *Eupholidoptera* were collected in fermenting traps at nine different locations. With the exception of one trap placed in a *Pinus* forest which collected 28 specimen of *E.mariannae* in a six-week period, only few specimens (fewer than five) were found in fermenting traps.

### ﻿Storage medium

Specimens collected by hand were dried and pinned. Specimens caught by traps are stored in 70% alcohol except for a few which, after examination, were pinned. For the storage medium for each of the examined specimens see Suppl. material 2.

### ﻿DNA samples

Part of the DNA samples used for phylogenetic analysis derived from right mid legs from specimens collected in 2014, 2017, and 2019 (see Suppl. material 2). Additional DNA samples were taken from specimens collected in pitfall traps and from dried, pinned specimens. Most specimens collected by pitfall traps did not yield good quality DNA due to the age of the samples, the collection method (deceased specimens stayed in the traps for weeks without ethanol and DNA-degrading agents such as vinegar before collection), and preservation (specimens were stored in room temperature). Dried, hand-picked samples had a better success rate than pitfall and fermenting trap catches. Still, some older dry samples (mainly collected before 2004) failed to yield good quality DNA results, especially regarding mitochondrial DNA, which is more prone to degradation compared to nuclear DNA.

### ﻿DNA isolation and amplification

DNA was extracted from femoral muscles of *Eupholidoptera* specimens that were preserved either in alcohol (75% or 95%) or dry mounted. For recent and well-preserved specimens, a standard protocol for DNA isolation using ethanol precipitation was used. For dry-mounted and old, alcohol-preserved specimens from pitfall and fermenting traps DNA-isolation kits were used (Qiagen DNeasy Blood & Tissue Kits and Invitrogen purelink genomic DNA mini kit) following the manufacturers’ instructions.

Sequence data for one protein-coding mitochondrial gene (NADH dehydrogenase subunit 2 – NADH2) and one non-coding nuclear region (the internal transcribed spacers 1 and 2 together with the 5.8S rDNA gene in-between – ITS) where used. For amplification of NADH2 the primers used were TM-J210 AATTAAGCTAATGGGTTCATACCC (forward) and TW-N1284 AYAGCTTTGAARGYTATTAGTTT (reverse) (Simon et al. 2006). The ITS fragment was amplified with the primers WeekF TAGAGGAAGTAAAAGTCG (forward) and WeekR GCTTAAATTCAGCGG (reverse) (Weekers et al. 2001)”schema”:”https://github.com/citation-style-language/schema/raw/master/csl-citation.json”}.

Polymerase chain reactions were performed using Thermo Scientific DreamTaq Hot Start Master Mix according to the manufacturer’s instructions. Temperature cycling for the NADH2 followed Chobanov et al. (2017), with adaptations for hot-start PCR and slight adjustments, as follows: initial step at 95 °C for 5 min, followed by 35 cycles of denaturation (95 °C for 50 sec), annealing (51 °C for 40 sec), and elongation (72 °C for 80 sec), with a final elongation step at 72 °C for 15 min. Temperature cycling for the ITS fragment followed the protocol by Ullrich et al. (2010).

Additional sequences for both loci were obtained from GenBank (https://www.ncbi.nlm.nih.gov/genbank/).

### ﻿Phylogenetic analyses

Obtained sequences were trimmed, assembled, and visually checked using CodonCode Aligner v. 8.0.2 (CodonCode, Dedham, MA, USA). All protein-coding sequences were checked for numt possibility and unique haplotypes were selected using DAMBE 7.2.152 (Xia, 2018). Sequence alignments were performed in MEGA-X (Kumar et al. 2018) using the MUSCLE algorithm. The protein-coding sequences were checked for saturation and the two loci were concatenated using DAMBE 7.2.152. Nucleotide-substitution models for all codon positions of the NADH2 and a single partition of the ITS were estimated with Partition Finder 2.1.1 (Lanfear et al. 2016) under the corrected Akaike information criterion and implemented in subsequent phylogenetic analyses.

Bayesian inference (BI) phylogenetic analysis was performed on the concatenated dataset (NADH2 + ITS) using Mr. Bayes v. 3.2.7 (Ronquist and Huelsenbeck 2003), with four simulations of Markov chains and 4 × 10^6^ generations sampling each 100^th^ tree. Stationary distribution of the MCMC parameters was checked with Tracer ver. 1.7.1 (Rambaut et al. 2018) multivariate visualization, demographic trajectory re-construction, conditional posterior distribution summary, and more. Tracer is open-source and available at http://beast.community/tracer. The first 25% of trees were excluded as burn-in. Results were visualized in FigTree 1.4.4 (http://tree.bio.ed.ac.uk/software/figtree/).

### ﻿Geographical coordinates

Coordinates are presented in decimal degrees (DD) and may therefore differ from collecting labels that use degrees, minutes and seconds or degrees and minutes (DMS). For locations for which no coordinates were available Google Earth has been used in which case coordinates have been placed between square brackets “ []”.

### ﻿Dissection of titillators

Titillators, once removed, were cleared in a KOH solution and fixed with glue on a small board, pinned under the specimen or in case of *E.latens* from Rhodopos, transferred to glycerol (Aspöck 1971).

### ﻿Bioacoustics

For song recordings several digital recorder systems (digital tape, solid state memory and computer hard disk) and microphones were used. Usually a frequency response better than 50–20,000 Hz were achieved. Most recordings were made indoors in a lab or room, in the evening or night, using (partly) open containers, frequently housed in an anechoic cupboard, with the microphone at 3–15 cm distance. The air temperature during recording in room or studio was between 15 °C and 27 °C. We did not attempt to correct for the possible body temperature of the animals, which may well have been below or above the measured room temperature. Specific data for the individual recordings can be found in Suppl. material 3.

Song analysis of the digital recordings has been performed using Wavelab software (www.steinberg.net). Oscillograms have been prepared using Praat software (www.praat.org).

Bioacoustic terminology: calling song – the song produced by an isolated male; syllable – the sound produced by one opening-and-closing movement of the tegmina; hemisyllable – the sound produced by the opening or closing movement of the tegmina; syllable period – period from one syllable beginning to the next. Syllable repetition rate – the number of syllables produced per second.

### ﻿Photography

For stacked images, a Zeiss SteREO Discovery V20 stereomicroscope was used, combined with a Zeiss AxioCam MRc5 microscope camera. The habitus photographs were taken with a NIKON D5600 with a sigma 105 mm macrolens and a Canon EOS 5D digital camera using a Canon zoom lens EF 28–90 mm F 4–5.6 with three combined Hama Close-Up lenses 1, 2 + 4×.

### ﻿Measurements

**Figures 4–7.**
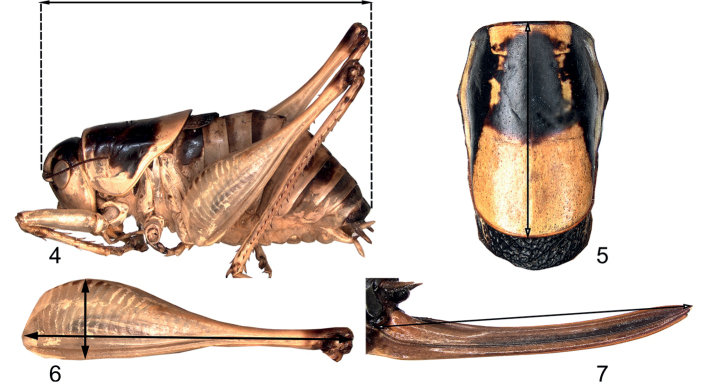
Measurements **4** body length **5** pronotum length **6** hind femur length and width **7** ovipositor length.

Figs 4–7 illustrate how measurements of body parts were taken, and the results are presented in Tables 6, 7. As bodies tend to be more or less swollen when kept in alcohol or shrunken after having been kept in spirit, measurements of body size tend to show a larger variation than, for instance, those of the pronotum or hind femur.

### ﻿Specimens

A list summarising all known localities, specimens, and repositories is presented in Suppl. material 1. Specimens examined to describe new species or the female sex are also mentioned in the taxonomic treatment. Suppl. material 2 lists all specimens studied for this paper including details about their use for field or stacked images, DNA, sound recording, and measurements.

### ﻿Repositories and acronyms

**BMNH** British Museum of Natural History, London, UK;

**CH** collection Klaus-Gerhard Heller, Triesdorf, Germany;

**CMUP** collection Bruno Massa, University of Palermo, Palermo, Italy;

**CT** collection Jos Tilmans, Wassenberg-Rothenbach, Germany;

**IBER**Institute of Biodiversity and Ecosystem Research, Bulgarian Academy of Sciences, Sofia, Bulgaria;

**MfNB** Museum für Naturkunde, Berlin, Germany;

**MHNG**Natural History Museum Geneva, Switzerland;

**NHMC**Natural History Museum Crete, University of Crete, Iraklion, Greece;

**obs.** observation (specimen not collected);

**RMNH**Naturalis Biodiversity Center, Leiden, The Netherlands.

## ﻿Results

Table 2 and Fig. 1 summarise new locations for *Eupholidoptera* from Crete and its adjoining islands discovered between 1987 and 2020 as presented in this paper. Apart from two species new to science and the first records of *E.smyrnensis* from Crete, the total number of records increased fivefold, largely due to the bycatches from the trapping program.

**Figures 8–10.**
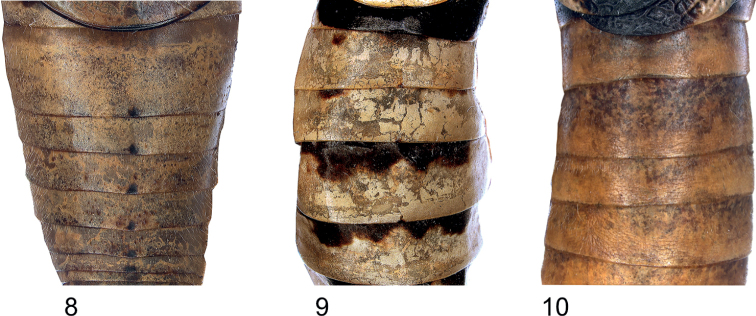
Colour pattern abdomen from above **8***Eupholidopterasmyrnensis* ♀ Makrigiannis RMNH.INS152908 **9***Eupholidopteragemellata* ♀ Mt. Idhi FC1651 RMNH.INS1141844 **10***Eupholidopteraastyla* ♂ Krotos RMNH5086990.

**Table 2.** Published and unpublished records (period 1987–2020) of *Eupholidoptera* from Crete and adjoining islands.

	Altitudinal range (m)	Published locations (Table 1)	Tilmans 1987–2019	Trapping program 1987–2019	Odé 2014	Observations 2012–2020	Heller 2016	Willemse and Zacharopoulou 2017	Chobanov et al. 2018	Tilmans and Willemse 2019	Total
*E.annamariae*	5–550	2	5	3	0	0	0	2	2	0	14
*E.astyla*	5–1850	10	6	24	0	0	1	3	1	0	45
*E.cretica*	1165–1234	1	0	4	0	0	0	0	0	0	5
*E.feri*	1100	1	0	0	0	0	0	0	0	0	1
*E.forcipata*	1350–2225	3	2	3	0	0	0	0	0	0	8
*E.francisae* sp. nov.	1–835	0	2	1	0	0	0	2	2	9	16
*E.gemellata*	1650–1910	1	0	1	0	0	0	0	0	0	2
*E.giuliae*	0–525	3	4	9	0	7	1	3	1	4	32
*E.jacquelinae*	30–270	1	0	8	0	1	0	0	0	0	10
*E.latens*	20–1815	7	3	4	1	0	1	1	2	2	21
*E.mariannae*	0–1475	4	2	9	0	0	0	2	0	0	17
*E.marietheresae* sp. nov.	1715	0	0	1	0	0	0	0	0	0	1
*E.pallipes*	1600–2440	1	0	1	0	1	0	0	0	0	3
*E.smyrnensis*	25–340	0	0	1	0	0	0	1	0	1	3
Total		34	23	69	1	9	3	14	8	16	178

### ﻿Key to *Eupholidoptera* species from Crete, Gavdos, Gavdopoula, and Andikithira

**Table:** 

1	Frons with tiger-pattern (Fig. 11); hind margin of abdominal tergites at the centre with tiny black dot (Fig. 8); male cercus with basal tooth (Fig. 111); hind margin of female subgenital plate forming two narrowly pointed lobes (Fig. 39) (west and central Crete)	***smyrnensis***
–	Frons with isolated dark spots or black crossband (Figs 12–24); abdominal tergite unicolourous, hind margin without a central black dot (Fig. 10) or with black edge (Fig. 9); male cercus unarmed or with subbasal tooth (Figs 112–125); hind margin of female subgenital plate different (Figs 40–52)	**2**
2	Females	**3**
–	Males	**15**
3	Subgenital plate with pair of elongate concavities divided by median ridge, apical lobes touching (Fig. 46) (Lasithi)	***annamariae***
–	Subgenital plate convex or proximally concave or with pits, apical lobes separated (Figs 40–45, 47–52)	**4**
4	Gavdos or Gavdopoula	***jacquelinae***
–	Crete or Andikithira	**5**
5	Subgenital plate wider to much wider than long (Figs 40, 42, 45, 50, 51)	**6**
–	Subgenital plate as wide as long or elongated (Figs 41, 43, 44, 47–49, 52)	**10**
6	Hind margin of subgenital plate with wide and deep median excision (Fig. 42) (Mt. Lefka)	***cretica***
–	Hind margin of subgenital plate differently formed (Figs 40, 45, 50, 51)	**7**
7	Hind margin of subgenital plate medially concave, without excision (Fig. 40) (Mt. Idi)	***gemellata***
–	Hind margin of subgenital plate with distinct excision (Figs 45, 50, 51)	**8**
8	Subgenital plate with apical lobes rounded (Fig. 45), in profile its apex (measured in a straight line parallel to the ovipositor) not reaching or surpassing proximal half of gonangulum (Fig. 67) (Andikithira and Chania) (in some females from Chania the subgenital plate is as wide as long or even elongated; see Taxonomic treatment and Discussion)	***francisae* sp. nov.**
–	Subgenital plate with apical lobes pointed (Figs 50, 51), in profile its apex (measured in a straight line parallel to the ovipositor) reaching or surpassing distal half of gonangulum (Fig. 68) (Mt. Idi or Mt. Dikti)	**9**
9	Subgenital plate 2.0× wider than long, proximal pits small, widely separated (Fig. 50) (Mt. Idi)	***forcipata***
–	Subgenital plate 1.4× wider than long, proximal pits large, closer to each other (Fig. 51) (Mt. Dikti)	***marietheresae* sp. nov.**
10	Front of head with large black patches (Fig. 13) (Mt. Lefka)	***pallipes***
–	Front of head with black dots (Figs 15–17, 19–21)	**11**
11	Apex subgenital plate in profile (measured in a straight line parallel to the ovipositor) not reaching or surpassing proximal half of gonangulum (Fig. 67) (Chania)	***francisae* sp. nov.**
–	Apex subgenital plate in profile (measured in a straight line parallel to the ovipositor) reaching or surpassing distal half of gonangulum (Fig. 68)	**12**
12	Subgenital plate proximally convex, flattened or slightly concave (Fig. 47)	***astyla***
–	Subgenital plate proximally with one wide or two separate pits (Figs 43, 44, 48, 49)	**13**
13	Apical lobes of subgenital plate not well produced, rectangular (Fig. 43); black patch front half pronotal disc absent, small or large, if present hind edge acutely V to U-shaped (Fig. 29)	***latens***
–	Apical lobes of subgenital plate more produced, rectangular to acute (Figs 44, 48, 49); front half pronotal disc with black patch, its hind edge widely V-shaped to transverse (Figs 30, 34, 35)	**14**
14	Subgenital plate proximally with two distinct pits separated by a median keel (Fig. 44); central black patch front half pronotal disc well delimited by pale edges, hind margin widely V-shaped (Fig. 30) (Chania and Rethimno)	***giuliae***
–	Subgenital plate proximally with a single or two pits (Figs 48, 49); front half pronotal disc mostly black, hind margin transverse (Figs 34, 35) (west and central Lasithi including Mt. Dikti)	***mariannae***
–	(Mt. Dikti, Katharo plains)	***feri***
15	Styli pointing backwards (Figs 169–171, 179–181)	**16**
–	Styli pointing downward or inward (Figs 172–178)	**21**
16	Cercus with subbasal side tooth (Figs 112, 113); subgenital plate with a short curved spine at base of each stylus (Figs 169, 170)	**17**
–	Cercus unarmed (Figs 114, 123–125); subgenital plate without spines (Figs 171, 179, 180) or with a very long straight spine at base of each stylus (Fig. 181)	**18**
17	Pronotum pale (Fig. 27); apical arms titillator completely fused, tip rounded at either side with a tiny thorn (Fig. 184) (Mt. Lefka)	***pallipes***
–	Pronotum with black band or patches (Fig. 26); apical arms fused, in apical quart split, tip truncated, unarmed (Fig. 183) (Mt. Idi)	***gemellata***
18	Styli very long, 5–6× longer than wide (Figs 171, 181)	**19**
–	Styli short, 2× longer than wide (Figs 179, 180)	**20**
19	Subgenital plate with long spine at base of stylus (Fig. 181); apical arms titillators separate in apical half (Fig. 197); anal tergite with hind margin with two large triangular extensions with V-shaped medial excision (Fig. 96) (Gavdos and Gavdopoula)	***jacquelinae***
–	Subgenital plate without a spine at base of stylus (Fig. 171); apical arms titillators separate in apical quarter (Fig. 185); anal tergite with hind margin forming two small, widely separated, triangular lobes (Fig. 86) (Mt. Lefka)	***cretica***
20	Hind margin of anal tergite from the cercus downward straight, side flaps gradually narrowing (Fig. 94); cerci narrower, 4–5× longer than wide, straight or very weakly curved inward (Fig. 123); apical arms titillator ending in two evenly curved, slender hooks (Fig. 195) (Mt. Idi)	***forcipata***
–	Hind margin of anal tergite from the cercus downward S-curved, side flaps first widening before narrowing (Fig. 95); cerci wider, 3–4× longer than wide, curved inward (Fig. 124); apical arms titillator ending in two very slender curved hooks, near the apex curved inward (Fig. 196) (Mt. Dikti)	***marietheresae* sp. nov.**
21	Anal tergite distally extended into two very long, spined hooks pointing downward (Fig. 90); apical arms titillator narrow, completely fused ending in needle shaped tip, pointing barely left or right (Figs 190, 206) (Lasithi)	***annamariae***
–	Anal tergite distally extended into short pointed lobes (Figs 87–89, 91–93); apical arms titillator in basal half wide or widening (Figs 186–189, 202–205) or narrow but tips not completely fused (Figs 191–194, 207–209)	**22**
22	Pointed lobes of anal tergite close together (Figs 91–93)	**23**
–	Pointed lobes of anal tergite widely separated (Figs 87–89)	**25**
23	Cercus unarmed (Fig. 120); apical arms titillator strongly asymmetrical (Figs 191, 207) (central Crete)	***astyla***
–	Cercus with subbasal side tooth (Figs 121, 122); apical arms titillator asymmetrical or barely so (Figs 193, 194, 208, 209) (east Crete)	**24**
24	Cercus conical (Fig. 121); pointed lobes anal tergite pointing backward (Fig. 92); (Katharo plain, Mt. Dikti)	***feri***
–	Cercus flattened and widened proximally (Fig. 122); pointed lobes of anal tergite pointing forward (Fig. 93) (west and central Lasithi including Mt. Dikti)	***mariannae***
25	Subgenital plate as wide as long (Fig. 145) with styli pointing inward (Fig. 173) (in Chania and Apokoronas subgenital plates are longer with styli pointing downwards; see species treatment and discussion)	***giuliae***
–	Subgenital plate elongate, tapering toward the apex (Figs 144, 146) with styli pointing downward (Figs 172, 174)	**26**
26	Tips apical lobes subgenital plate with a tooth (Figs 146, 160, 174) (on Andikithira tooth always present, in west and southwest Chania tooth sometimes missing or only on one apical lobe present), subgenital plate much longer than wide (mean ratio length-width 1.66)	***francisae* sp. nov.**
–	Tips apical lobes subgenital plate always without a tooth (Figs 144, 158, 172), subgenital plate longer than wide (mean ratio length-width 1.44) (north and central Chania)	***latens***

### ﻿Taxonomic treatment (species in alphabetical order)

The taxonomic treatment contains short diagnostics, illustrated with stacked images, for all species until now reported from Crete and its adjoining islands. New taxa as well as previously unknown sexes are described in more detail.

#### ﻿ Eupholidoptera annamariae

Taxon classificationAnimaliaOrthopteraTettigoniidae

Nadig, 1985

D77A6979-92F4-5A58-A9CD-BC4EE3A1313A

Figs 18 , 32 , 46 , 60 , 76 , 90 , 104 , 118 , 119 , 133 , 147 , 161 , 175 , 190 , 206 , 240 , 254 , 256 , 259 , Tables 1 , 2 , 5 , 6 , 7 , 10 , Suppl. materials 1 , 2 , 3 , 4

Eupholidoptera annamariae Nadig 1985: 329.
Morphological description. Nadig 1985: 329.
Bioacoustics. Çiplak et al. 2009: 27, 54.

##### Examined specimens.

1 ♂, 1 ♀ (**paratypes**); 23 ♂, 24 ♀ (for details see Suppl. material 2).

##### Diagnostic features.

Frontal part of head (Fig. 18) pale with black dots; frontal half of pronotal disc (Fig. 32) predominantly black sharply transversely delineated from pale rear half. Male (Fig. 240) – stridulatory file with 109 teeth (89 in Nadig 1985) (including proximal and distal ones), density of teeth in middle two thirds of the file 19 teeth per mm; anal tergite (Figs 76, 90, 104) with hind margin medially strongly bent downward forming two very long, curved teeth covered with small denticles pointing downward and slightly outward; cerci (Figs 118, 133) unarmed, 5× longer than wide, basal half cylindrical, apical half conical, slightly curved inward halfway subtly widened, in profile straight; subgenital plate (Figs 147, 161) wider than long, widest halfway, sides widely rimmed, in profile strongly upturned, pointing upward, tip apical lobes rounded, spineless, excised along one third of length; styli (Fig. 175) short, ca. one third as long as cerci, 1.5–2.0× longer than wide, inserted ventrally just proximal of tip of apical lobe, pointing downward; titillator (Figs 190, 206) slightly asymmetrical, apical arms strong, narrow, fused along entire length, smooth, evenly curved, apically narrowed toward needle shaped tip, pointing somewhat left or right. Female – subgenital plate (Figs 46, 60) circular to transverse oval with central longitudinal hump, adjoined by deep grooves, apical lobes touching, hind margin medially excised along one quarter to one third of length, in profile deltoid, with deep ventral groove, tip obtuse angular.

**Figures 11–24.**
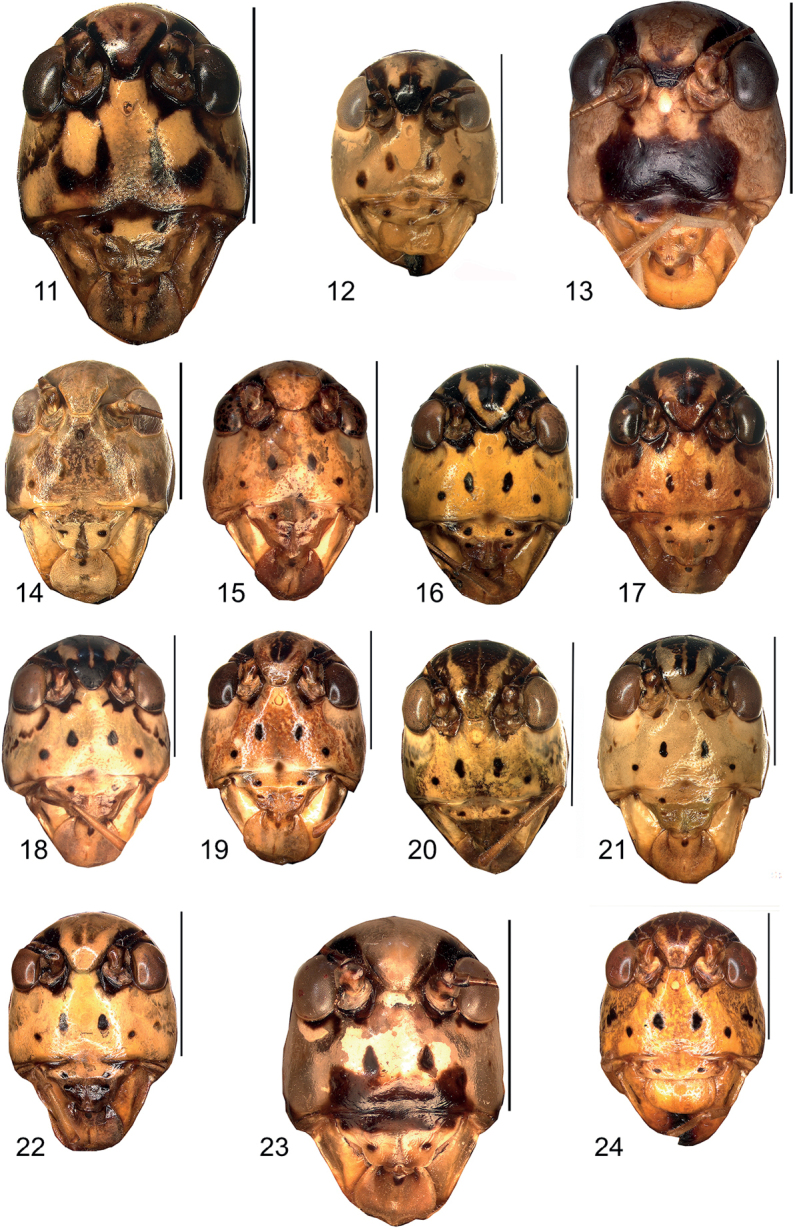
Colour pattern of head, frontal view **11***Eupholidopterasmyrnensis* ♀ Makrigiannis RMNH5014918 **12***Eupholidopteragemellata* ♂ Mt. Idhi RMNH.INS1141843 **13***Eupholidopterapallipes* ♂ paratype Mt. Lefka RMNH.INS1105313 **14***Eupholidopteracretica* ♂ Mt. Lefka, Omalos RMNH.INS114838 **15***Eupholidopteralatens* ♀ Mt. Lefka, Kalergi CT1987.047.04 **16***Eupholidopteragiuliae* ♂ Argoules CT1995.011.18 **17***Eupholidopterafrancisae* sp. nov. ♀ allotype Andikithira CT 2002.004.11 **18***Eupholidopteraannamariae* ♀ Kato Zakros CT1995.020.03 **19***Eupholidopteraastyla* ♀ Ano Viannos CT1987.024.03 **20***Eupholidopteraferi* ♂ holotype Mt. Dikti, Katharo plain RMNH.INS1105297 **21***Eupholidopteramariannae* ♀ Ag. Ioannis RMNH.5014906 **22***Eupholidopteraforcipata* ♂ Mt. Idhi CT1987.044.03 **23***Eupholidopteramarietheresae* sp. nov. ♂ paratype Mt. Dikti, FC1606 CT2000.096.01 **24***Eupholidopterajacquelinae* ♂ holotype Gavdos CT2000.005.02. Scale bars: 5 mm.

**Figures 25–38.**
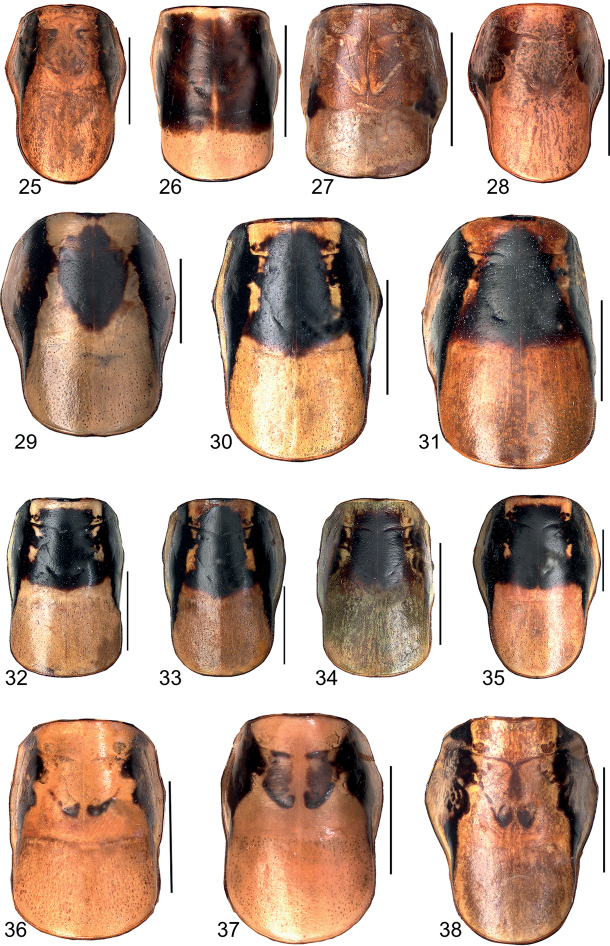
Colour pattern of pronotum, dorsal view **25***Eupholidopterasmyrnensis* ♂ Makrigiannis RMNH.INS152909 **26***Eupholidopteragemellata* ♂ Mt. Idhi, Amariou RMNH.INS1141843 **27***Eupholidopterapallipes* ♂ paratype Mt. Lefka RMNH.INS1105313 **28***Eupholidopteracretica* ♂ Mt. Lefka, Omalos RMNH.INS1141838 **29***Eupholidopteralatens* ♂ Rhodopos CH8236 **30***Eupholidopteragiuliae* Chora Sfakion ♂ CT2000.014.10 **31***Eupholidopterafrancisae* sp. nov. ♂ holotype Andikithira CT2002.004.04 **32***Eupholidopteraannamariae* ♂ Kato Zakros CT2000.030.04 **33***Eupholidopteraastyla* ♂ Krotos RMNH.INS1141819 **34***Eupholidopteraferi* ♂ holotype Mt. Dikti, Katharo plain RMNH.INS1105297 **35***Eupholidopteramariannae* ♀ Kalavros RMNH.5086974 **36***Eupholidopteraforcipata* ♂ Mt. Idhi CT1987.044.03 **37***Eupholidopteramarietheresae* sp. nov. ♂ holotype Mt. Dikti RMNH.INS1141850 **38***Eupholidopterajacquelinae* ♂ holotype Gavdos CT2000.005.02. Scale bars: 5 mm.

**Figures 39–52.**
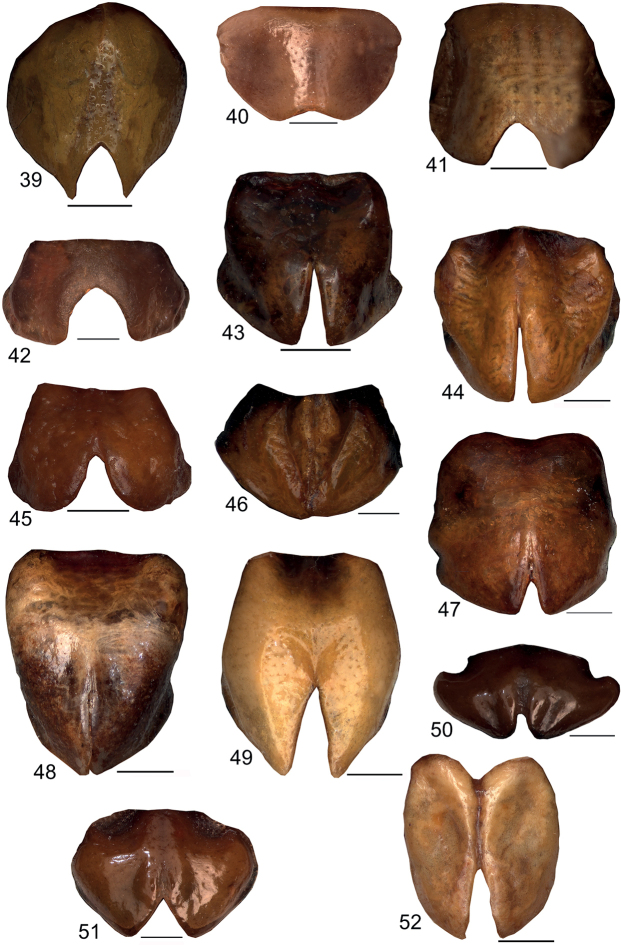
Female subgenital plate in ventral view **39***Eupholidopterasmyrnensis* Makrigiannis RMNH.5014918 **40***Eupholidopteragemellata* Mt. Idhi, FC1602 CT2000.095.02 **41***Eupholidopterapallipes* allotype Mt. Lefka RMNH.INS1105312 **42***Eupholidopteracretica* Mt. Lefka, Omalos RMNH.INS1141837 **43***Eupholidopteralatens* Mt. Lefka, Kalergi CT1987.047.04 **44***Eupholidopteragiuliae* Argoules CT1995.011.03 **45***Eupholidopterafrancisae* sp. nov. allotype Andikithira CT2002.004.11 **46***Eupholidopteraannamariae* Kato Zakros CT1995.020.03 **47***Eupholidopteraastyla* Krotos RMNH.INS1141820 **48***Eupholidopteraferi* allotype Mt. Dikti, Katharo plain RMNH.INS1105298 **49***Eupholidopteramariannae* Kalavros RMNH5014912 **50***Eupholidopteraforcipata* Mt. Idhi, FC1602 RMNH.INS1141845 **51***Eupholidopteramarietheresae* sp. nov. allotype Mt. Dikti RMNH.INS1141849 **52***Eupholidopterajacquelinae* Gavdos allotype CT2001.004.13. Scale bars: 1 mm.

**Figures 53–66.**
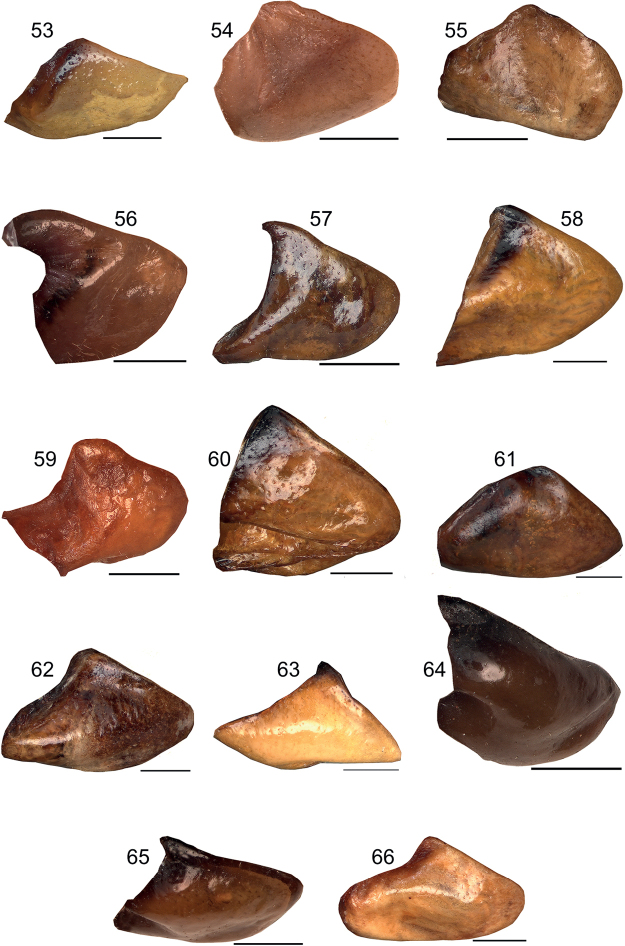
Female subgenital plate in lateral view **53***Eupholidopterasmyrnensis* Makrigiannis RMNH.5014918 **54***Eupholidopteragemellata* Mt. Idhi, FC1602 CT2000.095.02 **55***Eupholidopterapallipes* allotype Mt. Lefka RMNH.INS1105312 **56***Eupholidopteracretica* Mt. Lefka, Omalos RMNH.INS1141837 **57***Eupholidopteralatens* Mt. Lefka, Kalergi CT1987.047.04 **58***Eupholidopteragiuliae* Argoules CT1995.011.03 **59***Eupholidopterafrancisae* sp. nov. allotype Andikithira CT2002.004.11 **60***Eupholidopteraannamariae* Kato Zakros CT1995.020.03 **61***Eupholidopteraastyla* Krotos RMNH.INS1141820 **62***Eupholidopteraferi* allotype Mt. Dikti, Katharo plain RMNH.INS1105298 **63***Eupholidopteramariannae* Kalavros RMNH5014912 **64***Eupholidopteraforcipata* Mt. Idhi, FC1602 RMNH.INS1141845 **65***Eupholidopteramarietheresae* sp. nov. allotype Mt. Dikti RMNH.INS1141849 **66***Eupholidopterajacquelinae* allotype Gavdos CT2001.004.13. Scale bars: 1 mm.

**Figures 67, 68.**
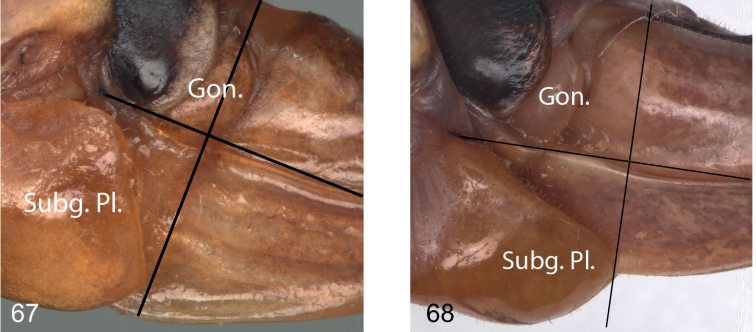
Female subgenital plate in lateral view **67***Eupholidopterafrancisae* sp. nov. Anidhroi CT2001.002.07 **68***Eupholidopteralatens* Prases DC-Ort000512.

##### Measurements.

See Tables 6, 7.

##### Bioacoustics.

Based upon the sound recordings of 6 specimens (53 syllables measured), the song of *E.annamariae*, as in all species of *Eupholidoptera*, consists of isolated syllables produced in long series with the opening hemisyllable much shorter and weaker than the closing hemisyllable. In *E.annamariae*, the syllable duration is ~ 160 ms. In the present recordings, the syllable repetition rate is very low. Published records (Çiplak et al. 2009) show a syllable duration of ~ 128 ms at 25 °C and a syllable repetition rate of 1/s at maximum. The song may most likely be confused with the other species of *Eupholidoptera* in Crete, except *E.smyrnensis* and *E.forcipata*. For details of sound recordings of *Eupholidopteraannamariae* see Suppl. material 3.

##### Variation.

The colour pattern and genitalia in *E.annamariae* in the specimens studied show little variation with one exception. In one of four males collected along the northern coast of Lasithi, west of Sitia, near Xerokampos the cerci showed a clearly developed inner tooth, halfway the cercus (Fig. 119). Other morphological traits in this aberrant male as well as in the other three males from Xerokampos fitted *E.annamariae*. The cercus with a side tooth is considered an anomaly. It is noteworthy that the location where the aberrant male of *E.annamariae* was found is the northwestern most location of *E.annamariae* only some 5 km away from Kalavros where *E.mariannae* was found.

##### Differential diagnosis.

Males differ from congenerics in the anal tergite (Figs 76, 90, 104) with the uniquely shaped long, curved spined teeth pointing downward and inward, in the stout, unarmed almost straight cercus (Figs 118, 133), in the wide, upturned subgenital plate (Figs 147, 161) lacking spines, pre-apically inserted with short, downward pointing styli (Fig. 175) and in the narrow, completely fused slightly asymmetrical apical arms of the titillator (Figs 190, 206). Females distinctly differ in the subgenital plate (Figs 46, 60) with a central hump bordered by deep and wide semi-circular grooves. From all Cretan *Eupholidoptera* species, the black part in the anterior half of the pronotum is most pronounced in *E.annamariae*. For more details differentiating *E.annamariae* from other Cretan *Eupholidoptera* see Table 5.

**Figures 69–82.**
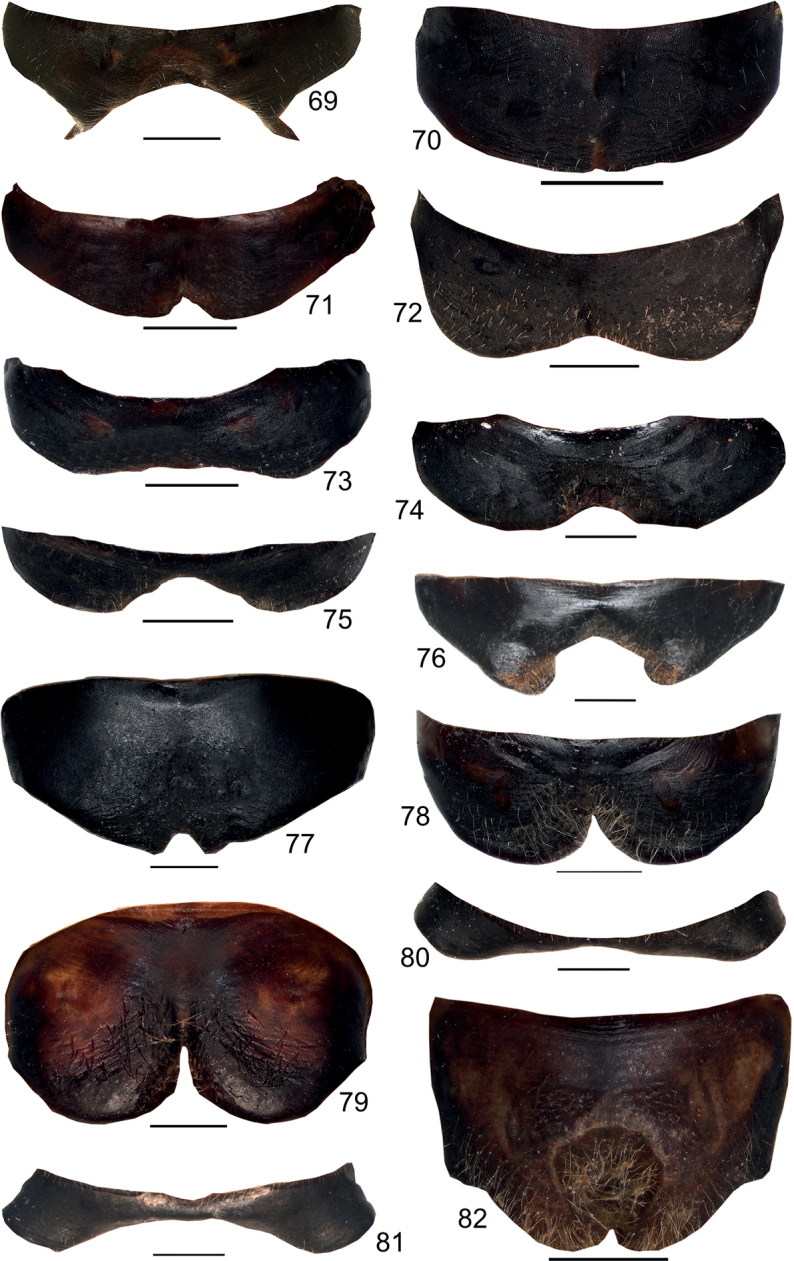
Male anal tergite in dorsal view **69***Eupholidopterasmyrnensis* Makrigiannis RMNH.INS152909 **70***Eupholidopteragemellata* holotype Mt. Idhi RMNH.INS1105300 **71***Eupholidopterapallipes* paratype Mt. Lefka RMNH.INS1105313 **72***Eupholidopteracretica* Mt. Lefka, Omalos FC17807 RMNH.INS1141838 **73***Eupholidopteralatens* Mt. Lefka, Kalergi CT2003.009.01 **74***Eupholidopteragiuliae* Chora Sfakion CT2000.024.04 **75***Eupholidopterafrancisae* sp. nov. holotype Andikithira CT2002.004.04 **76***Eupholidopteraannamariae* Kato Zakros CT2000.030.04 **77***Eupholidopteraastyla* Krotos RMNH.INS1141819 **78***Eupholidopteraferi* holotype Mt. Dikti, Katharo plain RMNH.INS 1105297 **79***Eupholidopteramariannae* holotype Anatoli RMNH.INS1105311 **80***Eupholidopteraforcipata* Mt. Idhi CT1987.044.01 **81***Eupholidopteramarietheresae* sp. nov. paratype Mt. Dikti FC1606 CT2000.096.01 **82***Eupholidopterajacquelinae* holotype Gavdos CT2000.005.02. Scale bars: 1 mm.

**Figures 83–96.**
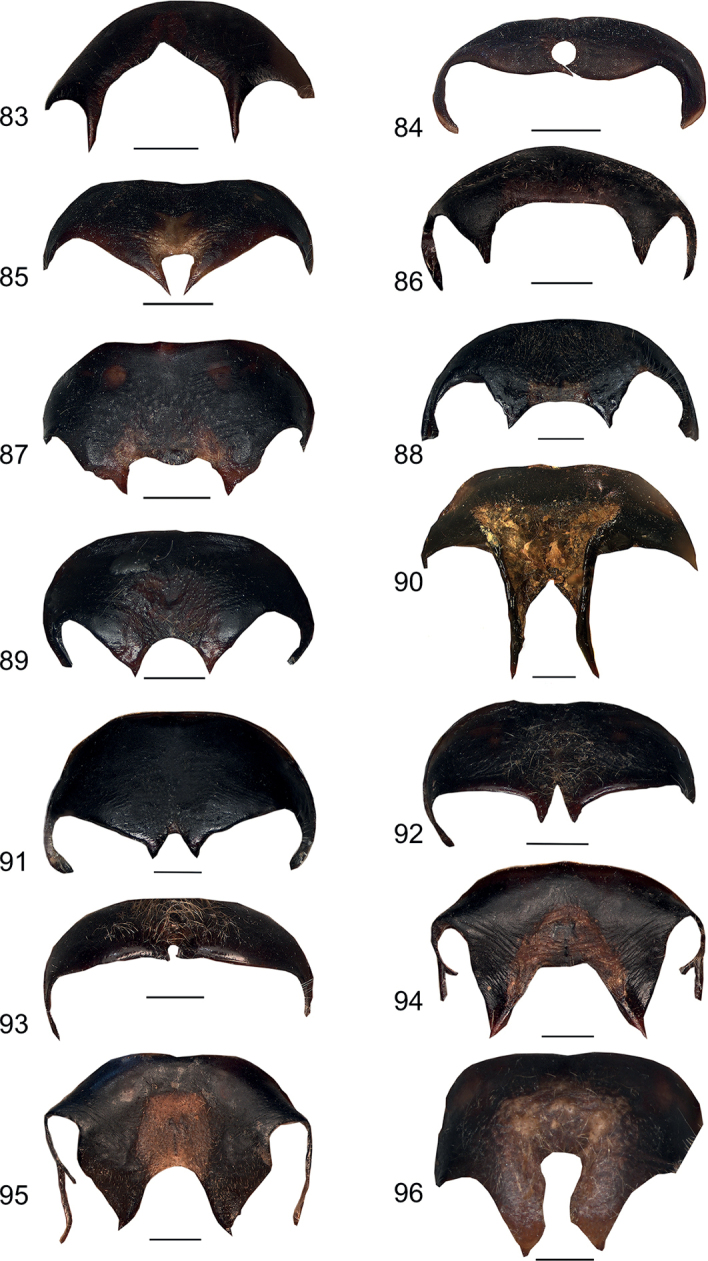
Male anal tergite in caudal view **83***Eupholidopterasmyrnensis* Makrigiannis RMNH.INS152909 **84***Eupholidopteragemellata* Mt. Idhi CT2000.095.01 **85***Eupholidopterapallipes* paratype Mt. Lefka RMNH.INS1105314 **86***Eupholidopteracretica* Mt. Lefka, Omalos FC17807 RMNH.INS1141838 **87***Eupholidopteralatens* Mt. Lefka, Kalergi CT2003.009.01 **88***Eupholidopteragiuliae* Chora Sfakion CT2000.014.01 **89***Eupholidopterafrancisae* sp. nov. paratype Andikithira CT2002.004.02 **90***Eupholidopteraannamariae* Kato Zakros CT2000.030.01 **91***Eupholidopteraastyla* Krotos RMNH.INS1141819 **92***Eupholidopteraferi* holotype Mt. Dikti, Katharo plain RMNH.INS1105297 **93***Eupholidopteramariannae* Prina FC17798 RMNH.INS1141840 **94***Eupholidopteraforcipata* Mt. Idhi CT1987.044.01 **95***Eupholidopteramarietheresae* sp. nov. paratype Mt. Dikti FC1606 CT2000.096.01 **96***Eupholidopterajacquelinae* paratype Gavdos NHMC2001.004.12. Scale bars: 1 mm.

**Figures 97–110.**
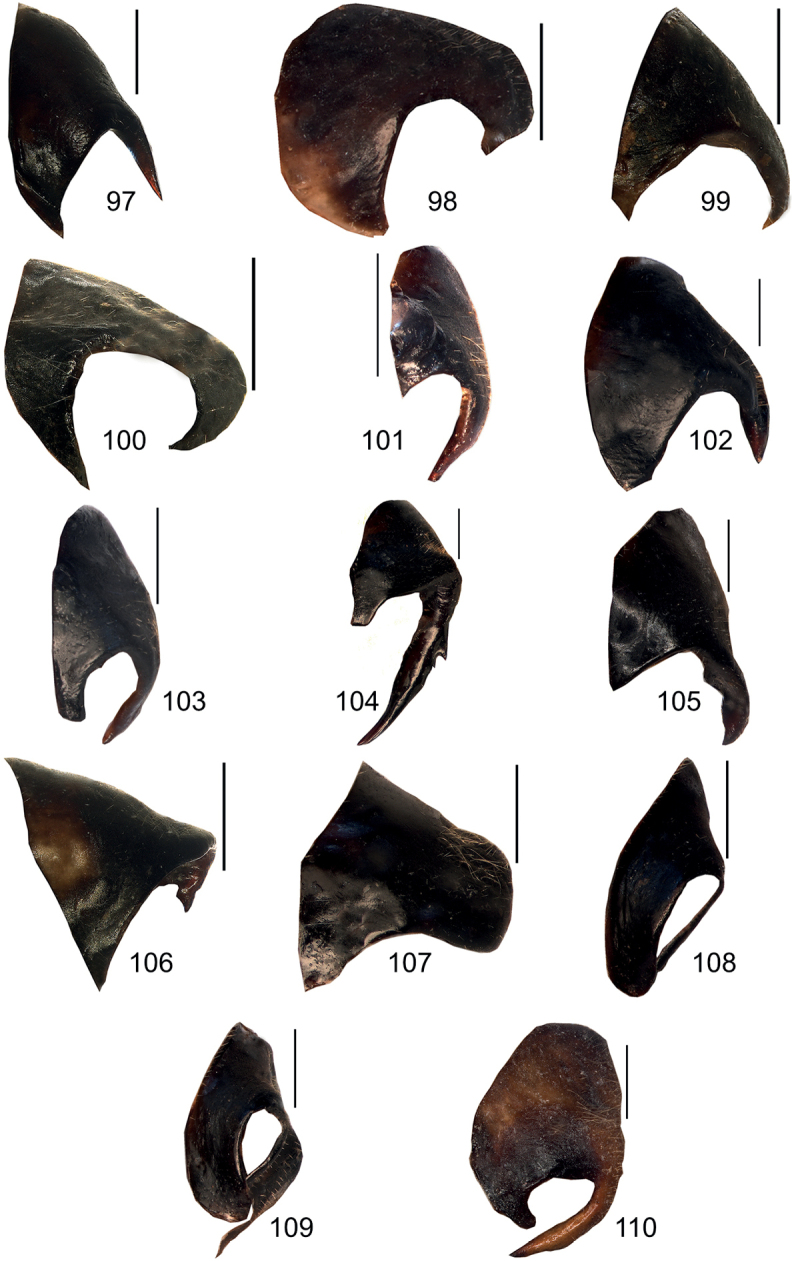
Male anal tergite in lateral view **97***Eupholidopterasmyrnensis* Makrigiannis RMNH5087053 **98***Eupholidopteragemellata* Mt. Idhi CT2000.095.01 **99***Eupholidopterapallipes* paratype Mt. Lefka RMNH.INS1105314 **100***Eupholidopteracretica* Mt. Lefka, Omalos FC17807 RMNH.INS1141838 **101***Eupholidopteralatens* Mt. Lefka, Kalergi CT1987.047.01 **102***Eupholidopteragiuliae* Argoules CT1995.011.11 **103***Eupholidopterafrancisae* sp. nov. paratype Andikithira CT2002.004.02 **104***Eupholidopteraannamariae* Kato Zakros CT2000.030.03 **105***Eupholidopteraastyla* Ano Viannos CT1995.024.01 **106***Eupholidopteraferi* holotype Mt. Dikti, Katharo plain RMNH.INS1105297 **107***Eupholidopteramariannae* Kalavros CT2017.029.01 **108***Eupholidopteraforcipata* Mt. Idhi CT1987.044.01 **109***Eupholidopteramarietheresae* sp. nov. paratype Mt. Dikti FC1606 CT2000.096.01 **110***Eupholidopterajacquelinae* Gavdopoula CT1996.019.01. Scale bars: 1 mm.

**Figures 111–125.**
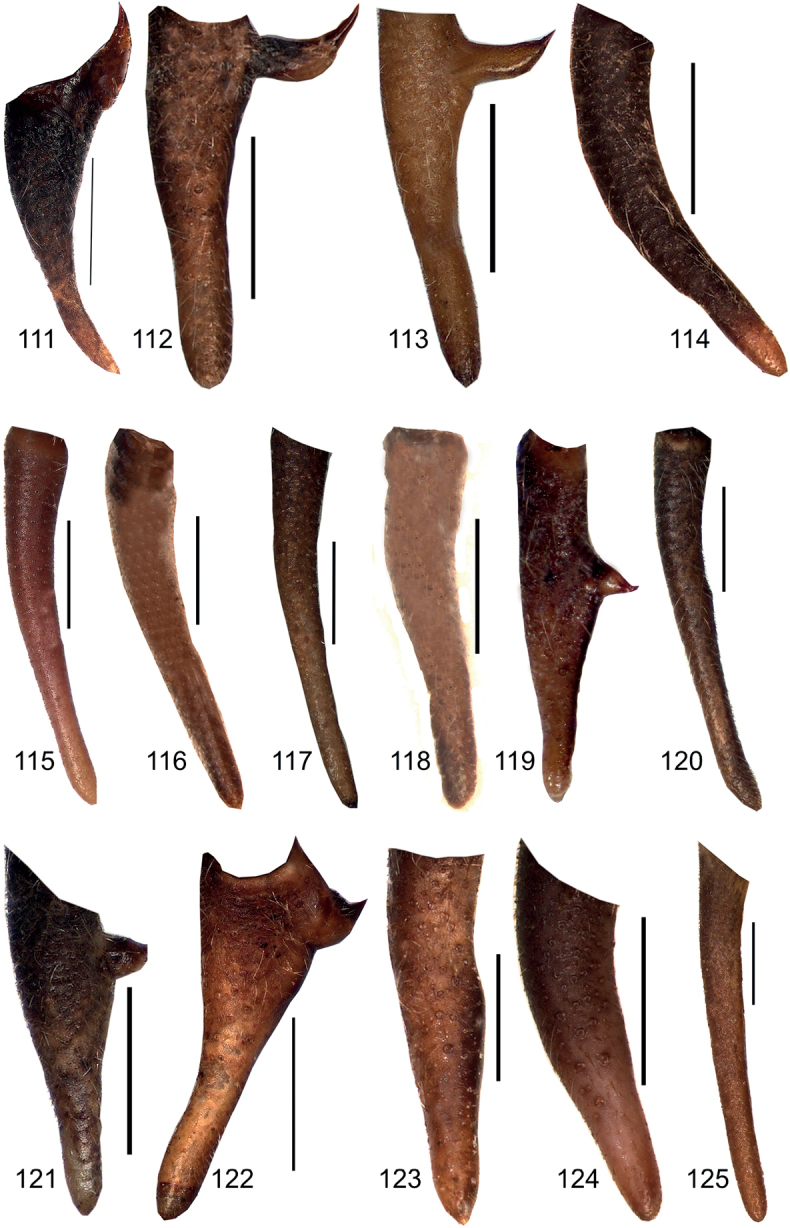
Male cercus in dorsal view **111***Eupholidopterasmyrnensis* Makrigiannis RMNH5087053 **112***Eupholidopteragemellata* Mt. Idhi FC1651 RMNH.INS1141843 **113***Eupholidopterapallipes* Mt. Lefka paratype RMNH.INS1105314 **114***Eupholidopteracretica* Mt. Lefka, Omalos FC17807 RMNH.INS1141838 **115***Eupholidopteralatens* Xiloskalo IBER DC-Ort000558 **116***Eupholidopteragiuliae* Chora Sfakion CT2000.014.02 **117***Eupholidopterafrancisae* sp. nov. holotype Andikithira CT2002.004.04 **118***Eupholidopteraannamariae* Kato Zakros CT2000.030.03 **119***Eupholidopteraannamariae* Xerokampos, male with anomalous cercus, IBER DC-Ort000565 **120***Eupholidopteraastyla* Ierapetra MfN s.n. **121***Eupholidopteraferi* holotype Mt. Dikti, Katharo plain RMNH.INS1105297 **122***Eupholidopteramariannae* holotype Anatoli RMNH.INS1105311 **123***Eupholidopteraforcipata* Mt. Idhi CT1987.044.01 **124***Eupholidopteramarietheresae* sp. nov. holotype Mt. Dikti FC1606 RMNH.INS1141850 **125***Eupholidopterajacquelinae* holotype Gavdos CT2000.005.02. Scale bars: 1 mm.

**Figures 126–139.**
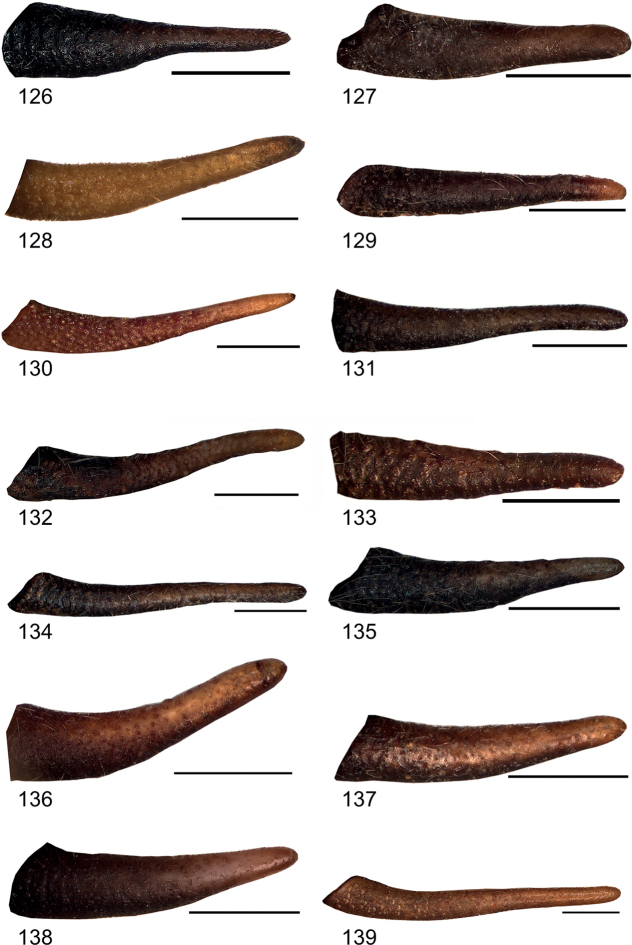
Male cercus in lateral view **126***Eupholidopterasmyrnensis* Makrigiannis RMNH.5087053 **127***Eupholidopteragemellata* Mt. Idhi FC1651 RMNH.INS1141843 **128***Eupholidopterapallipes* paratype Mt. Lefka RMNH.INS1105314 **129***Eupholidopteracretica* Mt. Lefka, Omalos FC17807 RMNH.INS1141838 **130***Eupholidopteralatens* Mt. Lefka Kalergi CT2003.009.01 **131***Eupholidopteragiuliae* Chora Sfakion CT2000.024.04 **132***Eupholidopterafrancisae* paratype sp. nov. Andikithira CT2002.004.08 **133***Eupholidopteraannamariae* Kato Zakros CT1995.020.02 **134***Eupholidopteraastyla* paratype Ierapetra MfN s.n. **135***Eupholidopteraferi* holotype Mt. Dikti, Katharo plain RMNH.INS1105297 **136***Eupholidopteramariannae* holotype Anatoli RMNH.INS1105311 **137***Eupholidopteraforcipata* Mt. Idhi CT1987.044.01 **138***Eupholidopteramarietheresae* sp. nov. holotype Mt. Dikti FC1606 RMNH.INS1141850 **139***Eupholidopterajacquelinae* holotype Gavdos CT2000.005.02. Scale bars: 1 mm.

**Figures 140–153.**
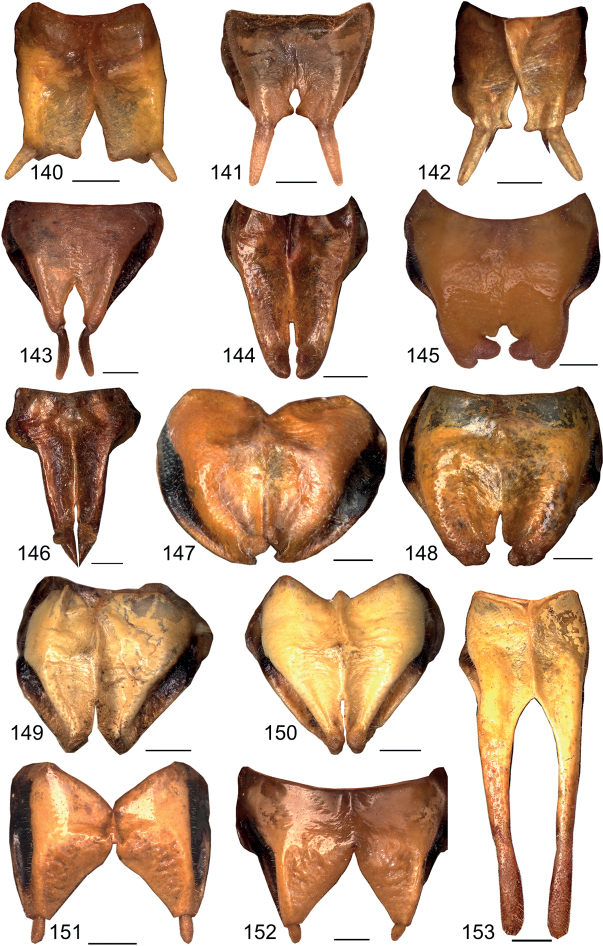
Male subgenital plate in ventral view **140***Eupholidopterasmyrnensis* Lagkada RMNH 5106270 **141***Eupholidopteragemellata* Mt. Idhi FC1651 RMNH.INS1141843 **142***Eupholidopterapallipes* paratype Mt. Lefka RMNH.INS1105313 **143***Eupholidopteracretica* Mt. Lefka, Omalos FC17807 RMNH.INS1141838 **144***Eupholidopteralatens Mt. Lefka Kalergi* CT1987.047.02 **145***Eupholidopteragiuliae* Skaloti IBER DC-Ort000564 **146***Eupholidopterafrancisae* sp. nov. holotype Andikithira CT2002.004.04 **147***Eupholidopteraannamariae* Kato Zakros CT2000.030.04 **148***Eupholidopteraastyla* Krotos RMNH.INS1141819 **149***Eupholidopteraferi* holotype Mt. Dikti, Katharo plain RMNH.INS1105297 **150***Eupholidopteramariannae* holotype Anatoli RMNH.INS1105311 **151***Eupholidopteraforcipata* Mt. Idhi CT1987.044.03 **152***Eupholidopteramarietheresae* sp. nov. holotype Mt. Dikti FC1606 RMNH.INS1141850 **153***Eupholidopterajacquelinae* holotype Gavdos 2000.005.02. Scale bars: 1 mm.

**Figures 154–167.**
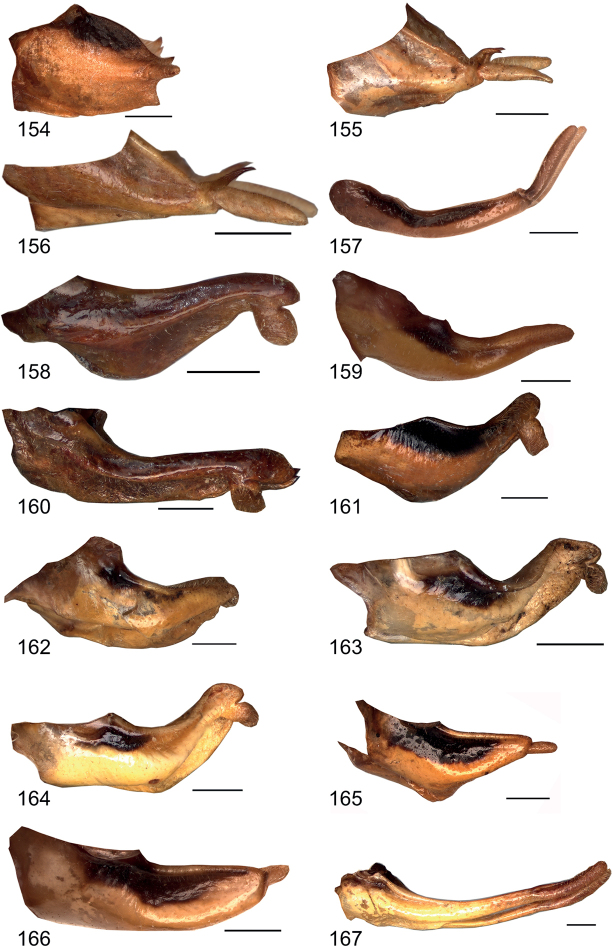
Male subgenital plate in lateral view **154***Eupholidopterasmyrnensis* Lagkada RMNH.INS152909 **155***Eupholidopteragemellata* holotype Mt. Idhi RMNH.INS1105300 **156***Eupholidopterapallipes* paratype Mt. Lefka RMNH.INS1105313 **157***Eupholidopteracretica* Mt. Lefka, Omalos FC17807 (RMNH.INS1141838 **158***Eupholidopteralatens* Mt. Lefka Kalergi CT2003.009.01 **159***Eupholidopteragiuliae* Skaloti IBER DC-Ort000564 **160***Eupholidopterafrancisae* sp. nov. holotype Andikithira CT2002.004.04 **161***Eupholidopteraannamariae* Kato Zakros CT2000.030.04 **162***Eupholidopteraastyla* Krotos RMNH5086992 **163***Eupholidopteraferi* holotype Mt. Dikti, Katharo plain RMNH.INS1105297 **164***Eupholidopteramariannae* holotype Anatoli RMNH.INS1105311 **165***Eupholidopteraforcipata* Mt. Idhi CT1987.044.03 **166***Eupholidopteramarietheresae* sp. nov. holotype Mt. Dikti FC1606 RMNH.INS1141850 **167***Eupholidopterajacquelinae* holotype Gavdos CT2000.005.02. Scale bars: 1 mm.

**Figures 168–181.**
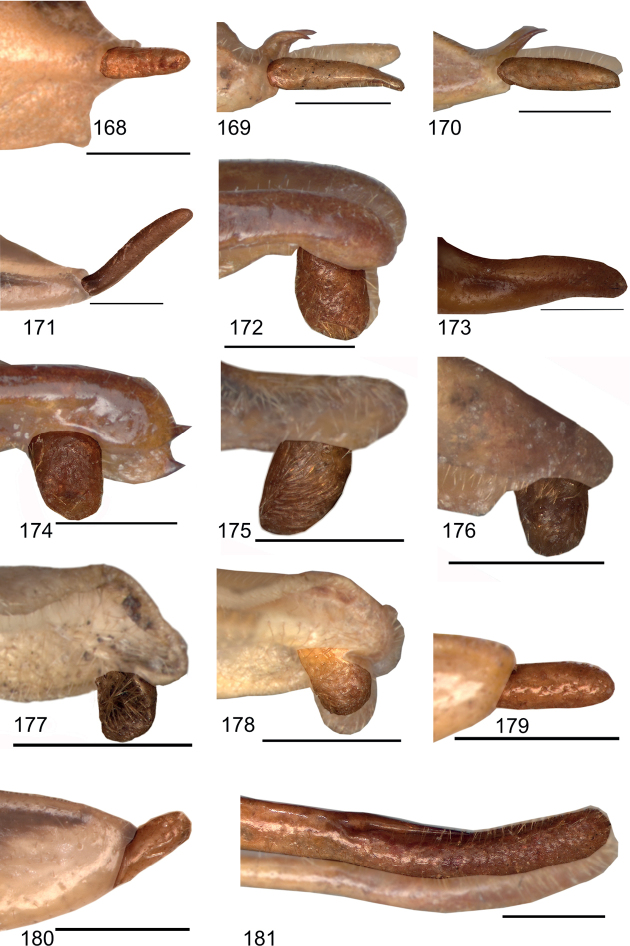
Male stylus in lateral view **168***Eupholidopterasmyrnensis* Makrigiannis RMNH.INS152909 **169***Eupholidopteragemellata* holotype Mt. Idhi RMNH.INS1105300 **170***Eupholidopterapallipes* paratype Mt. Lefka RMNH.INS1105313 **171***Eupholidopteracretica* Mt. Lefka, Omalos FC17807 RMNH.INS1141838 **172***Eupholidopteralatens* Mt. Lefka Kalergi CT2003.009.01 **173***Eupholidopteragiuliae* Skaloti IBER DC-Ort000564 **174***Eupholidopterafrancisae* sp. nov. paratype Andikithira CT2002.004.08 **175***Eupholidopteraannamariae* Kato Zakros CT2000.030.04 **176***Eupholidopteraastyla* Krotos RMNH.INS1141819 **177***Eupholidopteraferi* holotype Mt. Dikti, Katharo plain RMNH.INS1105297 **178***Eupholidopteramariannae* holotype Anatoli RMNH.INS1105311 **179***Eupholidopteraforcipata* Mt. Idhi CT1987.044.03 **180***Eupholidopteramarietheresae* sp. nov. holotype Mt. Dikti FC1606 RMNH.INS1141850 **181***Eupholidopterajacquelinae* holotype Gavdos CT2000.005.02. Scale bars: 1 mm.

**Figures 182–197.**
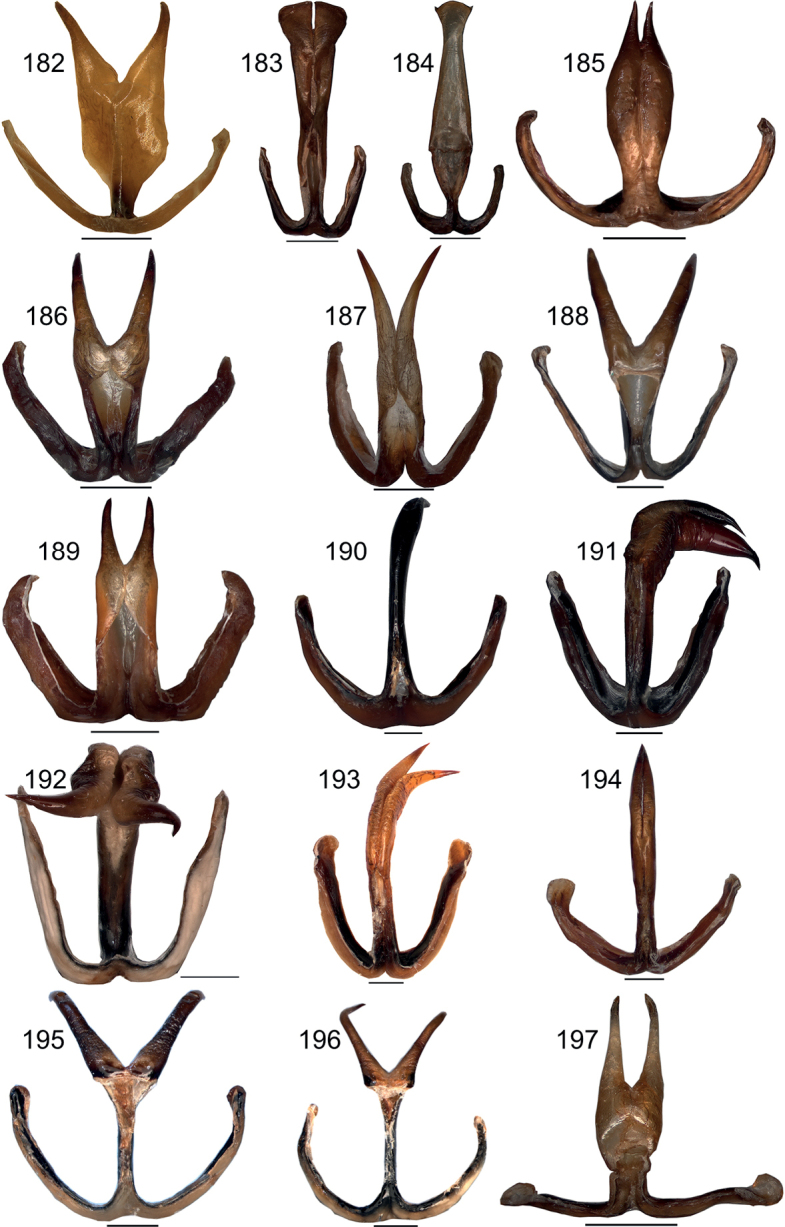
Male titillator in ventral view **182***Eupholidopterasmyrnensis* Makrigiannis RMNH5087053 **183***Eupholidopteragemellata* Mt. Idhi Amariou FC1651 RMNH.INS1141843 **184***Eupholidopterapallipes* paratype Mt. Lefka RMNH.INS1105314 **185***Eupholidopteracretica* Mt. Lefka, Omalos FC17807 RMNH.INS1141838 **186***Eupholidopteralatens* Mt. Lefka Kalergi CT2003.009.01 **187***Eupholidopteralatens* Rhodopos CH 8236 **188***Eupholidopteragiuliae* Argoules CT1995.011.11 **189***Eupholidopterafrancisae* sp. nov. paratype Andikithira CT2002.004.08 **190***Eupholidopteraannamariae* Kato Zakros CT2000.030.01 **191***Eupholidopteraastyla* Krotos RMNH.INS1141819 **192***Eupholidopteraastyla* Kofinas FC460 RMNH.INS1141836 **193***Eupholidopteraferi* holotype Mt. Dikti, Katharo plain RMNH.INS1105297 **194***Eupholidopteramariannae* paratype Malles CH2906A **195***Eupholidopteraforcipata* Mt. Idhi CT2019.047.01 **196***Eupholidopteramarietheresae* sp. nov. paratype Mt. Dikti FC1606 CT2000.096.01 **197***Eupholidopterajacquelinae* holotype Gavdos CT2000.005.02. Scale bars: 1 mm.

**Figures 198–212.**
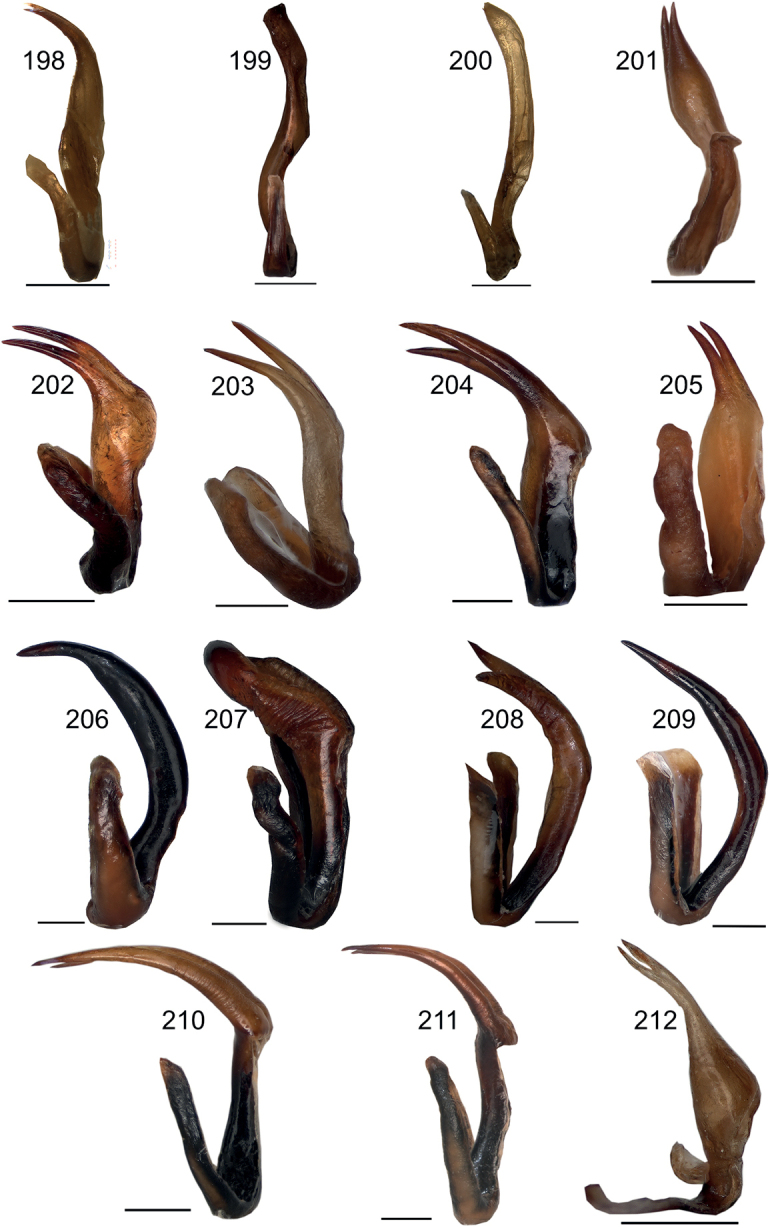
Male titillator in lateral view **198***Eupholidopterasmyrnensis* Makrigiannis RMNH5087053 **199***Eupholidopteragemellata* Mt. Idhi Amariou FC1651 RMNH.INS1141843 **200***Eupholidopterapallipes* paratype Mt. Lefka RMNH.INS1105314 **201***Eupholidopteracretica* Mt. Lefka, Omalos FC17807 RMNH.INS1141838 **202***Eupholidopteralatens* Mt. Lefka Kalergi CT2003.009.01 **203***Eupholidopteralatens* Rhodopos CH 8236 **204***Eupholidopteragiuliae* Argoules CT1995.011.11 **205***Eupholidopterafrancisae* sp. nov. paratype Andikithira CT2002.004.08 **206***Eupholidopteraannamariae* Kato Zakros CT2000.030.01 **207***Eupholidopteraastyla* Krotos RMNH.INS1141819 **208***Eupholidopteraferi* holotype Mt. Dikti, Katharo plain RMNH.INS1105297 **209***Eupholidopteramariannae* Kalavros CT2017.029.01 **210***Eupholidopteraforcipata* Mt. Idhi CT1987.044.01 **211***Eupholidopteramarietheresae* sp. nov. holotype Mt. Dikti FC1606 RMNH.INS1141850 **212***Eupholidopterajacquelinae* holotype Gavdos CT2000.005.02. Scale bars: 1 mm.

##### Distribution.

The species was described from Kato Zakros along the eastern coast of Crete. After its original finding it was collected again in the same area between Kato Zakros and Zakros (Çiplak et al. 2009). New data presented here show that the range extends from the southeastern coast east of Kalo Nero Bay along the entire east coast up into the northernmost peninsular and along the northern coast westward up to and beyond Sitia (Fig. 254). It is still unclear if the species also occurs more inland east of the Koutsouras-Sitia road. For a complete list of localities, specimens and repositories see Suppl. material 1.

##### Habitat.

The species has been found in sparse phrygana between sea level and 550 m in dry open terrains with bare ground, covered with small spiny or thorny shrublets in which it hides during the day. The species was also found in a pitfall trap in sand dunes near Xerokampos along the southeastern coast.

##### Phenology.

Hand catches of this species were made between end of May and mid-August (25/05–15/08). This roughly coincides with the period during which the species was caught in pitfall traps. Still their presence may be more prolonged into August or up to October as a trap sampled 12 October 2000 and set 6 August still contained nine adults.

#### ﻿ Eupholidoptera astyla

Taxon classificationAnimaliaOrthopteraTettigoniidae

(Ramme, 1927)

A68D7726-DAAD-531A-A150-9B7841DAF3DF

Figs 10 , 19 , 33 , 47 , 61 , 77 , 91 , 105 , 120 , 134 , 148 , 162 , 176 , 191 , 192 , 207 , 213 , 224 , 225 , 241 , 254 , 256 , 259 , Tables 1 , 2 , 5 , 6 , 7 , 9 , 10 , Suppl. materials 1 , 2 , 3 , 4

Pholidoptera astyla Ramme 1927: 133.
Eupholidoptera astyla (Ramme, 1927); Ramme 1951: 198.
Morphological description. Ramme 1927: 133; 1939: 100; Harz 1969: 377.
Bioacoustics. Çiplak et al. 2009: 27, 54–55.

##### Examined specimens.

1 ♂ (**paratype**); 81 ♂, 59 ♀ (for details see Suppl. material 2).

##### Diagnostic features.

Frontal part of head (Fig. 19) pale with black dots; frontal half of pronotal disc (Fig. 33) with more or less extensive black patch, border with pale rear half transverse or V-shaped. Male (Fig. 241) – stridulatory file with 101–105 teeth (including proximal and distal ones), density of teeth in middle two thirds of the file 22–24 teeth per mm; anal tergite (Figs 77, 91, 105) with hind margin toward middle forming two small pointed teeth separated by a short narrow V-shaped excision, tips pointing downwards; cerci (Figs 120, 134) unarmed, 5–7× longer than wide, weakly conical, weakly curved inward in basal half, in profile straight; subgenital plate (Figs 148, 162) wider than long, widest halfway, sides rimmed, in profile upturned, tip apical lobes narrowly truncate, spineless, at inner side emarginate with V-shaped excision along one fifth of length; styli (Fig. 176) minute, circular, inserted ventrally, proximal of tip of apical lobe, pointing inward to downward; titillator (Figs 191, 207) asymmetrical, apical arms widening from base, halfway splitting into two strongly thickened, flattened and wrinkled arms, forming wide to almost straight angle ending into two very strong curved spine-like teeth, pointing left or right.

##### Redescription of female.

In 1927 *E.astyla* was described based on a single male from Naxos and three females and the male abdomen from Crete (Ramme 1927). According to Willemse and Heller (2001) the presence of *E.astyla* on Naxos is doubtful. An extensive search on Naxos in June 2019 by the first author only revealed *E.smyrnensis* at midlevel altitudes in the north-eastern part of the island but no other *Eupholidoptera* species. Willemse and Heller (2001) indicated that the *E.astyla* females listed in the original description (Ramme 1927: 134) from Ierapetra, Anatoli, and Kato Chorio could in fact belong to *E.mariannae* which they described in their paper from this area. In 2001 female *E.mariannae* were not known and this assumption could neither be confirmed nor denied. Based on the discovery of male and female *E.mariannae* in neighbouring locations near Kavousi and Kalavros, opportunity has been taken to re-examine paratypes used by Ramme (1927) in his description of *E.astyla* and compare them with female *E.astyla* and *E.mariannae* described in this paper. Unfortunately, the female from “Jerapetra” could not be located in the MfNB. Comparison of the subgenital plates (Figs 214, 215) revealed that females from Kato Chorio and Anadoli resemble female *E.mariannae* from Kalavros and Kavousi rather than *E.astyla*. This implies that Ramme based his description of female *E.astyla* on female *E.mariannae*. Consequently female *E.astyla* has still not been described and is redescribed here based on specimens from Rethimno and Iraklion.

##### Description.

**Female.**

##### Examined specimens.

11 ♀: RETHIMNO: Idhi Mt., Idhaio Andro -1987.041.02 (CT); Idhi Mt., Ski-centre – 1987.046.03 (CT); Nea Kria Vrisi, 2 km NW – 2000.016.04 (CT); IRAKLION: Ano Viannos, 3 km SE – 1995.024.03 (CT), RMNH.5014909 (RMNH), RMNH.5086970 (RMNH), RMNH 5086971 (RMNH); Krotos, 0.5 km N – RMNH.INS1141820 (RMNH); Marathos – RMNH.5086989 (RMNH); Mournia, 3 km SW – 2001.008.04 (CT); Tsoutsouros, 6 km NNW – 1995.014.03 (CT). For more details see Suppl. material 2.

General appearance (Figs 224, 225) and colouration as male. First abdominal segment dorsally black, laterally lighter; remaining segments dorsally yellowish brown, sides lighter coloured, last segment completely black. Elytra in dorsal view covered by pronotum, in profile barely protruding, light coloured. Cercus short, conical tapering, more so in apical third toward a pointed tip, straight to slightly upturned in profile, straight to slightly curved inward in dorsal view, covered with pale short and long hairs. Subgenital plate (Figs 47, 61) oblong, greatest width halfway or in distal quarter, in ventral view convex, proximally flattened to slight depressed, halfway on side with or without a (in-)distinct bulge, surface shiny, smooth with dispersed hairs, hind margin converging gradually from halfway or more abruptly in distal quarter, with medial V-shaped excision along one third of length, corners rectangular to sharp-angled, in profile triangular to trapezoid with a more or less distinct dorsal depression, lower edge straight distally slightly upturned, tip obtuse angular. Ovipositor almost straight to slightly upcurved, 1.5–2.0× longer than pronotum.

**Figures 213–215.**
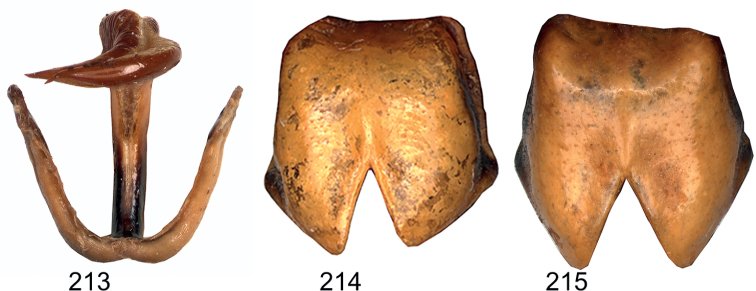
*Eupholidopteraastyla* paratypes **213** titillator in dorsal view *Eupholidopteraastyla* ♂ paratype Ierapetra s.n. **214** subgenital plate in ventral view *Eupholidopteramariannae* ♀ Anadoli s.n. [paratype of *Eupholidopteraastyla*] **215***Eupholidopteramariannae* ♀ Kato Chorion s.n. [paratype of *Eupholidopteraastyla*].

##### Measurements.

See Tables 6, 7.

##### Bioacoustics.

Based upon the sound recordings of two specimens (20 syllables measured), the song of *E.astyla*, as in all species of *Eupholidoptera*, consists of isolated syllables produced in long series with the opening hemisyllable much shorter and weaker than the closing hemisyllable. In *E.astyla*, the syllable duration is ~ 120 ms, with a syllable rate up to ~ 1/s. Published records (Çiplak et al. 2009) show a syllable duration of ~ 166 ms at 25 °C and a syllable repetition rate of less than 1/s. The song may most likely be confused with the other species of *Eupholidoptera* in Crete, except *E.smyrnensis* and *E.forcipata*. For details of sound recordings of *Eupholidopteraastyla* see Suppl. material 3.

##### Variation.

High altitude specimens are smaller than specimens found at lower altitudes. Variation in black and pale colour patterns seem linked to individuals rather than to populations. Cercus more or less slender and more or less bent inward. Medial excision anal tergite V- to U-shaped, adjoining teeth aligned with dorsal surface pointing distally or bent downward, pointing downward. Subgenital plate more or less compact, in profile, lower margin evenly rounded or with an angle halfway. Styli minute to small, in the west and north pointing inward, toward the south and east pointing downward, exceptionally also outward. In some males from Asterousia Mt. (central-south Crete), styli were lacking almost completely. Titillator can be more or less compact, apical arms parallel or slightly divergent, apical teeth gradually or suddenly and more strongly pointed, pointing right or left. Two males (of 34) collected in pitfall traps near Kofinas along the south coast showed an almost symmetrical titillator, the two apical arms pointing in opposite directions (Fig. 192). In other characters these two males fully matched *E.astyla*, as did all other males from Kofinas. The symmetrical titillators are considered individual anomalies. The shape of the female subgenital plate varies: in the west and at higher altitudes on Mts. Idi and Dikti being plump with a short median excision, toward the east changing to oblong more distinctly acutely bilobed with a deeper excision, resembling those of *E.feri* and *E.mariannae*.

##### Differential diagnosis.

Males differ from congenerics in the strongly asymmetrical, thickened and wrinkled apical arms of the titillator (Figs 191, 207) pointing left or right, in the narrow V-shaped excision in the anal tergite (Figs 77, 91, 105) with tips pointing downward, in the slender, unarmed weakly inward curved cerci (Figs 120, 134), in the wide, upturned, spineless subgenital plate (Figs 148, 162) and in the minute, pre-apically inserted styli pointing downward (Fig. 176). Females differ in the oblong subgenital plate (Figs 47, 61), proximally convex with a short excision. In the southeast toward Lasithi female subgenital plates are longer, the apical lobes on both sides of the medial excision more pointed, resembling *E.mariannae*. In colouration, particularly the anterior half of the pronotum *E.astyla* resembles *E.annamariae*, *E.feri*, *E.giuliae*, and *E.mariannae*. For more details differentiating *E.astyla* from other Cretan *Eupholidoptera* see Table 5.

##### Distribution.

From the Cretan species of *Eupholidoptera*, *E.astyla* has the widest range. Current data indicate its range covers large parts of central Crete, stretching from central and eastern Rethimno to western Lasithi, from Skaleta east of the city of Rethimno in the northwest to Ierapetra along the southern coast in the southeast (Fig. 254). Despite the additional localities presented here, the exact boundaries in the west, where it meets *E.giuliae*, and in the east, where it meets *E.mariannae* and *E.feri*, are not clear. Current information suggests *E.astyla* to be absent from large parts of the central lowlands south of Iraklion and the northern coastal region east of Iraklion but this certainly requires additional investigation. Opportunity was taken to examine (and photograph) the male mentioned by Ramme (1927) in his description of *E.astyla* of which only the last part of the abdomen was left. Based on the shape of the titillator (Fig. 213) this male clearly belongs to *E.astyla*. The label of this male only states “Jerapetra”. It is not clear whether the name “Jerapetra” actually refers to the town Ierapetra or the district. Either way, assuming the label is correct, the presence in Ierapetra indicates there is an area west of Ierapetra where both *E.astyla* and *E.mariannae* may occur together (Fig. 254). For a complete list of localities, specimens and repositories see Suppl. material 1.

##### Habitat.

The species habitats cover a wide altitudinal range: from sea level along the northern and southern coast to 1550–1800 m on Mt. Idi and Mt. Dikti. Pitfall traps that caught *E.astyla* were placed in sparse to dense phrygana, maquis and areas dominated by pine trees.

##### Phenology.

Hand catches indicate adults can be found from early May onward at lower altitudes up to the end of August at higher altitudes. Pitfall trap catches indicate that especially at higher altitudes the species can be found at least up to the second half of September or even early October.

#### ﻿ Eupholidoptera cretica

Taxon classificationAnimaliaOrthopteraTettigoniidae

Ramme, 1951

953D4F3C-FE4D-5230-A2A9-308EAC95DAD5

Figs 14 , 28 , 42 , 56 , 72 , 86 , 100 , 114 , 129 , 143 , 157 , 171 , 185 , 201 , 226 , 227 , 255 , Tables 1 , 2 , 5 , 6 , 7 , 9 , 10 , Suppl. materials 1 , 2

Eupholidoptera cretica Ramme, 1951: 202.
Morphological description. Ramme 1951: 202.

##### Remark.

*Eupholidopteracretica* was described after a single male, collected 13 June 1942 by K. Zimmermann. This, most likely, is the mammologist who worked on the mammals of Crete and published a review of his observations including a map (Zimmermann et al. 1953). As collecting location for the specimen Ramme (1951) mentioned “Sanmaria” [sic]. Samaria is the name used both for the gorge as well as the hamlet in the gorge ca. 4 km inland at 340 m altitude. Extensive searches in 2000, 2011, and 2019 around Agia Roumeli, the coastal village at the entrance of the gorge and the Samaria gorge itself were unsuccessful. Likewise visits to the Omalos plain above the Samaria gorge in 1991 (Heller), 2003 and 2004 (Tilmans) failed to find the species. Then the species appeared to have been trapped in fermenting traps placed in bushes at 1200 m on the southeastern-most flanks of Mt. Lefka above the villages of Anopoli and Limnia in 1991 and again on the Omalos plateau just above the Samaria gorge in 2019. Only few specimens were caught. A single undamaged male and female have been used to make stacked images, present diagnostic features for the male and describe the female.

##### Examined specimens.

1 ♂, 2 ♀ (for details see Suppl. material 2).

##### Diagnostic features.

Frontal part of head (Fig. 14) pale with black dots; pronotum (Fig. 28) pale with more or less distinct black spots in centre of disc and along rear edge of side flap. Male – stridulatory file with 107 teeth (including proximal and distal ones), density of teeth in middle two thirds of the file 32 teeth per mm; anal tergite (Figs 72, 86, 100) with hind margin forming two widely separated triangular lobes pointing backward and downward with pointed tip; cerci (Figs 114, 129) unarmed, 5× longer than wide, basal half cylindrical, apical half conical, strongly curved inward, in profile straight; subgenital plate (Figs 143, 157) as slightly wider than long, proximally widest, apically gradually narrowing, sides partly rimmed, in profile very weakly upturned, pointing backward, tip apical lobes narrowly truncate, spineless, with wide V-shaped excision along one third of length; styli (Fig. 171) long, 0.6× as long as cerci, 6× longer than wide, cylindrical, inserted at inner tip of apical lobe, pointing backward and upward; titillator (Figs 185, 201) symmetrical, apical arms from base widening, in apical half narrowing again, swollen, fused except for straight tooth-like apical fifth part, in profile weakly S-shaped hardly widened in basal half, somewhat dilated in swollen apical half.

##### Description.

**Female.** Examined specimens. 2♀: CHANIA: Lefka Mt., above Omalos – RMNH.INS1141837 (RMNH); Lefka Mt., Sfakion above Anopoli – 2005.060.01 (CT) (for details see Suppl. material 2).

General appearance (Figs 226, 227) and colouration as male. Elytra in dorsal view covered by pronotum, in profile barely protruding, light coloured. Cerci relatively long, as long as subgenital plate, slightly bent inward and upward, conical, gradually narrowing toward slender pointed tip. Subgenital plate (Figs 42, 56) distinctly wider than long, greatest width in distal half, in ventral view medially convex, laterally flattened, halfway forming distinct bulge, surface dull, smooth with dispersed hairs, hind margin with very wide U-shape excision reaching along a quarter to halfway, corners rectangular, in profile rhomboid to deltoid with a distinct depression in apical and dorsal corner, lower edge strongly convex, tip obtuse angular. Ovipositor in proximal two thirds straight, apical third slightly curved upward, 2× longer than pronotum.

##### Measurements.

See Tables 6, 7.

##### Bioacoustics.

The song of this species has not yet been recorded.

##### Differential diagnosis.

Males differ from congenerics in the stout, unarmed, inward curved cercus (Figs 114, 129), in the widely separated triangular lobes of the anal tergite with tips pointing backward and downward (Figs 72, 86, 100), in the subgenital plate (Figs 143, 157) gradually narrowing into truncate and spineless tips, in the very long, apically inserted backward and upward pointing styli (Fig. 171) and the widened apical arms of the titillator (Figs 185, 201) fused except for two short straight teeth in apical fifth. Females differ in the wide, convex, subgenital plate (Figs 42, 56), the hind margin medially with a very wide and deep excision. In colouration *E.cretica* is one of the few Cretan *Eupholidoptera* species with no or only minute black marking on the pronotal disc. For more details differentiating *E.cretica* from other Cretan *Eupholidoptera* see Table 5.

##### Distribution.

Besides the type location which is not exactly traceable, only known from two spots on Mt. Lefka, one in northwest near the Omalos plateau and Samaria gorge and a second along the southeastern slopes above the villages of Anopoli and Limnia (Fig. 255). For a complete list of localities, specimens and repositories see Suppl. material 1.

##### Habitat.

The area around the Omalos plateau where the species was trapped is described as *Cupressus* forest. The species has been trapped in fermenting traps placed above the ground in shrubs, indicating *E.cretica* like *E.smyrnensis*, *E.mariannae* as well as *E.jacquelinae* but contrary to most other Cretan species, actually lives in such shrubs and not in small prickly bushes on the ground.

##### Phenology.

Still very little is known. The first male was caught on 13 June 1942. Specimens being caught in traps were found in traps operative between 31 July and 19 October. The recorded altitudes where the species was found are between 1165 m and 1235 m.

#### ﻿ Eupholidoptera feri

Taxon classificationAnimaliaOrthopteraTettigoniidae

Koçak & Kemal, 2010

DC0197EA-9045-5BD3-A74B-A5D5A6831CD9

Figs 20 , 34 , 48 , 62 , 78 , 92 , 106 , 121 , 135 , 149 , 163 , 177 , 193 , 208 , 255 , 259 , Tables 1 , 2 , 5 , 6 , 7 , 10 , Suppl. materials 1 , 2 , 3

Eupholidoptera rammei Willemse & Heller, 2001: 333.
Eupholidoptera feri Koçak & Kemal, 2010: 7.
Morphological description. Willemse and Heller 2001: 333–339.
Bioacoustics. Willemse and Heller 2001: 335, fig. 52 [as E.rammei]; Çiplak et al. 2009: fig. 234 [as E.rammei].

##### Examined specimens.

**Holotype, allotype** (for details see Suppl. material 2).

##### Diagnostic features.

Frontal part of head (Fig. 20) pale with black dots; frontal half of pronotal disc (Fig. 34) with extensive central black patch, border with pale rear half transverse to V-shaped. Male – stridulatory file with 100 teeth (Çiplak et al. 2009) (including proximal and distal ones), density of teeth in middle two thirds of the file 22–24 teeth per mm; anal tergite (Figs 78, 92, 106) with hind margin medially forming two small pointed teeth pointing downward, separated by a short narrow V-shaped excision; cerci (Figs 121, 135) armed, inner margin with short side-tooth at one third of length pointing inward, 5× longer than wide, basal half cylindrical, apical half conical, straight, in profile slightly upturned in apical third; subgenital plate (Figs 149, 163) slightly wider than long, widest in proximal third, sides widely rimmed, in profile upturned, tip apical lobes narrowly truncate, spineless, with slit-like excision along one fifth of length; styli (Fig. 177) minute, circular, flat, inserted at inner side of apical lobes, just proximal of tip, pointing downward; titillator (Figs 193, 208) slightly asymmetrical, greater part apical arms fused, in apical half transversely wrinkled diverging into two spines pointing sideways in different angles, in profile narrow, halfway slightly wider, curved upward, in apical half stronger so. Female – subgenital plate (Figs 48, 62) longer than wide, widest in proximal third, convex, proximally concave, apical lobes touching, tips acute with median excision along one third of length, in profile triangular.

##### Measurements.

See Tables 6, 7.

##### Bioacoustics.

Based upon the sound recordings of 1 specimen (10 syllables measured), the song of *E.feri*, as in all species of *Eupholidoptera*, consists of isolated syllables produced in long series with the opening hemisyllable much shorter and weaker than the closing hemisyllable. The syllable duration is ~ 271 ms, recorded at 15 °C, with a syllable rate up to ~ 1/s. Published records (Çiplak et al. 2009), based upon the same sound recording and after correction for the low temperature, show a syllable duration of ~ 121 ms at 25 °C and a syllable repetition rate of ~ 1/s at maximum. The song may most likely be confused with the other species of *Eupholidoptera* in Crete, except *E.smyrnensis* and *E.forcipata*. For details of sound recordings of *Eupholidopteraferi* see Suppl. material 3.

##### Differential diagnosis.

Males differ from congenerics in the stout, cylindrical cerci (Figs 121, 135) with a subbasal inner side tooth, in the anal tergite (Figs 78, 92, 106) medially not extended, bent downward with very narrow V-shaped excision, tips pointing downward, in the wide, upturned, spineless subgenital plate (Figs 149, 163) with minute, pre-apically inserted styli (Fig. 177) pointing downward and the narrow asymmetrical apical arms of the titillator (Figs 193, 208). Females differ in the elongated and proximally concave subgenital plate (Figs 48, 62), its apical lobes touching with an excision along one third of the length. In colouration the species resembles *E.annamariae*, *E.astyla*, and *E.mariannae*. For more details differentiating *E.feri* from other Cretan *Eupholidoptera* see Table 5.

##### Distribution.

Only known from the Katharo plain in the eastern offshoots of Mt. Dikti, in the western part of the Lasithi district (Fig. 255). For a complete list of localities, specimens and repositories see Suppl. material 1.

##### Habitat.

The type specimens were collected 1–2 m high in a *Quercus* shrub in the Katharo plain, part of which is used for cultivation (vineyards), the rest consists of bare grounds.

##### Phenology.

The Katharo plain lies at an altitude of 1100 m. The type specimens were collected in late August.

#### ﻿ Eupholidoptera forcipata

Taxon classificationAnimaliaOrthopteraTettigoniidae

Willemse & Kruseman, 1976

7ECFFF11-734E-5833-BBD2-91CE3C35954A

Figs 22 , 36 , 50 , 64 , 80 , 94 , 108 , 123 , 137 , 151 , 165 , 179 , 195 , 210 , 255 , 259 , Tables 1 , 2 , 5 , 6 , 7 , 9 , 10 , Suppl. materials 1 , 2 , 3

Eupholidoptera forcipata Willemse & Kruseman, 1976: 131.
Morphological description. Willemse and Kruseman 1976: 131–134.
Bioacoustics. Çiplak et al. 2009: 27, 51, 54.

##### Examined specimens.

**Holotype, allotype**, 8 ♂, 9 ♀ (**paratypes**); 12 ♂, 5 ♀ (for details see Suppl. material 2).

##### Diagnostic features.

Frontal part of head (Fig. 22) pale with black dots; pronotal disc (Fig. 36) pale with central black marking resembling an open “W” or frontal half with larger central black patch, border with pale rear half transverse or V-shaped; Male – stridulatory file with 193 teeth (190 in Çiplak et al. 2009) (including proximal and distal ones), density of teeth in middle two thirds of the file 36 teeth per mm (33 in Çiplak et al. 2009); anal tergite (Figs 80, 94, 108) narrow, distally strongly bent and extended downward forming two apical lobes ending in strong teeth pointing downward and slightly outward separated by a wide, deep excision; cerci (Figs 123, 137) unarmed, 4× longer than wide, basal half cylindrical, apical half conical straight, inner margin sinuate with minute bulge halfway, in profile slightly upturned in apical half; subgenital plate (Figs 151, 165) wider than long, widest halfway, sides widely rimmed, in profile upturned, tip apical lobes narrowly truncate, spineless, forming very wide V-shaped excision reaching halfway; styli (Fig. 179) short, 0.3 as long as cerci, 2× longer than wide, cylindrical, inserted at tip of apical lobes pointing backwards; titillator (Figs 195, 210) symmetrical, basal half apical arms fused, narrow, stalk-like, halfway widening, swollen, diverging into two evenly curved, slender hooks, in profile in basal half narrowing apically, wide angled with apical hooks, reaching or extending above anal tergite. Female – subgenital plate (Figs 50, 64) twice as wide as long, widest halfway, proximally with two distinct, widely separated pit-like concavities, apical lobes with depression, tips rounded, separated by U-shaped excision along one quarter of length, in profile oblong, lower edge distally strongly upcurved, tip truncated.

##### Measurements.

See Tables 6, 7.

##### Bioacoustics.

Based upon the sound recordings of one specimen (30 syllables measured), the song of *E.forcipata*, as in all species of *Eupholidoptera*, consists of isolated syllables produced in long series with the opening hemisyllable much shorter and weaker than the closing hemisyllable. In *E.forcipata*, the syllable duration is ~ 525 ms, with a syllable rate up to somewhat less than 1/s. The syllable duration easily discerns the song of this species from the other known songs of *Eupholidoptera* from Crete. Published records (Çiplak et al. 2009) show a syllable duration of ~ 425 ms and a syllable repetition rate far lower than 1/s. For details of sound recordings of *Eupholidopteraforcipata* see Suppl. material 3.

##### Differential diagnosis.

Males differ from congenerics in the pointed backward and downward extended widened apical lobes of the anal tergite (Figs 80, 94, 108) with tips pointing downward and slightly outward, in the wide upturned, spineless subgenital plate (Figs 151, 165) with a very wide V-shaped excision, in short, apically inserted styli (Fig. 179) pointing backward, in the stout, straight cerci (Figs 123, 137) with a minute bulge on the inner margin and the symmetrical apical arms of the titillator (Figs 195, 210), in basal half fused and narrow, in apical half strongly diverging hooks. Females differ in the very wide subgenital plate (Figs 50, 64), proximally with two concavities, tips rounded with U-shaped excision along quarter of the length. *Eupholidopteraforcipata* closely resembles *E.marietheresae* sp. nov. but male *E.forcipata* differ from male *E.marietheresae* sp. nov. in the straight hind margin of the anal tergite (compare Fig. 94 with Fig. 95), slimmer cercus (compare Fig. 123 with Fig. 124) and the apical arms of the titillator being straight in basal half and gradually upcurved in apical half (compare Fig. 195 with Fig. 196). In female *E.forcipata* the subgenital plate is shorter and the proximal pits being placed further apart than in *E.marietheresae* sp. nov. (compare Fig. 50 with Fig. 51). In colouration particularly the anterior half of the pronotum *E.forcipata* lacks extensive black markings or patches. For more details differentiating *E.forcipata* from other Cretan *Eupholidoptera* see Table 5.

##### Distribution.

Only known from higher altitudes on Mt. Psiloritis, central Crete (Fig. 255). For a complete list of localities, specimens and repositories see Suppl. material 1.

##### Habitat.

The species lives at high altitudes in subalpine phrygana in low prickly bushes (e.g., *Astragalus*) in which it hides during the day.

##### Phenology.

The species occurs between 1350 m and 2225 m. Adults have been collected by hand at the end of July and during the first half of August. Trap catches indicate they are still active up to September and possibly October.

#### ﻿ Eupholidoptera francisae

Taxon classificationAnimaliaOrthopteraTettigoniidae

Tilmans & Odé sp. nov.

2FE72D89-B51E-5D5B-93D9-A99BCB61DFE8

https://zoobank.org/6A1CA984-AD9D-4472-A6C3-3B8CF2100492

Figs 17 , 31 , 45 , 59 , 67 , 75 , 89 , 103 , 117 , 132 , 146 , 160 , 174 , 189 , 205 , 216–217 , 218 , 228–231 , 243–246 , 254 , 256 , 257 , 258 , 259 , Tables 2 , 3 , 4 , 5 , 6 , 7 , 8 , 10 , Suppl. materials 1 , 2 , 3 , 4

##### Remark.

The *Eupholidoptera* populations present on the island of Andikithira and in the area of western-southwestern Chania in Crete not only proved to differ from the geographically nearest other taxa of the genus: *Eupholidopteraspinigera*, restricted to the island of Kithira, and *Eupholidopteralatens* from northern and central Chania, but also from all its other congenerics. This new taxon is described below. For arguments to assign the *Eupholidoptera* populations of Andikithira and western/southwestern Chania populations as one single new taxon see under Discussion.

##### Examined specimens.

**Type specimens.** ♂ **holotype** (2002.004.04) (CT), ♀ **allotype** (2002.004.11) (CT), both labeled: HELLAS, Andikithira, 150 m, 9.V.2002/3 km S.E.S. Potamos/WGS 84 35°51.996'N, 023°18.114'E/legnt. J.M. Tilmans and J.F.R. Tilmans-Smid.

**Paratypes.** 8 ♂ & 5 ♀ (CT), 1 ♂ & 1 ♀ (NHMC), 1 ♂ & 1 ♀ (RMNH): same location and date as holotype; further paratypes 2 ♀ (CT): HELLAS, Andikithira, 50 m, 9.V.2002/0,6 km S.E.S. Potamos/WGS84 35°52.600'N, 023°17.426'E/legnt. J.M. Tilmans & J.F.R. Tilmans-Smid; 1 ♂ & 2 ♀ (CT): HELLAS, Andikithira, 50 m, 27.V.2008/0,6 km S.E.S. Potamos/WGS84 35°52.605'N, 023°17.439'E/legnt. J.M. Tilmans & J.F.R. Tilmans-Smid; 1♂ & 2♀ (RMNH): Greece – Crete (Chania): Ag. Paraskevi (Elafonisos-Maniatiana)/445 m; 17.VI.2019; 35.285645°N, 23.588774°E/leg. L. Willemse & J. Tilmans; 1♀ (RMNH): Greece – Crete (Chania): 1 km NE of Anidhroi/385 m; 21.VI.2017; 35.255925°N, 23.737376°E/leg. L. Willemse & P. Zacharopoulou; 6♂ & 2♀ (CT): HELLAS, nomos Khania, 300 m/3 km E. Anidhroi, 27–29.IV.2001/35°15.288'N, 23°44.157'E/leg. J.M. Tilmans & J.F.R. Tilmans-Smid; 1♂ (CT), 2♂ & 1♀ (RMNH): Greece – Crete (Chania): 1 km N of Chondros/485 m; 16.VI.2019; 35.322094°N, 23.685799°E/leg L. Willemse & J. Tilmans; 1♂ & 1♀ (RMNH): Greece – Crete (Chania): Elos/480 m; 16.VI.2019; 35.367374°N, 23.637676°E)/leg. L. Willemse & J. Tilmans; 1♀ (CT), 1♀ (RMNH): Greece – Crete (Chania): 0.5 km W of Kamaria/345 m; 18.VI.2019; 35.282516°N, 23.778568°E/leg. L. Willemse & J. Tilmans; 1♀ (RMNH): 1 km S of Livadas/225 m; 18.VI.2019; 35.263004°N, 23.814722°E/leg. L. Willemse & J. Tilmans; 1♂ (IBER): Louchio, 0.5 km (35.3691°N, 23.6244°E) 665 m, 23/05/2018 Chobanov, D., Iorgu, I. & Borissov, S. 1♂ IBER; 1♂ 2♀ (RMNH): Greece – Crete (Chania); Marouliana, Ano Sfinari – Kostogiannides/715 m; 19.VI.2017; 35.390335°N, 23.605317°E/leg. L. Willemse & P. Zacharopoulou; 2♂ & 2♀ (CT), 3♂ & 4♀ (RMNH): Greece – Crete (Chania): Psariana-Aligi/420 m; 17.VI.2019; 35.351833°N, 23.694208°E/leg. L. Willemse & J. Tilmans; 1♂ & 1♀ (CT), 2♂ & 1♀ (RMNH): Greece – Crete (Chania): 1 km S of Sarakina/305 m; 16.VI.2019; 35.289181°N, 23.674417°E/leg. L. Willemse & J. Tilmans; 2♀ (IBER): Sfinari (35.4407°N, 23.5704°E) 1m, 23/05/2018 Chobanov, D., Iorgu, I. & Borissov, S.; 1♂ (CT), 1♂ & 1♀ (RMNH): Greece – Crete (Chania): just N of Strovles/420 m; 16.VI.2019; 35.368656°N, 23.669718°E/leg. L. Willemse & J. Tilmans; 1♂ (RMNH): Greece – Crete (Chania): 0.5 km N of Temenia/835 m; 17.VI.2019; 35.299652°N, 23.751684°E/leg. L. Willemse & J. Tilmans; 1♂ 1♀ (CT), 1♂ 2♀ (NHMC), 1♀ (RMNH): Greece – Crete (Rethimno): 1 km SE of Piso Moni Preveli/20 m; 12.VII.1997; 35.1518°N, 24.4725°E/leg. P. Lymberakis. (for details see Suppl. material 2).

##### Description.

**Male.** General appearance (Figs 228, 229), elytra and legs as type species of genus, *E.chabrieri*.

Pronotum (Fig. 31) dorsally slightly flattened.

Forewing: stridulatory file left elytron consists of 96–138 teeth, shortest distance between proximal and distal end 3.0–3.9 mm, density of teeth in middle two thirds of the file 27–34 teeth per mm.

Anal tergite (Figs 75, 89, 103) apically strongly curved downward with round dorsomedian depression; posterior margin with wide, concave, moderately deep rounded (in many specimens semi-circular), median excision, bordered by two sharply toothed processes laterally, directed downward.

Cerci (Figs 117, 132) long, slender, 6–7× longer than greatest width, cylindrical with golden-coloured short and long hairs, without any tooth, slightly bent inwards.

Subgenital plate (Figs 146, 160) very large, longer than wide, strikingly elongated, lateral margins swollen, ventrally with a median keel; basal third wide, then suddenly (strongly) incurved to the median part with in many specimens nearly parallel lateral margins; in apical third strongly tapering, hind margin distinctly medially excised over the whole length of the apical third, apical lobes laterally flattened, the apex round spatulate with a well-defined, slightly upwards-pointing, curved tooth at the lower end; in profile pointing backward. Styli (Fig. 174) short, thick, 1.1–1.6× longer than wide, downwardly directed in lateral view, inserted quite far before apex of apical lobe.

Titillator (Figs 189, 205) moderately sized; basal parts extending, strongly curved in the direction of the apical arms; fused part of apical arms broad at base not widening to the beginning of the unfused part of the apical arms; unfused part of apical arms hook-like, parallel or diverging and in lateral view in a 35–50 degrees angle curved upward to dorsum, wide at basis and evenly narrowing to tip; fused part of apical arms as long to longer than unfused part.

**Colouration (in living specimens)**: general colouration in Andikithiran specimens dark brown (in several specimens chestnut brown) (Fig. 243), in Chania specimens green to light brown (Fig. 245). Head: frontal part below antennae and eyes in Andikithiran specimens creamy yellow-brownish with two larger inner and two smaller outer dark brown spots (Fig. 17) and often brownish speckled below the eyes, in Chania specimens bright green and likewise arranged and sized spots in black; border of frons with (lighter coloured) clypeus with dark transverse patches; upper part around eyes and antennal sockets black; behind both eyes and antennae two black bands separated from each other by a yellowish median line; occiput with black marking often provided with a thin lighter median line. Pronotum: dorsum dark brown-chestnut brown (Andikithira) to greenish or yellowish brown and often mottled (Chania) in first half with more (Andikithira) (Fig. 31) or less (Chania) extensive black marking; lateral lobes in upper part with black, ventrally not sharply delimited, longitudinal band, lower part pronotal lobes brownish to pinkish (Andikithira), green or yellow-white (Chania); in many specimens lower margin pronotal lobes in metazona yellowish. Elytra: visible parts not covered by the pronotum black or dark brown, covered part (lighter) brownish. Abdomen: first tergite dorsally black, other tergites completely dark brown to chestnut brown (Andikithira) or green to brownish often dorsally lighter coloured (Chania) and on both islands abdominal tergites sometimes mottled, anal tergite completely black; abdominal sternites pinkish brown (Andikithira) or yellowish brown (Chania). Cercus and subgenital plate: same (general) colour as body. Titillator: basal parts and unfused part of apical arms same colour as body, fused part of apical arms lighter coloured. Legs: same colour as body; fore and middle legs with many blackish to brownish stripes, spots, and markings; hind femur in the basal half dorsally with a longitudinal black to brownish stripe and also laterally on the outside in the middle part of its length; hind knees black.

**Female.** General appearance (Figs 230, 231) as in male. Elytra completely covered by pronotum, only in some females scarcely protruding laterally.

Cercus short, conical with golden coloured short and long hairs, nearly straight, tapering apically; tip pointed, slightly bent inwards.

Subgenital plate (Figs 45, 59) in ventral view generally wider than long; hind margin rounded, medially with a broadly rounded wide V-shaped excision half as long as the subgenital plate; basis concave with a shallow medial longitudinal ridge; in profile short triangular, apex rounded and not reaching or surpassing the proximal half of the gonangulum (Fig. 67).

Ovipositor nearly straight, only slightly upcurved near its apex, 1.5 to almost 2.0× longer than pronotum.

Colouration generally as in male (Figs 244, 246). Black marking of pronotum dorsally in prozona in most females less extensive as in males. First abdominal segment black; cercus, subgenital plate and ovipositor same colour as body (Andikithira) or yellowish brown with tip of ovipositor darker brown and laterally its medial part greyish brown.

Morphological variation found in *E.francisae* sp. nov. is elaborated in the Discussion.

##### Measurements.

See Tables 6, 7.

##### Bioacoustics.

Based upon the sound recordings of 15 specimens (153 syllables), the song of *E.francisae* sp. nov., as in all species of *Eupholidoptera*, consists of isolated syllables produced in long series with the opening hemisyllable much shorter and weaker than the closing hemisyllable. In *E.francisae* sp. nov., the syllable duration is ~ 188 ms (Fig. 218). In the present recordings, the syllable repetition rate is slower than 0,5/s. The song may most likely be confused with the other species of *Eupholidoptera* in Crete, except *E.smyrnensis* and *E.forcipata*. For details of sound recordings of *Eupholidopterafrancisae* sp. nov. see Suppl. material 3.

##### Variation.

**Figures 216, 217.**
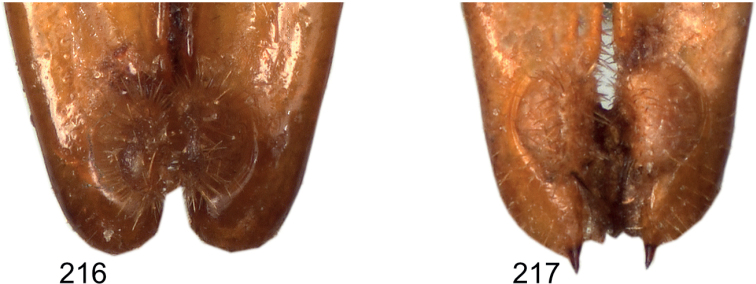
Tip of male subgenital plate in ventral view **216***Eupholidopterafrancisae* sp. nov. paratype Anidhroi CT2001.002.01 **217***Eupholidopterafrancisae* sp. nov. paratype Anidhroi CT2001.002.0.

Within this new taxon, specimens from Andikithira are, as stated earlier, quite uniform in their morphological traits and colouration, while the populations on Crete incorporate more variation as the morphometric analyses in Tables 3, 4 show. For the males this is especially the case in the presence/absence of tiny spines at the tip of the subgenital plate (compare Figs 216, 217), the ratio length-width hind femur, the ratio length-width subgenital plate, the length of the incision of the subgenital plate. The females show most variation in the ratio length-width hind femur. Looking at the differences between the populations of Andikithira and those of western/southwestern Crete, the males and females of Andikithira in general have a larger body length and pronotum length; the males of Andikithira also possess a subgenital plate that is longer and wider than in those from Chania; the females of Andikithira have a subgenital plate that generally is wider than in those from Chania. Moreover, in females from Chania the length of the median incision of the hind margin of the subgenital plate is longer.

**Table 3.** Measurements (in mm) and biometrics of male *E.latens* and *E.francisae*.

Males	*Euph. latens*	*Euph. francisae*	*Euph. francisae* only Andikithira	*Euph. francisae* only Chania
length body	*n* = 9	*n* = 38	*n* = 11	*n* = 27
min. – max.	18.9–27.1	19.0–28.8	20.9–28.3	19.0–28.8
mean ± SD	22.4 ± 2.58	24.1 ± 2.66	25.6 ± 2.58	23.5 ± 2.50
length pronotum	*n* = 9	*n* = 38	*n* = 11	*n* = 27
min. – max.	8.4–9.9	8.0–10.7	9.0–10.7	8.0–10.4
mean ± SD	9.1 ± 0.53	9.5 ± 0.65	10.0 ± 0.53	9.3 ± 0.59
length hind femur	*n* = 13	*n* = 37	*n* = 10	*n* = 27
min. – max.	17.0–22.7	19.0–23.1	19.0–22.8	19.7–23.1
mean ± SD	19.3 ± 2.28	21.1 ± 0.93	20.7 ± 1.10	21.2 ± 0.83
width hind femur	*n* = 13	*n* = 37	*n* = 10	*n* = 27
min. – max.	3.9–4.8	3.7–4.9	4.0–4.6	3.7–4.9
mean ± SD	4.3 ± 0.23	4.3 ± 0.25	4.4 ± 0.18	4.3± 0.27
ratio length-width hind femur	*n* = 13	*n* = 37	*n* = 10	*n* = 27
min. – max.	4.14–5.28	4.47–5.49	4.52–5.07	4.47–5.49
mean ± SD	4.53 ± 0.37	4.88 ± 0.25	4.74 ± 0.16	4.93 ± 0.26
length subg. plate	*n* = 12	*n* = 34	*n* = 11	*n* = 23
min. – max.	3.75–6.30	4.25–5.90	5.35–5.90	4.25–5.80
mean ± SD	4.45 ± 0.74	5.08 ± 0.49	5.57 ± 0.17	4.85 ± 0.41
width subg. plate	*n* = 12	*n* = 34	*n* = 11	*n* = 23
min. – max.	2.20–5.00	2.00–3.85	2.95–3.85	2.00–3.75
mean ± SD	3.21 ± 0.78	3.13 ± 0.48	3.43 ± 0.32	2.99 ± 0.48
ratio length-width subg. plate	*n* = 12	*n* = 34	*n* = 11	*n* = 23
min. – max.	0.84–2.05	1.13–2.44	1.48–1.90	1.13–2.44
mean ± SD	1.44 ± 0.32	1.66 ± 0.30	1.63 ± 0.15	1.67 ± 0.35
length incision subg. plate	*n* = 12	*n* = 32	*n* = 11	*n* = 21
min. – max.	1.15–1.60	1.20–1.90	1.50–1.85	1.20–1.90
mean ± SD	1.33 ± 0.15	1.53 ± 0.21	1.67 ± 0.13	1.46 ± 0.21

**Table 4.** Measurements (in mm) and biometrics of female *E.latens* and *E.francisae*.

**Females**	***Euph. latens***	***Euph. francisae***	***Euph. francisae* only Andikithira**	***Euph. francisae* only Chania**
length body	*n* = 8	*n* = 38	*n* = 11	*n* = 27
min. – max.	17.5–23.3	19.5–31.5	23.7–31.5	19.5–26.9
mean ± SD	20.3 ± 2.32	24.1 ± 2.43	26.3 ± 1.93	23.2 ±1.98
length pronotum	*n* = 8	*n* = 38	*n* = 11	*n* = 27
min. – max.	8.1–9.4	8.5–10.5	8.5–10.5	8.6–10.4
mean ± SD	8.7 ± 0.46	9.6 ± 0.56	9.8 ± 0.65	9.5 ± 0.49
length ovipositor	*n* = 8	*n* = 38	*n* = 11	*n* = 27
min. – max.	13.8–16.4	13.8–19.3	15.8–18.7	13.8–19.3
mean ± SD	14.9 ± 0.76	16.6 ± 1.38	17.5 ± 0.89	16.2 ± 1.39
ratio length ovip. pronot.	*n* = 8	*n* = 38	*n* = 11	*n* = 27
min. – max.	1.64–1.79	1.52–1.99	1.61–1.99	1.52–1.91
mean ± SD	1.72 ± 0.05	1.73 ± 0.11	1.79 ± 0.12	1.71 ± 0.11
length hind femur	*n* = 9	*n* = 37	*n* = 11	*n* = 26
min. – max.	17.1–22.9	20.9–24.9	20.0–21.7	20.9–24.9
mean ± SD	18.8 ± 1.93	22.2 ± 1.04	21.2 ± 0.61	22.6 ± 0.90
width hind femur	*n* = 9	*n* = 37	*n* = 11	*n* = 26
min. – max.	3.9–4.8	4.1–5.1	4.1–4.7	4.1–5.1
mean ± SD	4.3 ± 0.27	4.5 ± 0.24	4.5 ± 0.20	4.5 ± 0.25
ratio length-width hind femur	*n* = 9	*n* = 37	*n* = 11	*n* = 26
min. – max.	3.98–4.77	4.55–5.62	4.55–5.02	4.60–5.62
mean ± SD	4.40 ± 0.25	4.93 ± 0.28	4.76 ± 0.17	5.00 ± 0.28
length subg. plate	*n* = 9	*n* = 35	*n* = 11	*n* = 24
min. – max.	2.10–3.75	1.75–2.70	1.90–2.60	1.75–2.70
mean ± SD	2.68 ± 0.51	2.19 ± 0.23	2.18 ± 0.25	2.19 ± 0.23
width subg. plate	*n* = 9	*n* = 35	*n* = 11	*n* = 24
min. – max.	2.10–3.20	1.95–3.40	2.20–3.40	1.95–2.90
mean ± SD	2.62 ± 0.39	2.53 ± 0.41	2.93 ± 0.39	2.34 ± 0.27
ratio length-width subg. plate	*n* = 9	*n* = 35	*n* = 11	*n* = 24
min. – max.	0.86–1.44	0.59–1.23	0.59–1.00	0.75–1.23
mean ± SD	1.03 ± 0.17	0.89 ± 0.15	0.76 ± 0.14	0.95 ± 0.12
length incision subg. plate	*n* = 9	*n* = 35	*n* = 11	*n* = 24
min. – max.	1.15–1.70	0.90–1.60	0.90–1.25	0.95–1.60
mean ± SD	1.32 ± 0.18	1.16 ± 0.16	1.08 ± 0.12	1.20 ± 0.17

##### Differential diagnosis.

The new species differs from all the other species of the genus by the shape of the strikingly elongated male subgenital plate. Within the *E.prasina* group (male cerci of most taxa possess no tooth) the new species belongs to the *E.latens* subgroup as its preapically situated short styli are downward directed in lateral view. *Eupholidopterafrancisae* sp. nov. seems most related to *E.latens* by the shape and proportions of the male subgenital plate with the apical lobes provided with a tooth at its tip, the proportions of the stylus, the shape of the titillator and the ratio height-length of the hind femur (see Tables 6, 7 for measurements). A phylogeny based on molecular data also clearly separates *E.francisae* from *E.latens* (see discussion).

The male subgenital plate of *E.francisae* sp. nov. (Figs 146, 160) is larger and more elongated than in *E.latens* (Figs 144, 158). The stylus of *E.francisae* sp. nov. is 1.5× longer than wide, while in *E.latens* it is 2–3× longer than wide. The fused parts of the apical arms of the titillator of *E.francisae* sp. nov. (Fig. 189) are broad at base, not widening to the beginning of the unfused part, while in *E.latens* (Figs 186, 187) they are narrow at base and clearly widening to the beginning of the unfused part. The unfused part of the apical arms of the titillator of *E.francisae* sp. nov. is not spine-like, straight and only slightly to moderately curved upward to the dorsum. In contrast, in *E.latens* the unfused part is spine-like and in most specimens strongly hooked upward to the dorsum.

The females of *E.francisae* sp. nov. differ from the other taxa in the genus by the shape and proportions of the subgenital plate (Figs 45, 59). It can be distinguished from females of *E.latens* by the fact that in ventral view the incision of the hind margin is shaped in the form of a wide V, instead of slit-like or narrowly V-shaped as in *E.latens*; in profile the apex of the female subgenital plate of *E.francisae* sp. nov. does not reach or surpass the proximal half of the gonangulum, while in *E.latens* the apex reaches the distal half of the gonangulum or even surpasses it. For more details differentiating *E.francisae* sp. nov. from other Cretan *Eupholidoptera*, see Table 5.

**Figures 218, 219.**
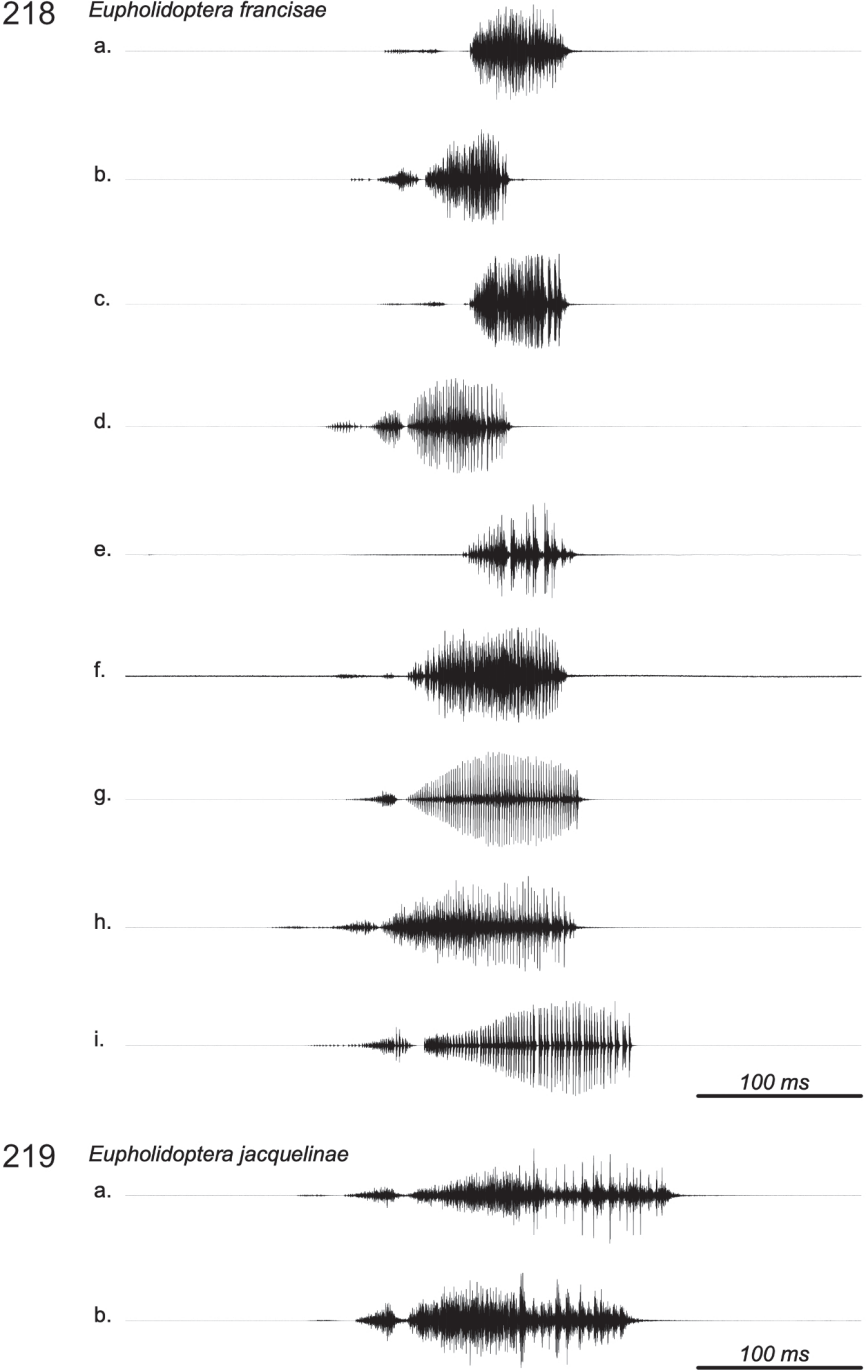
Oscillograms of *Eupholidoptera***218** Single syllables of nine specimens of *E.francisae* sp. nov., timescale 500 ms and temperature 25–27.7 °C **a** 2002.004.10 **b** 2002.004.09 **c** 2002.004.08 **d** 2002.004.07 **e** 2002.004.04 **f** 2002.004.07 **g**RMNH.5087052 **h** 2001.002.02 **i**RMNH.5106281 **219** single syllables of one specimen of *E.jacquelinae*, timescale 500 ms and temperature 23.9–24.5 °C **a, b** 2001.004.12.

**Table 5.** Diagnostic characters and character-states for *Eupholidoptera* species from Crete, Andikithira, Gavdos, and Gavdopoula.

Structure	1			2			3		4				5		6				7			
Character	a	b	c	a	b	c	a	b	a	b	c	d	a	b	a	b	c	d	a	b	c	d
Species																						
*annamariae*	1	3	1	2	2	2	2	1	1	2	1	1	1	1	2	1	1	1	1–3	2	1	1
*astyla*	1	3	1	1	1	1	3	1	1	1	1	1	1	1	3	1	3	1	3–4	2	1	1
*cretica*	1	1	1	1	3	3	2	1	1	2	1	1	3	2	1	1	2	2	1	3	2	1
*feri*	1	3	1	1	1	1	2	3	1	1	1	1	1	1	2	1	2	1	4	2	1	2
*forcipata*	1	1	1	2	3	1	1	1	1–2	3	1	1	2	2	1	1	3	1	1	1	2	3
*francisae*	1	2	1	1	2	1	3	1	3	2	1	1	1	1	1	1	3	2	1–3	3	2	2–3
*gemellata*	3	3	3	1	1	2	2	3	1–3	2	2	2	2	2	1	2	2	2	1	1	2	1
*giuliae*	1	2	1	1	2	1	3	1	1–3	1	1	1	1	3	1	1	3	2	2–4	3	1	3
*jacquelinae*	1	1	1	2	2	1	3	1	3	3	2	1	3	2	1	1	3	2	3–4	3	1	1
*latens*	1	2	1	1	2	1	3	1	(1)-3	2	1	1	2	1	1	1	3	2	2	3	1	2–3
*mariannae*	1	3	1	1	1	3	1	3	2	1	1	1	1	1	2	1	3	1	3–4	2	1	2–3
*marietheresae*	3	1	1	2	3	1	1	1	1	3	1	1	2	2	1	1	3	1	1	2	2	3
*pallipes*	3	3	3	1	1	2	2	3	2	2	2	2	2	2	1	2	1	2	2	1	2	1
*smyrnensis*	2	1	2	1	3	1	1	2	2	3	3	2	2	2	1	1	3	2	3	2	2	1
1. Male and Female colouration																						
a. frons						1. dots (Fig. 12); 2. mosaic (Fig. 11); 3. black patch (Fig. 13)																
b. pronotal disc						1. pale (Fig. 28); 2. small patch (Fig. 29); 3. large patch (Fig. 32)																
c. abdomen						1. pale (Fig. 10); 2. black dot (Fig. 8); 3. black edge (Fig. 9)																
2. Male anal tergite																						
a. extended backwards						1. not/hardly; 2. distinctly																
b. excision tips						1. narrow; 2. intermediate; 3. wide																
c. direction tips						1. downward; 2. inward; 3. forward																
3. Male cercus																						
a. length-width ratio						1. < 5; 2. 5–6.5; 3. > 6.5																
b. side tooth						1. missing; 2. basal; 3. sub-basal																
4. Male subgenital plate																						
a. length-width ratio						1. < 1; 2. ca. 1; 3. > 1																
b. length excision-total length ratio						1. 0.1–0.25; 2. 0.3–0.4; 3. 0.5–0.6																
c. spine						1. absent; 2. one; 3. two																
d. protuberance						1. absent; 2. present																
5. Male styli																						
a. length-width ratio						1. 1.0–2.0; 2.0–3.0; 3. > 4.0																
b. direction						1. downward; 2. backward; 3. inward																
6. Male titillator																						
a. symmetry						1. symmetrical; 2. subsymmetrical; 3. asymmetrical																
b. basal arms						1. long; 2. short																
c. apical arms						1. merged; 2. largely merged; 3. free for > ⅓																
d. apical arms						1. basal half stalk-like; 2. basal half wide																
7. Female subgenital plate																						
a. length-width ratio						1. < 0.75; 2. 0.75–0.90; 3. 0.90–1.10; 4. >1.10																
b. length excision-total length ratio						1. < 0.25; 2. 0.25–0.33; 3. > 0.33																
c. medial excision						1. narrow; 2. wide																
d. proximally						1. convex; 2. concave; 3. with 2 concavities																

**Table 6.** Measurements (in mm), ratios, and biometrics of male *Eupholidoptera*.

Males	Body length	Pronotum length	Hind femur length	Hind femur width	Ratio length-width hind femur	Number of teeth
*annamariae*	*n* = 23	*n* = 23	*n* = 23	*n* = 23	*n* = 23	*n* = 1
mean ± SD	28.3±2.1	11.3±0.5	21.3±0.8	4.8±0.2	4.43±0.16	
min. – max.	25.0–32.2	10.5–13.1	19.6–22.8	4.4–5.1	4.16–4.81	109
*astyla*	*n* = 79	*n* = 79	*n* = 70	*n* = 70	*n* = 70	*n* = 2
mean ± SD	25.5±2.4	9.9±1.0	19.5±2.0	4.5±0.4	4.34±0.17	
min. – max.	21.0–30.8	8.2–12.2	16.2–23.0	3.9–5.3	3.77–4.74	101–105
*cretica*	*n* = 1	*n* = 1	*n* = 1	*n* = 1	*n* = 1	*n* = 1
mean ± SD	n.a.	n.a.	n.a.	n.a.	n.a.	
min. – max.	21.7	8.8	22.1	4.4	5.04	107
*feri*	*n* = 1	*n* = 1	*n* = 1	*n* = 1	*n* = 1	*n* = 1
mean ± SD	n.a.	n.a.	n.a.	n.a.	n.a.	
min. – max.	26.8	8.8	18.6	4.0	4.61	100
*forcipata*	*n* = 20	*n* = 20	*n* = 20	*n* = 20	*n* = 20	*n* = 1
mean ± SD	23.4±1.6	9.5±0.5	17.8±0.7	4.3±0.2	4.18±0.11	
min. – max.	20.8–26.7	8.6–10.5	16.6–19.6	4.0–4.7	3.96–4.43	193
*francisae*	*n* = 38	*n* = 38	*n* = 37	*n* = 37	*n* = 37	*n* = 19
mean ± SD	24.1±2.7	9.5±0.7	21.1±0.9	4.3±0.3	4.88±0.25	119±10.3
min. – max.	19.0–28.8	8.0–10.7	19.0–23.1	3.7–4.9	4.47–5.49	96–138
*gemellata*	*n* = 3	*n* = 4	*n* = 4	*n* = 4	*n* = 4	*n* = 1
mean ± SD	21.1±3.9	7.4±0.3	17.1±0.2	4.0±0.1	4.32±0.10	
min. – max.	16.8–24.2	7.0–7.7	16.8–17.3	3.9–4.1	4.17–4.45	101
*giuliae*	*n* = 52	*n* = 52	*n* = 52	*n* = 52	*n* = 52	*n* = 1
mean ± SD	24.5±3.2	10.2±0.5	21.7±0.8	4.7±0.2	4.66±0.24	
min. – max.	17.2–30.2	9.1–11.4	20.0–23.8	4.0–5.0	4.26–5.43	106
*jacquelinae*	*n* = 6	*n* = 7	*n* = 7	*n* = 7	*n* = 7	*n* = 1
mean ± SD	27.4±2.5	10.6±0.3	24.8±1.1	4.8±0.2	5.14±0.36	
min. – max.	25.3–32.1	10.2–11.2	23.8–26.4	4.4–5.1	4.86–5.98	144
*latens*	*n* = 9	*n* = 9	*n* = 13	*n* = 13	*n* = 13	*n* = 4
mean ± SD	22.4±2.6	9.1±0.5	19.3±2.3	4.3±0.2	4.53±0.37	113±7.1
min. – max.	18.9–27,1	8.4–9.9	17.0–22.7	3.9–4.8	4.14–5.28	108–123
*mariannae*	*n* = 9	*n* = 9	*n* = 9	*n* = 9	*n* = 9	*n* = 4
mean ± SD	24.4±2.2	10.1±0.4	21.2±0.9	4.6±0.3	4.65±0.17	
min. – max.	21.5–28.9	9.5–10.9	19.8–22.3	4.2–4.9	4.36–5.00	89–105
*marietheresae*	*n* = 5	*n* = 5	*n* = 5	*n* = 5	*n* = 5	*n* = 2
mean ± SD	24.9±2.7	9.4±0.6	17.1±0.6	4.0±0.2	4.30±0.14	
min. – max.	21.4–28.5	8.9–10.3	16.4–18.1	3.8–4.2	4.04–4.45	211–213
*pallipes*	*n* = 6	*n* = 6	*n* = 6	*n* = 6	*n* = 6	*n* = 1
mean ± SD	20.3±1.0	7.7±0.3	16.9±0.6	4.1±0.1	4.18±0.13	
min. – max.	19.3–21.9	7.4–8.1	16.5–18.0	3.9–4.2	3.92–4.32	94
*smyrnensis*	*n* = 7	*n* = 7	*n* = 7	*n* = 7	*n* = 7	*n* = 1
mean ± SD	20.2±1.6	9.8±0.4	23.3±0.6	4.5±0.1	5.24±0.19	
min. – max.	17.5–22.6	9.5–10.3	22.4–24.0	4.3–4.6	4.92–5.48	100

**Table 7.** Measurements (in mm), ratios, and biometrics of female *Eupholidoptera*.

Females	Body length	Pronotum length	Hind femur length	Hind femur width	Ratio length-width hind femur	Ovipositor length	Ratio length ovipositor-length pronotum
*annamariae*	*n* = 24	*n* = 24	*n* = 24	*n* = 24	*n* = 24	*n* = 24	*n* = 24
mean ± SD	25.6±2.6	10.8±0.5	21.7±0.6	4.9±0.2	4.48±0.14	19.0±1.1	1.76±0.09
min. – max.	21.7–32.2	10.2–12.1	20.4–22.8	4.6–5.2	4.20–4.81	17.0–21.2	1.63–2.05
*astyla*	*n* = 59	*n* = 59	*n* = 59	*n* = 59	*n* = 59	*n* = 59	*n* = 59
mean ± SD	23.8±2.8	9.6±1.1	20.1±2.1	4.5±0.4	4.41±0.19	17.3±1.9	1.80±0.13
min. – max.	17.8–30.7	7.9–11.9	16.8–23.8	3.8–5.2	3.77–4.89	14.0–20.9	1.42–2.16
*cretica*	*n* = 2	*n* = 2	*n* = 2	*n* = 2	*n* = 2	*n* = 2	*n* = 2
mean ± SD	n.a.	n.a.	n.a.	n.a.	n.a.	n.a.	n.a.
min. – max.	22.2–24.2	9.1–9.6	21.4–22.4	4.6–4.9	4.36–4.87	18.1–18.2	1.86–1.99
*feri*	*n* = 1	*n* = 1	*n* = 1	*n* = 1	*n* = 1	*n* = 1	*n* = 1
mean ± SD	n.a.	n.a.	n.a.	n.a.	n.a.	n.a.	n.a.
min. – max.	27.8	9.6	21.7	4.9	4.43	16.1	1.67
*forcipata*	*n* = 14	*n* = 14	*n* = 14	*n* = 14	*n* = 14	*n* = 14	*n* = 14
mean ± SD	24.2±2.7	9.5±0.8	19.0±1.9	4.4±0.4	4.38±0.27	17.9±0.8	1.90±0.14
min. – max.	21.6–30.8	8.3–11.00	16.6–22.8	4.0–5.3	3.96–5.04	16.7–19.6	1.63–2.12
*francisae*	*n* = 38	*n* = 38	*n* = 37	*n* = 37	*n* = 37	*n* = 38	*n* = 38
mean ± SD	24.1±2.4	9.6±0.6	22.2±1.0	4.5±0.2	4.93±0.28	16.6±1.4	1.73±0.11
min. – max.	19.5–31.5	8.5–10.5	20.9–24.9	4.1–5.1	4.55–5.62	13.8–19.3	1.52–1.99
*gemellata*	*n* = 2	*n* = 2	*n* = 2	*n* = 2	*n* = 2	*n* = 2	*n* = 2
mean ± SD	n.a.	n.a.	n.a.	n.a.	n.a.	n.a.	n.a.
min. – max.	20.5–22.1	7.4–10.0	16.5–22.4	4.2–4.6	3.93–4.92	13.8–14.2	1.42–1.86
*giuliae*	*n* = 30	*n* = 30	*n* = 30	*n* = 30	*n* = 30	*n* = 30	*n* = 30
mean ± SD	24.2±2.7	10.3±0.4	22.3±0.9	4.8±0.2	4.65±0.19	17.7±1.5	1.73±0.15
min. – max.	18.7–29.8	9.4–10.9	21.1–24.2	4.5–5.4	4.38–5.13	15.7–21.2	1.45–2.12
*jacquelinae*	*n* = 7	*n* = 7	*n* = 7	*n* = 7	*n* = 7	*n* = 7	*n* = 7
mean ± SD	28.3±2.9	10.6±0.4	26.2±0.8	5.3±0.1	4.96±0.16	20.3±0.9	1.91±0.09
min. – max.	24.9–33.6	10.2–11.1	25.2–27.5	5.1–5.4	4.74–5.29	19.4–21.8	1.76–2.04
*latens*	*n* = 8	*n* = 8	*n* = 9	*n* = 9	*n* = 9	*n* = 8	*n* = 8
mean ± SD	20.3±2.3	98.7±0.5	18.8±1.9	4.3±0.3	4.40±0.25	14.9±0.8	1.72±0.05
min. – max.	17.5–23.3	8.1–9.4	17.1–22.9	3.9–4.8	3.98–4.77	13.8–16.4	1.64–1.79
*mariannae*	*n* = 15	*n* = 15	*n* = 14	*n* = 14	*n* = 14	*n* = 15	*n* = 15
mean ± SD	22.4±2.8	8.9±1.0	19.0±2.4	4.3±0.3	4.43±0.27	15.9±1.2	1.80±0.22
min. – max.	19.1–27.5	7.5–10.3	16.5–22.8	3.9–4.9	3.92–4.74	13.6–17.5	1.41–2.23
*marietheresae*	*n* = 6	*n* = 6	*n* = 6	*n* = 6	*n* = 6	*n* = 6	*n* = 6
mean ± SD	23.6±3.3	8.3±0.7	18.5±2.1	4.2±0.2	4.36±0.39	17.9±1.1	2.15±0.14
min. – max.	20.5–28.8	7.4–9.5	16.6–22.6	3.9–4.4	3.95–5.16	15.9–18.9	1.98–2.38
*pallipes*	*n* = 1	*n* = 1	*n* = 1	*n* = 1	*n* = 1	*n* = 1	*n* = 1
mean ± SD	n.a.	n.a.	n.a.	n.a.	n.a.	n.a.	n.a.
min. – max.	21.7	9.5	23.8	4.6	5.23	13.5	1.43
*smyrnensis*	*n* = 5	*n* = 5	*n* = 5	*n* = 5	*n* = 5	*n* = 5	*n* = 5
mean ± SD	20.0±1.8	9.9±0.3	23.6±0.9	4.6±0.1	5.18±0.18	17.6±0.8	1.78±0.12
min. – max.	17.5–22.6	9.5–10.3	22.4–24.7	4.4–4.7	4.92–5.48	16.6–18.6	1.61–1.96

##### Distribution.

This new taxon has been found on the island of Andikithira situated some 32 km NW of Crete and also in the western and southwestern part of Chania in western Crete (Fig. 254). Andikithira is a geographically isolated island halfway between the island of Kithira in the northwest and Crete in the southeast. It is a small, dry, and stony island (20.43 km^2^) rising to not more than 378 meters above sea level. The island has only few tens of residents and is hardly visited by tourists.

In Chania populations of *E.francisae* sp. nov. have been encountered west and southwest from the line of Gramvousa peninsula (northwest coast) to Livadas (near the south coast and situated 3–4 km west of the famous Samaria Gorge). Worth mentioning is also the fact that several males and females of *E.francisae* sp. nov. were caught in 1997 in a pitfall trap near Piso Moni Preveli. This location a long way to the east along the southern coast of the Rethimno region is ~ 60 km (in a straight line) east of Livadas. Piso Moni Preveli is also situated near the eastern (sic!) boundary of *E.giuliae*. Why *E.francisae* sp. nov. occurs here and has not been found on intermediate locations is puzzling. A revisit to this location to confirm its presence is necessary to rule out a mistake of mislabelling. For a complete list of localities, specimens and repositories see Suppl. material 1.

##### Habitat.

On Andikithira the species was found in phrygana and garrigue that cover a significant part of the entire island. Most specimens were collected as nymphs in *Sarcopoteriumspinosum* that is present all over the island. The collecting sites are situated 50–150 m above sea level. But this species probably is present from sea level to the highest points of the island wherever phrygana and garrigue formations are present. On one of the collecting sites on Andikithira the new species was found together with the first specimen of *Rhacocleisandikithirensis* (Tilmans et al. 2016). On both collecting sites on Andikithira also *Poecilimoncretensis* or a new taxon closely linked to it (pers. obs.) was present. In southwestern Chania *E.francisae* sp. nov. was not only found in low prickly shrublets in phrygana, but also frequently on tall shrubs of blackberry (*Rubus*).

##### Etymology.

Named in honour of Mrs. Francis Smid-Elbers, the late mother-in-law of the second author. Together with her husband Jacques Smid, she enthusiastically collected many interesting Orthoptera specimens in Greece, also from Crete. For instance, the paratype male and female of *E.giuliae* from 2.5 km E. of Argoules.

##### Phenology.

On Andikithira most specimens were collected as nymphs becoming adult in the period 22 May–10 June. In Chania collected nymphs became adult in the period 26 May–6 June and adults were collected in the period 23 May–21 June. Adults of *Eupholidopterafrancisae* sp. nov. can thus be encountered from the end of May throughout June to July and possibly even later.

#### ﻿ Eupholidoptera gemellata

Taxon classificationAnimaliaOrthopteraTettigoniidae

Willemse & Kruseman, 1976

4BC7A85B-39D7-57DA-8364-5F41F59E53A4

Figs 12 , 26 , 40 , 54 , 70 , 84 , 98 , 112 , 127 , 141 , 155 , 169 , 183 , 199 , 232 , 233 , 255 , Tables 1 , 2 , 5 , 6 , 7 , 9 , 10 , Suppl. materials 1 , 2

Eupholidoptera gemellata Willemse & Kruseman, 1976: 136.
Morphological description. Willemse and Kruseman 1976: 137.

##### Remark.

The species was described after a single male was collected in 1973. Pitfall catches made in 2000–2001 at Mt. Psiloritis at 1950 m above Lochria and Agia Marina caught 11 males and 8 females. Opportunity is taken here to describe the female and illustrate important morphological structures with stacked images.

##### Examined specimens.

**Holotype**, 3 ♂, 2 ♀ (for details see Suppl. material 2).

##### Diagnostic features.

Frontal part of head (Fig. 12) pale with two larger and two smaller dark dots; pronotal disc (Fig. 26) pale with irregular small or large black patches or largely blackish, posterior quarter to third pale; narrow band along anterior margin fourth to ninth abdominal tergites black. Male – stridulatory file with 101 teeth (including proximal and distal ones), density of teeth in middle two thirds of the file 22 teeth per mm; anal tergite (Figs 70, 84, 98) wide, distally bend downward centrally forming two inward pointing, overlapping, spines separated by short circular excision; cerci (Figs 112, 127) 4–5× longer than wide, conical, straight in profile and dorsal view, armed with inward curved inner sub-basal rectangular sidetooth; subgenital plate (Figs 141, 155) ca. as wide as long, proximally widest, sides rimmed except in apical quarter, in profile, narrowing, straight, pointing backward, tip apical lobes widely truncate with a protuberance on the inner margin and strong upward and backward pointing curved spine at base of stylus, with V-shaped excision along one third of total length; styli (Fig. 169) long, more than half as long as cerci, 3× longer than wide, conical, inserted at tip of apical lobe, pointing backwards; titillator (Figs 183, 199) symmetrical, weakly sclerotised, basal arms short, apical arms fused, in apical quart widened and split, tip truncate, unarmed in profile S-shaped from base to tip equally wide.

##### Description.

**Female.** Examined specimens. 2 ♀: RETHIMNO: Psiloritis, above Lochria, FC1602 1♀ RMNH.INS1141844 (RMNH) 1♀ 2000.095.02 (CT). For more details, see Suppl. material 2.

General appearance and size as male (Figs 232, 233). Colouration as male. In dorsal view wings covered by pronotum, in profile hardly visible, light coloured. Cercus short, conical, slightly more than half as long as subgenital plate, straight in profile and in dorsal view, conical, tapering in apical third toward a pointed tip, densely covered with pale short and long hairs. Subgenital plate (Figs 50, 54) wider than long, in profile triangular, in ventral view trapezoid, basally widest, hind margin medially with wide shallow excision; surface basally and centrally convex, laterally flattened, thinly covered with hairs; ovipositor proximal two thirds straight, apical third slightly curved upward, 1.4–1.9× longer than pronotum.

##### Measurements.

See Tables 6, 7.

##### Bioacoustics.

The song of this species has not yet been recorded.

##### Differential diagnosis.

Males differ from congenerics in the stout, straight cercus (Figs 112, 127) with sub-basal rectangular side-tooth, in the subgenital plate (Figs 141, 155) narrowing into a truncate tip with upward and backward pointing spines, the inner margin of the excision with a protuberance, in the long, apically inserted styli (Fig. 169) pointing backward, in the anal tergite (Figs 70, 84, 98), medially bent downward forming a small circular excision adjoined by two partly overlapping, inward pointing spines and in the titillator (Figs 183, 199) with short basal arms and fused and adjoined apical arms with widened and truncated tip. Females differ in the wide, convexly rounded subgenital plate (Figs 50, 54), hind margin centrally with wide shallow excision. *Eupholidopteragemellata* closely resembles *E.pallipes* but males differ in the apical arms of the titillator apically not being fused in *E.gemellata* (Fig. 183) and fused with small lateral spinelets in *E.pallipes* (Fig. 184). Females of both species differ in the shape and the hind margin of the subgenital plate (compare Fig. 40 with Fig. 41). In colouration *E.gemellata* is easily recognisable by the head with larger frontal black dots, the extensive blackening of the pronotal disc and narrow anterior transverse black band in the abdominal tergites. For more details differentiating *E.gemellata* from other Cretan *Eupholidoptera* see Table 5.

##### Distribution.

The holotype was collected on Mt. Idi at 1650 m near the spring of Skaronero. Additional specimens collected in pitfall traps at a site northwest of Skaronero at 1950 m above Lochria between 15 September 2000 and 12 June 2001 (Fig. 255). For a complete list of localities, specimens, and repositories see Suppl. material 1.

##### Habitat.

Rocky mountain slopes with phrygana.

##### Phenology.

The holotype was collected at 1650 m on 28 July. The pitfalls that trapped the species were positioned at 1910 m and emptied on 15 September and 30 October 2000, and again on 12 June 2001.

#### ﻿ Eupholidoptera giuliae

Taxon classificationAnimaliaOrthopteraTettigoniidae

Massa, 1999

EF64048A-9EA1-5F42-A17D-55AA260C3466

Figs 16 , 30 , 44 , 58 , 74 , 88 , 102 , 116 , 131 , 145 , 159 , 173 , 188 , 204 , 220–223 , 247 , 248 , 254 , 256 , 257 , 258 , 259 , Tables 1 , 2 , 5 , 6 , 7 , 8 , 9 , 10 , Suppl. material 1 , 2 , 3 , 4

Eupholidoptera giuliae Massa, 1999: 72.
Morphological description. Massa 1999: 72–75; Willemse and Heller 2001: figs 8, 16, 23, 30, 45.

##### Examined specimens.

2 ♂, 1♀ (**paratypes**); 52 ♂, 30 ♀ (for details see Suppl. material 2).

##### Diagnostic features.

Frontal part of head (Fig. 16) pale with black dots; frontal half of pronotal disc (Fig. 30) with extensive central black patch not reaching sides, border with pale rear half diffuse or distinct V-shaped. Male (Fig. 247) – stridulatory file with 106 teeth (including proximal and distal ones), density of teeth in middle two thirds of the file 23 teeth per mm; anal tergite (Figs 74, 88, 102) wide, centrally depressed, distally bend downward forming two pointed lobes pointing downward and slightly outward separated by wide excision; cerci (Figs 116, 131) unarmed, 5–6× longer than wide, basal half cylindrical, apical half conical almost straight to slightly curved inward, in profile straight; subgenital plate (Figs 145, 159) wider than long, widest in proximal third, sides rimmed in proximal half, in profile upturned, pointing upward, tip apical lobes rounded, spineless, at inner side emarginate with V-shaped excision along one seventh of length; styli (Fig. 173) minute, square to circular, as long as wide, inserted at internal margin of apical lobes proximal of tip, pointing inward to slightly downward; titillator (Figs 188, 204) symmetrical, apical arms proximally fused, halfway to two thirds diverging into two parallel or divergent, smooth hooks, in profile basal half distinctly wider than in ventral or dorsal view, halfway recurved, forming wide angle with beak-like evenly, weakly curved spines. Female (Fig. 248) – subgenital plate (Figs 44, 58) generally as long as wide, widest in proximal third, proximally with two distinct, dark-coloured concavities separated by a median ridge, tip apical lobes acute, rounded, separated by slit-like medial excision along one third to half the length, in profile triangular, ventrally and in proximal upper corner depressed.

##### Measurements.

See Tables 6, 7.

##### Bioacoustics.

Based upon the sound recordings of 5 specimens (50 syllables measured), the song of *E.giuliae* – as in all species of *Eupholidoptera* – consists of isolated syllables produced in long series with the opening hemisyllable much shorter and weaker than the closing hemisyllable. In *E.giuliae*, the syllable duration is ~ 199 ms, with a syllable rate up to ~ 1/s. There are no published descriptions of the song of this species. The song may most likely be confused with the other species of *Eupholidoptera* in Crete, except *E.smyrnensis* and *E.forcipata*. For details of sound recordings of *Eupholidopteragiuliae* see Suppl. material 3.

##### Variation.

**Figures 220–223.**
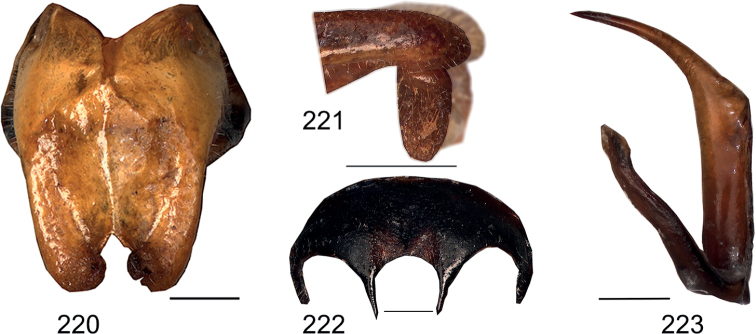
Male terminalia *Eupholidopteragiuliae* complex **220** subgenital plate in ventral view Drapanos RMNH.5086980 **221** styli in lateral view Skloka CT2019.022.01 **222** anal tergite in caudal view Skloka CT2019.022.01 **223** titillator in lateral view Skloka CT2019.022.01 Scale bars: 1 mm.

Along the south coast and more to the northeast up toward the town of Rethimno males show little variation in cerci, anal tergite, subgenital plate or titillator. Styli are small, mostly pointing inward but in some specimens somewhat downward. In the titillator the two apical arms are mostly divergent but sometimes almost parallel and close to each other. It is unclear whether such variation is structural, the result of the drying up process after killing or the age of the specimen in number of days after the final moult. Toward the northwest, in the municipalities of Chania and Apokoronas, male subgenital plates (Fig. 220) are longer than wide with long styli pointing downward (Fig. 221) resembling the subgenital plate in *E.latens*. A unique feature found in males from this area are the teeth bordering the medial excision in the hind margin of the anal tergite (Fig. 222) which are distinctly longer than in other areas. Notwithstanding differences in the subgenital plate and anal tergite, populations from Chania and Apokoronas have been assigned to *E.giuliae* because the titillator with its slender apical arms and long apical hooks (Fig. 223) perfectly fits this species. The morphological variation in *E.giuliae*, its geographical pattern and links to *E.latens*, are further elaborated in the discussion.

##### Differential diagnosis.

Males differ from congenerics in the wide, upturned, spineless subgenital plate (Figs 145, 159) with styli (Fig. 173) inserted at the inner margin of apical lobes pointing inward to slightly downward, in the anal tergite (Figs 74, 88, 102) medially bent downward forming two widely separated lobes with tips pointing downward and slightly outward, in the slender, weakly inward bent, unarmed cerci (Figs 116, 131) and in the stout apical arms of the titillator (Figs 188, 204), fused in basal half, separated into two strong, long, parallel to diverging curved hooks. Females differ in the elongated subgenital plate (Figs 44, 58), proximally with two distinct concavities, apical lobes with slit-like excision along one third to half the length. In colouration, the amount of black shown by *E.giuliae* is intermediate between overall pale coloured species such as *E.cretica*, *E.jacquelinae* and *E.smyrnensis* and dark coloured species like *E.annamariae*, *E.astyla*, or *E.mariannae*. For more details differentiating *E.giuliae* from other Cretan *Eupholidoptera*, see Table 5.

##### Distribution.

The species was described from Chora Sfakion and a site 2.5 km east of Argoules along the southwestern coast of Crete (Massa 1999; Çiplak et al. 2009). Additional data gathered over the past years indicate that *E.giuliae* occurs from the eastern part of the Chania regional unit to the western part of the Rethimno regional unit not only along the southern coast but across the island up to the northern coast (Fig. 254). The westernmost find of *E.giuliae* just south of Chania town is quite close to the easternmost find of *E.latens* from Lakki. For a complete list of localities, specimens and repositories see Suppl. material 1.

##### Habitat.

Based on current data *E.giuliae* is a lowland species occurring from sea level up to some 500 m. It has been found in a variety of habitats ranging from very dry phrygana covered hills and *Quercus* forest, to rather wet lush vegetations including ferns.

##### Phenology.

Adults of this species have been collected by hand from 10 May to 24 June. This is also the period during which most pitfall catches were made but at least in one instance *E.giuliae* adults were caught in a trap that had been set 20 August. This indicates that although adults may appear early in the season and may be most numerous in June, they can still be found until late August.

#### ﻿ Eupholidoptera jacquelinae

Taxon classificationAnimaliaOrthopteraTettigoniidae

Tilmans, 2002

80F3E45D-4C76-5C3E-800E-87BD737480EA

Figs 24 , 38 , 52 , 66 , 82 , 96 , 110 , 125 , 139 , 153 , 167 , 181 , 197 , 212 , 219 , 242 , 254 , 256 , 259 , Tables 1 , 2 , 5 , 6 , 7 , 10 , Suppl. material 1 , 2 , 3 , 4

Eupholidoptera jacquelinae Tilmans, 2002: 157.
Morphological description. Tilmans 2002: 157.

##### Examined specimens.

**Holotype, allotype**, 2 ♂ (**paratypes**); 4 ♂, 6 ♀ (for details see Suppl. material 2).

##### Diagnostics features.

Frontal part of head (Fig. 24) pale with black dots. Pronotal dorsum (Fig. 38) brown to yellow, orange-brown flush with dark brown markings in middle of prozona, metazona yellow with brown flush, pronotal lateral lobes with black dorsal fascia, in prozona not sharply delimited ventrally and strongly narrowing posteriorly, rest of pronotal lateral lobes yellow with vague brown marks except for broad bright yellow margin in metazona; elytra area near the fore margin white, other parts black; anal tergite black, other tergites in living specimens brownish to olive-green; subgenital plate from cream white to bright yellow. Male – stridulatory file left elytron (paratype 2001.004.12): length 4.4 mm, width 0.1 mm, total number of teeth (including proximal and distal ones) 144, density of teeth in middle two thirds of the file 29 teeth per mm; cercus (Figs 125, 139) slender, 8–9× longer than wide, without any tooth, slightly bent inwards; subgenital plate (Figs 153, 167) in ventral view convex, remarkably slender with well-defined median keel and next to it on both sides a depression; strongly elongated apical lobes that (faintly discernible) pass into long styli that form an extension of the lobes; apex of the apical lobes dorsally armed with a sharp spine that covers the basis of the stylus (Fig. 181); anal tergite (Figs 82, 96, 110) in dorsal view with a round dorsomedian depression, in caudal view posterior margin triangularly extended ventrally with V-shaped medial excision, strongly curved frontally, provided with an apical tooth on either side, surface of processes with transverse wrinkles, depression and processes densely covered with golden-coloured hairs; titillator (Figs 197, 212) small, with basal parts extending, weakly curved laterally, apical parts fused, slightly swollen in basal half with medial depression, divided in apical half, from the narrow basis widening up to middle of basis half, from there narrowing apically, tips simply pointed, parallel, surface with transverse wrinkles, in lateral view moderately curved dorsally. Female (Fig. 242) – subgenital plate (Figs 52, 66) varying from longer than wide to somewhat wider than long, slightly impressed on both sides of median groove, in some females with transverse faint wrinkles, hind margin obliquely convergent toward a triangular, median excision along one third of total length, apical lobes diverging with narrowly posterior angles, lateral sides slightly impressed.

##### Measurements.

See Tables 6, 7.

##### Bioacoustics.

Based upon the sound recordings of one specimen, the song of *E.jacquelinae*, as in all species of *Eupholidoptera*, consists of isolated syllables produced in long series with the opening hemisyllable much shorter and weaker than the closing hemisyllable. In *E.jacquelinae*, the syllable duration is ~ 231 ms (Fig. 219). In the present two recordings, the syllable repetition rate is slower than 1/s. Although the recordings do not permit detailed analysis, it seems that the first part of the closing hemisyllable contains a more densely series of teeth impacts and the second part a more loosely series. This would suggest the closing movement initially is fast but ends slowly. The song may most likely be confused with the other species of *Eupholidoptera* in Crete, except *E.smyrnensis* and *E.forcipata*. For details of sound recordings of *Eupholidopterajacquelinae* see Suppl. material 3.

**Figures 224–235.**
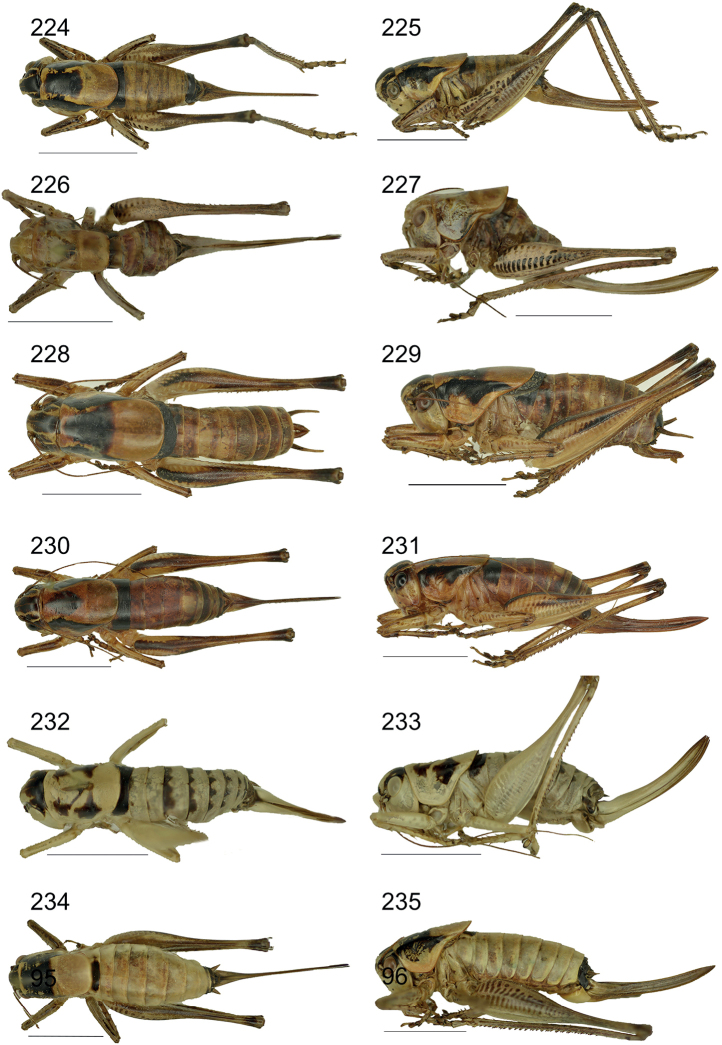
Habitus *Eupholidoptera* spp. in dorsal and lateral view **224, 225***Eupholidopteraastyla* ♀ Mt. Idhi CT1987.046.03 **226, 227***Eupholidopteracretica* ♀ Mt. Lefka Omalos FC17807 RMNH.INS1141837 **228, 229***Eupholidopterafrancisae* sp. nov. ♂ holotype Andikithira CT2002.004.04 **230, 231***Eupholidopterafrancisae* sp. nov. ♀ allotype Andikithira CT2002.004.11 **232, 233***Eupholidopteragemellata* ♀ Mt. Idhi FC1651 RMNH.INS1141844 **234, 235***Eupholidopteramariannae* ♀ Ag. Ioannis RMNH5014906 1844. Scale bars: 10 mm.

**Figures 236–239.**
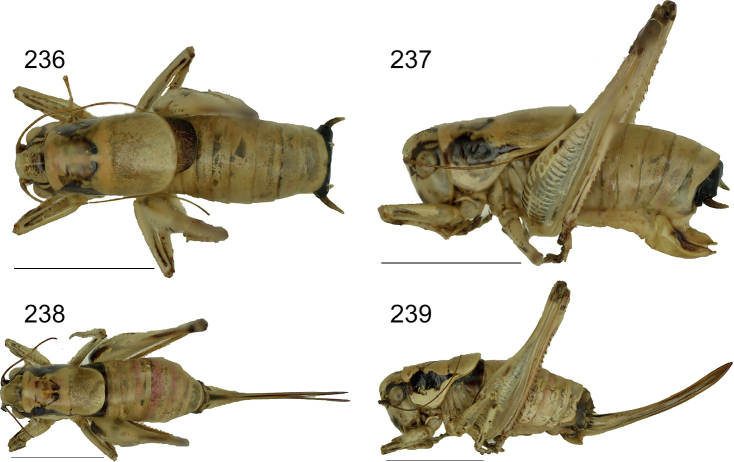
Habitus *Eupholidopteramarietheresae* sp. nov. in dorsal and lateral view **236, 237** ♂ holotype Mt. Dikti FC1606 RMNH.INS1141850 **238, 239** ♀ allotype Mt. Dikti FC1606 RMNH.INS1141849. Scale bars: 10 mm.

##### Variation.

For the description of *E.jacquelinae* in 2002 only three males and one female from Gavdos were available. At present more specimens are at hand, also from the islet of Gavdopoula. We compared seven males (5 from Gavdos and 2 from Gavdopoula) and seven females (also 5 from Gavdos and 2 from Gavdopoula). The males, compared with the holotype, show no differences in colour, marking, last abdominal tergite, subgenital plate with styli, cercus, or titillator (from three males). The females, however, compared to the allotype, show some variation in marking and the form of the subgenital plate. Most of the females show more black markings on the lateral lobes of the pronotum than the allotype, but even then, less markings than in the males. A bit more than half of the females studied possesses a subgenital plate that is not longer than wide as in the allotype, but as long as wide or even a little bit wider than long. In one female from Gavdos and one of Gavdopoula the median groove of the subgenital plate fades away toward the basis. In a small number of the females the subgenital plate show faint transverse wrinkles.

##### Differential diagnosis.

Male *E.jacquelinae* is differentiated from all other (Cretan) *Eupholidoptera* by its uniquely shaped, strongly elongated apical lobes of the subgenital plate (Figs 153, 167) armed with a long apical spine that dorsally covers the basis of the long styli that form an extension of the lobes. Females differ in the subgenital plate (Figs 52, 66), that is a bit longer than wide to a little wider than long combined with a medial incision of its hind margin measuring more than one third of the length of the subgenital plate. In colouration *E.jacquelinae* is one of the Cretan *Eupholidoptera* species with no or only minute black marking on the pronotal disc. For more details differentiating *E.jacquelinae* from other Cretan *Eupholidoptera* see Table 5.

**Figures 240–246.**
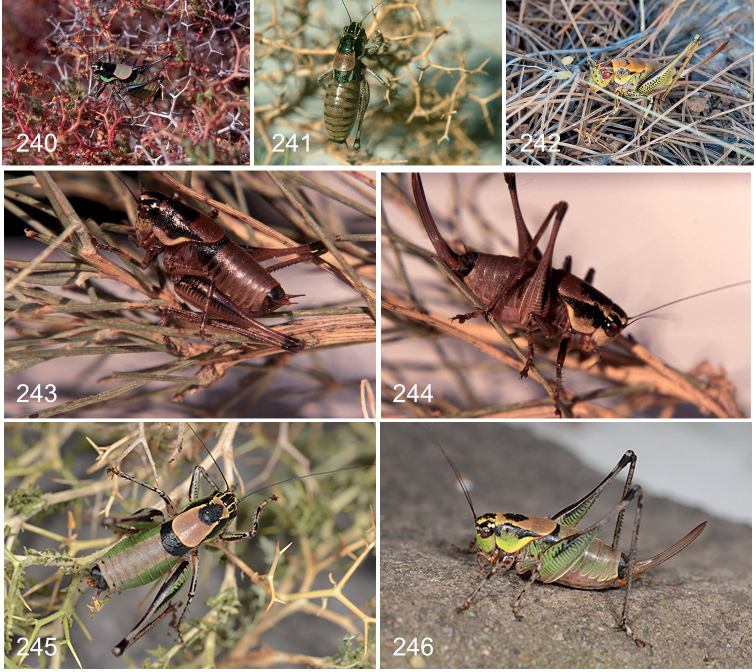
Field images *Eupholidoptera* spp. Crete **240***Eupholidopteraannamariae* ♂ Kata Zakros CT1995.0530 **241***Eupholidopteraastyla* ♂ Mt. Idhi CT2004.08.14 **242***Eupholidopterajacquelinae* ♀ Gavdos **243***Eupholidopterafrancisae* sp. nov. ♂ paratype Andikithira CT2002.004.07 **244***Eupholidopterafrancisae* sp. nov. ♀ paratype Andikithira CT2002.004.12 **245***Eupholidopterafrancisae* ♂ paratype Marouliana RMNH5087052 **246***Eupholidopterafrancisae* ♀ paratype Marouliana RMNH5014917.

##### Distribution.

Restricted to the islets of Gavdos and Gavdopoula, south of western Crete (Fig. 254). For a complete list of localities, specimens and repositories see Suppl. material 1.

##### Habitat.

The habitats where the species was found consists of rocky ground with sparse vegetation of low trees (*Pinusbrutia*), thorny shrubs and smaller plants as well as sand dunes with *Pistacia*, *Juniperus*, and *Tamarix*. The male specimens of *E.jacquelinae* were collected by hand on bushes of *Ericamanipuliflora*, *Pistacia*, *Tamarix*, and *Pinus* but not on prickly bushes like *Juniperus* or *Sarcopoteriumspinosum*, a spiny shrublet very common on Crete but much less so on Gavdos. The females however were found hiding under the low spiny shrubs of *Euphorbiaacanthothamnos*. Trap catches on Gavdos and Gavdopoula recorded the species between 8 and 270 m.

**Figures 247–253.**
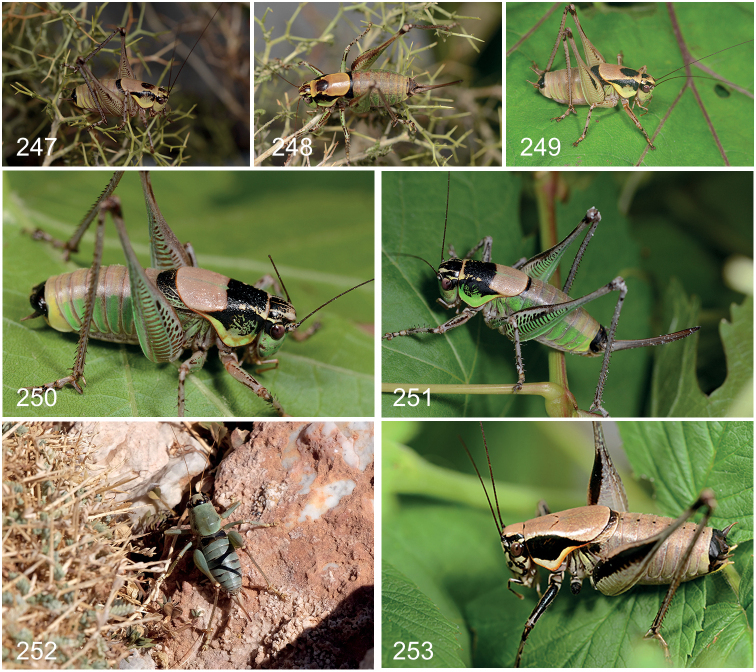
Field images *Eupholidoptera* spp. Crete **247***Eupholidopteragiuliae* ♂ Prasies RMNH5087051 **248***Eupholidopteragiuliae* ♀ Rethymnon RMNH5014921 **249***Eupholidopteralatens* ♂ Kolympari RMNH553681 **250***Eupholidopteramariannae* ♂ Kalavros RMNH5014907 **251***Eupholidopteramariannae* ♀ Ag. Ioannis RMNH5014906 **252***Eupholidopterapallipes* ♀ below Pakhnes **253***Eupholidopterasmyrnensis* ♂ Makrigiannis RMNH5087053.

##### Phenology.

The holotype male together with a paratype male was collected on 11 June at 50 m. The allotype female together with another paratype male was collected as nymph in the period 30 April to 2 May, becoming adult 25 May. Additional females were collected on 5 August. Specimens were found in traps emptied between mid-March and mid-November.

#### ﻿ Eupholidoptera latens

Taxon classificationAnimaliaOrthopteraTettigoniidae

Willemse & Kruseman, 1976

1E2F5C7D-3A98-52D2-86E7-ED67E87F7BEB

Figs 15 , 29 , 43 , 57 , 68 , 73 , 87 , 101 , 115 , 130 , 144 , 158 , 172 , 186 , 187 , 202 , 203 , 249 , 254 , 256 , 257 , 258 , 259 , Tables 1 , 2 , 3 , 4 , 5 , 6 , 7 , 8 , 9 , 10 , Suppl. materials 1 , 2 , 3 , 4

Eupholidoptera latens Willemse & Kruseman, 1976: 134.
Morphological description. Willemse and Kruseman 1976: 134, 135.
Bioacoustics. Çiplak et al. 2009: 27, 51, 54, 55.

##### Examined specimens.

**Holotype, allotype**, 4 ♂, 3f (**paratypes**); 12 ♂, 7 ♀ (for details see Suppl. material 2).

##### Diagnostics features.

Frons (Fig. 15) pale with black dots; pronotal disc (Fig. 29) rarely completely pale, mostly frontal half with more or less well defined black central patch, border with pale rear half. V-shaped. Male (Fig. 249) – stridulatory file with 108–123 teeth (including proximal and distal ones), density of teeth in middle two thirds of the file 26–31 teeth per mm; anal tergite (Figs 73, 87, 101) centrally depressed, distally bend downward, forming two lobes with teeth like apex pointing downward, separated by wide excision; cerci (Figs 115, 130) unarmed, 6–7× longer than wide, in basal half conical, in apical half cylindrical, slightly curved inward in basal half, in profile straight to weakly upturned; subgenital plate (Figs 144, 158) longer than wide, widest in proximal third, tapering and distinctly narrower in distal two thirds, sides rimmed in proximal third, in profile pointing backward, tip apical lobes rounded, spineless, with narrow median excision along one quarter of length its proximal half narrower; styli (Fig. 172) short, one quarter as long as cerci, 2–3× longer than wide, round or flattened, inserted at ventral side of apical lobes, proximal of tip, pointing downward; titillator (Figs 186, 187, 202, 203) symmetrical, apical arms fused, parallel to slightly widening toward apical third inflated, diverging into two straight diverging hook-like teeth, in profile basal two thirds parallel, in second third swollen forming wide angle with two weakly upward curved hook-like teeth. Female – subgenital plate (Figs 43, 57) generally slightly wider than long, proximally with two concavities separated by keel, tip apical lobes rectangular, rounded, with slit-like to narrow V-shaped excision reaching one third to halfway, in profile rhomboid, upper distal angle rectangularly rounded.

##### Measurements.

See Tables 6, 7.

##### Bioacoustics.

Based upon the sound recordings of nine specimens (49 syllables measured), the song of *E.latens*, as in all species of *Eupholidoptera*, consists of isolated syllables produced in long series with the opening hemisyllable much shorter and weaker than the closing hemisyllable. In *E.latens*, the syllable duration is ~ 228 ms, with a syllable rate up to ~ 1/s. Published records (Çiplak et al. 2009) show a syllable duration of ~ 161 ms and a syllable repetition rate of ~ 0.5/s at maximum. The song may most likely be confused with the other species of *Eupholidoptera* in Crete, except *E.smyrnensis* and *E.forcipata*. For details of sound recordings of *Eupholidopteralatens* see Suppl. material 3.

##### Variation.

*Eupholidopteralatens* is restricted to the northern and central Chania region in western Crete. The cerci and anal tergite show little variation across its range. Titillators in males from the Rodopou peninsula (Figs 187, 203) are relatively slender, with longer apical hooks, being intermediate between *E.latens* and *E.giuliae*. In males, morphological variation is most pronounced in the subgenital plates. In males from Kolympari, Rodhopos, and Kato Kefalia in central and northern Chania, as well as in the outlier found in Rethimno the subgenital plate is clearly more slender than in the populations of the Lefka area, the transition between the wide basal part and the narrow apical part generally being more distinct. The morphological variation found in *E.latens*, *E.giuliae*, and *E.francisae* sp. nov. is further elaborated in the discussion.

##### Differential diagnosis.

Males differ from congenerics in the stout apical arms of the titillator (Figs 186, 187, 202, 203), fused in basal half, separated into two strong, short, parallel or diverging curved hooks in the elongated, in the slender subgenital plate (Figs 144, 158) narrowing at one third of the length, the apical lobes without a teeth at its apex and with short styli (Fig. 172), inserted pre-apically pointing downward, the anal tergite (Figs 73, 87, 101) medially bent downwards forming two widely separated lobes with short pointed tips pointing downward and in the slender, unarmed, weakly inward and upward bent cerci (Figs 115, 130). *Eupholidopteralatens* most closely resembles *E.francisae* sp. nov. but differs in the shape of the subgenital plate (compare Fig. 144 with Fig. 146), the length-width ratio of styli, the shape of the titillator (compare Figs 186, 187 with Fig. 189), body size and the ratio height-length of the hind femur (see Tables 3, 4 for measurements). Females differ in the subgenital plates (Figs 43, 57) being as long as wide, proximally with two concavities, slit-like to narrow V-shaped excision between the apical lobes reaching halfway. They can be distinguished from females of *E.francisae* by the fact that in ventral view the incision of the hind margin in *E.francisae* is shaped in the form of a wide V; in profile the apex of the female subgenital plate in *E.latens* reaches the distal half of the gonangulum or surpasses it (Fig. 68) while in *E.francisae* it does not reach or surpass the proximal half of the gonangulum (Fig. 67). In colouration, the amount of black shown by *E.latens* is intermediate between overall pale coloured species like *E.cretica*, *E.jacquelinae* and *E.smyrnensis* and dark-coloured species like *E.annamariae*, *E.astyla*, or *E.mariannae*. For more details differentiating *E.latens* from other Cretan *Eupholidoptera* see Table 5.

##### Distribution.

**Figures 254, 255.**
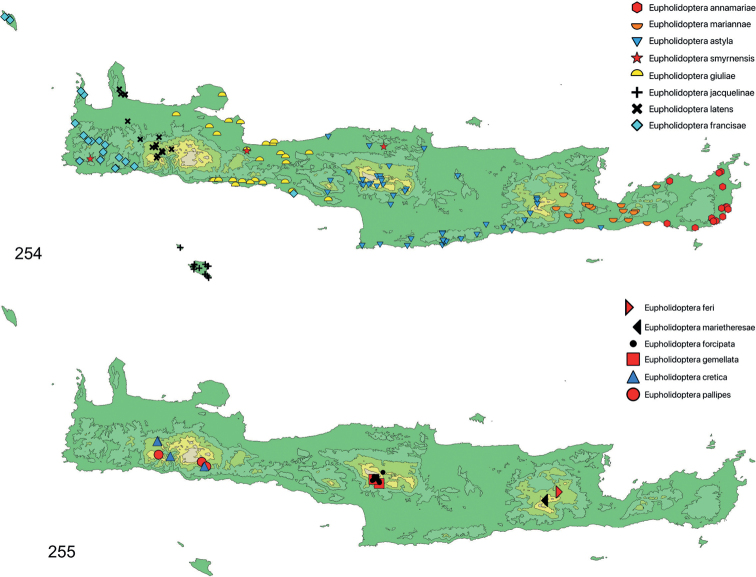
Distribution maps *Eupholidoptera* Crete and adjacent islands **254** lowland species **255** highland species.

The species was discovered in 1973 at high altitudes on Mt. Lefka, western Crete and published records from this species originated only from this mountain and its foothills (Lakki). Specimens collected between 2016 and 2019 indicate the species also occurs at low altitudes in the northern and northeastern parts of Chania region (Fig. 254). To the east the distribution area of *E.latens* borders to but is separated from *E.giuliae*. For a complete list of localities, specimens and repositories see Suppl. material 1.

##### Habitat.

The species occupies habitats from sea level to alpine regions between 1600 and 1800 m on Mt. Lefka. It hides in low prickly shrublets in the phrygana while at lower altitudes it was also found on shrubs of blackberry (*Rubus*) or gorse (*Ulex*).

##### Phenology.

At low altitudes adults appear around mid-May, at mid-level elevations toward the end of May or early June whereas at high altitudes it may take to the second half of July before the first adults appear.

#### ﻿ Eupholidoptera mariannae

Taxon classificationAnimaliaOrthopteraTettigoniidae

Willemse & Heller, 2001

C2D55B1A-55A3-5B54-ACB4-992941DA6D4A

Figs 21 , 35 , 49 , 63 , 79 , 93 , 107 , 122 , 136 , 150 , 164 , 178 , 194 , 209 , 214 , 215 , 234 , 235 , 250 , 251 , 254 , 256 , 259 , Tables 1 , 2 , 5 , 6 , 7 , 10 , Suppl. materials 1 , 2 , 3 , 4

Eupholidoptera mariannae Willemse & Heller, 2001: Willemse et al. 2018: figs 946, 947.
Morphological description. Willemse and Heller 2001: 329–331.
Bioacoustics. Willemse and Heller 2001: 331; Çiplak et al. 2009: 27, 54, 55.

##### Examined specimens.

**Holotype**, 1 ♂ (**paratype**); 8 ♂, 15 ♀ (for details see Suppl. material 2).

##### Diagnostic features.

Frons (Fig. 21) pale with black dots; frontal half of pronotal disc (Fig. 35) predominantly black sharply with transverse rarely a V-shaped border with pale rear half; elytra black, veins and cross-veins more or less extensively yellow. Male (Fig. 250) – stridulatory file with 89–105 teeth (90 in Çiplak et al. 2009) (including proximal and distal ones), density of teeth in middle two thirds of the file 18–23 teeth per mm (18–20 in Çiplak et al. 2009); anal tergite (Figs 79, 93, 107) very wide, bilobed, lobes separated by groove, distally bend downward, centrally forming two teeth pointing forward separated by narrow, densely haired pits; cerci (Figs 122, 136) 3× longer than wide, proximally wide, flattened, strongly narrowing in second third, apical third cylindrical, pointing outward, in profile bent upward, armed with short, strong inner side-tooth at ca. one fifth of length; subgenital plate (Figs 150, 164) as wide as long, widest in proximal third, sides rimmed, in profile pointing upward and backward, tip apical lobes rounded, spineless, with slit-like excision along one fifth of length; styli (Fig. 178) minute, flat or depressed, inserted at ventral side of apical lobes, proximal of tip, pointing downward; titillator (Fig. 194, 209) slightly asymmetrical, apical arms strongly sclerotised, narrow, except for very tip, fused along entire length, smooth, needle shaped tip pointing somewhat laterad, in profile narrow, middle third slightly wider evenly upward curved.

##### Measurements.

See Tables 6, 7.

##### Bioacoustics.

Based upon the sound recordings of two specimens (20 syllables measured), the song of *E.mariannae*, as in all species of *Eupholidoptera*, consists of isolated syllables produced in long series with the opening hemisyllable much shorter and weaker than the closing hemisyllable. In *E.mariannae*, the syllable duration is ~ 172 ms. In the present recordings, the syllable repetition rate is very low. Published records (Çiplak et al. 2009) show a syllable duration of ~ 123 ms and a syllable repetition rate of 1/s at maximum. The song may most likely be confused with the other species of *Eupholidoptera* in Crete, except *E.smyrnensis* and *E.forcipata*. For details of sound recordings of *Eupholidopteramariannae* see Suppl. material 3.

##### Variation.

Apical arms of the titillator in most specimens are fused, in some they become apically somewhat separated. The central pits in the anal tergite may be more or less well developed and more or less densely haired.

##### Description of female.

Examined specimens. 15 ♀: LASITHI: Agios Ioannis – RMNH.5014906 (RMNH); Anatoli – s.n. [paratype of *E.astyla*Ramme 1927] (MfNB); Kalavros -2017.029.02 (CT) RMNH.5014912, RMNH. 5086974 (RMNH); Katharo plain, 1 km NE FC17787 – RMNH.INS1124470, RMNH.INS1124471 (RMNH); Kato Chorion s.n. [paratype of *E.astyla*Ramme 1927] (MfNB); Kavousi – 1999.029.02 (CT), RMNH.INS1141830 (RMNH); Koutsouras – 2002.007.05 (CT); Prina, 0.5 km N FC17798 – 2019.061.02 (CT), RMNH.INS1141839 (RMNH); Mt. Thrypti – 2019.032.01 (CT), FC25104 RMNH.INS1124469 (RMNH). (For details see Suppl. material 2).

General appearance and colouration as male (Figs 234, 235, 251). First abdominal segment dorsally darkened, in one female black; second segment may also be somewhat darkened. Fore wings covered by pronotum, in profile barely protruding, pale coloured.

Cercus short, conical hardly tapering but for apical third which is distinctly narrower, tip pointed, slightly upturned in profile, straight in dorsal view, covered with pale short and long hairs.

Subgenital plate (Figs 49, 63) longer than wide, mitre-shaped, in profile triangular; hind margin toward middle forming two distinct pointed apical lobes, separated by a deep and wide excision along one third of length; dorsal margin usually at least partly visible in the apical half as a protruding edge or bulge, in profile the apical half straight, the basal half straight except for the proximal part which is concave; ventral side proximally with two dark coloured concavities separated by a more or less distinct keel, the apical half flattened to shallowly depressed with a median keel, surface smooth without wrinkles, with dispersed hairs.

Ovipositor almost straight, apically slightly upcurved, 1.4–2.2× longer than pronotum.

##### Differential diagnosis.

Males differ from congenerics in the stout, upturned, proximally flattened cercus, pointing outward (Figs 122, 136), sub-basally armed with short strong inner tooth, in the anal tergite (Figs 79, 93, 107) medially not extended, bent downward with very narrow V-shaped excision, tips pointing forward, in the wide, upturned, spineless subgenital plate (Figs 150, 164) with minute, pre-apically inserted styli (Fig. 178) pointing downward and in the barely asymmetrical, narrow, fused apical arms of the titillator (Figs 194, 209). Females differ from congenerics in the elongated subgenital plate (Figs 49, 63) proximally with two concavities, apical lobes pointed, separated by wide excision, as deep as one third of the length. In colouration, the amount of black shown, *E.mariannae* together with *E.annamariae*, *E.astyla*, *E.feri*, and *E.francisae* sp. nov. belongs to the darkest coloured species. For more details differentiating *E.mariannae* from other Cretan *Eupholidoptera* see Table 5.

##### Distribution.

The species was described from southwestern Lasithi. Recent findings indicate its distribution area also includes western central Lasithi from the eastern slopes of Mt. Dikti eastward up to Kalavros in the north and Koutsouras in the south (Fig. 254). In the east there seems to be no overlap with *E.annamariae* although near Kalavros and Xerokampos both species were found within 5 km of each other. *Eupholidopteramariannae* and *E.astyla* may overlap in the Ierapetra area but up to now co-occurrence could not be confirmed. In the northwest, *E.mariannae* was also found in the Katharo plain where *E.feri* was discovered. For a complete list of localities, specimens and repositories see Suppl. material 1.

##### Habitat.

The altitudinal range of *E.mariannae* is considerable. It ranges from sea level where it was found near Koutsouras, to 1475 m, the highest altitude at which it was found on Mt. Thrypti. The type series was found inside open pine forest and groups of planted olive trees. Near Kalavros the species was found on open hill slopes with quite a dense vegetation of small shrubs interspersed with taller shrubs. Near Kavousi (Pacheia Amos) the species was trapped on a hill covered by phrygana. Around Malles and Anadoli individual males were heard singing on the branches of olive trees a few meters above the ground.

##### Phenology.

At low altitudes adults can be found already in early May, at higher altitudes starting from 300–700 m up to 1475 m, adults appear in June or July and have been caught until the end of September and mid-October.

#### ﻿ Eupholidoptera marietheresae

Taxon classificationAnimaliaOrthopteraTettigoniidae

Willemse & Kotitsa sp. nov.

B0D22523-596D-5E0A-A925-7B21817156FF

https://zoobank.org/8119FA55-0B62-4158-8323-C64A5E307A9F

Figs 23 , 37 , 51 , 65 , 81 , 95 , 109 , 124 , 138 , 152 , 166 , 180 , 196 , 211 , 236–239 , 255 , 256 , Tables 2 , 5 , 6 , 7 , 9 , 10 , Suppl. materials 1 , 2 , 4

##### Remark.

Pitfall catches collected on Mt. Dikti at a site above the Limnakaro plateau trapped a total of 127 specimens of *Eupholidoptera* which at first glance were identified as *E.forcipata*. Closer examination however revealed differences with *E.forcipata* based on which a species new to science is described here.

##### Examined specimens.

**Type locality**. Greece, Crete, Lasithi, Dikti Mt., above Limnakaro plateau, SE of Ag. Anastasi mountain refuge, SW Spathi Madharas, 35.1107°N, 25.4779°E, 1715 m

##### Type specimens.

**Holotype** ♂, RMNH.INS1141850, **allotype** ♀, RMNH.INS1141849. Both pinned (from alcohol) with original label: “Dikti Mt., above Limnakaro plateau, SE of Ag. Anastasi mountain refuge, SW Spathi Madharas. FC1606”; FC1606 operated between 05/08/2000–02/10/2000 (RMNH)

**Paratypes.** 1 ♂ RMNH.INS11418546 (pinned from alcohol), 1 ♂ RMNH.INS1124467 (alcohol), 3 ♀ RMNH.INS1141847, RMNH.INS1141848, RMNH.INS1141849 (pinned from alcohol), 1 ♀ RMNH.INS1124468 (alcohol) (RMNH); 2 ♂ 2000.096.01, 2000.096.03 2 ♀ 2000.096.02, 2000.096.04 (pinned from alcohol (CT), same location and date as holotype (for details see Suppl. material 2).

##### Additional specimens

**(not examined).** 40 ♂, 37 ♀ in alcohol (NHMC) LASITHI: Dikti Mt., above Limnakaro plateau, SE of Ag. Anastasi mountain refuge, SW Spathi Madharas, 35.1107°N, 25.4779°E, 1715 m, FC1606, 05/08/2000–02/10/2000; 17 ♂, 24 ♀ in alcohol (NHMC) same as holotype, FC1536 12/05/2000–05/08/2000; 2 ♀ in alcohol (NHMC) same as holotype, FC1655 02/10/2000–09/01/2001.

##### Description.

**Male.** General appearance (Figs 236, 237) as type species *E.chabrieri*, more compact. Pronotum hardly widening posteriorly, metazona relatively short, hind margin slightly convex. Legs short and thick, hind femur 1.7–1.9× as long as pronotum (2.0–2.2 in *E.chabrieri*), mid and hind femur ventrally unarmed.

Stridulatory file with 211–216 teeth (including proximal and distal ones), density of teeth in middle two thirds of the file 37–42 teeth per mm.

Anal tergite (Figs 81, 95, 109) in dorsal view narrow in the middle, laterally widening, in the middle folded downward, centrally forming large pale coloured hairy patch in the centre, laterally with striae, hind margin somewhat swollen, from the ventro-lateral corner folded around cerci, extending straight down- and inward, toward the middle forming two wide downward pointing lobes with teethlike tip separated by a wide excision.

Cercus (Figs 124, 138) compact, unarmed, conical, weakly and gradually curved inward in basal half, in profile slightly upturned, 4× as long as the greatest width.

Subgenital plate (Figs 152, 166) very compact, 1.5× wider than long, widest at one third of the length, proximal margin slight concave; in profile compact, lower margin straight ca. halfway bent upward, hardly narrowing apically, apical part pointing upward in situ covering last abdominal segment; ventral surface with a strong median keel, proximal half depressed next to the keel, halfway transversely depressed, apical half divided into two flattened triangular lobes, tip straight truncated, without a spine, surface irregularly gibbose, laterally with straight rod-like protuberances; lateral margin in ventral view a folded back rim, thickened near the base, apically disappearing under the rod-like protuberances, in profile straight with a proximal nod; posterior margin with a very wide V-shaped median excision along more than half the total length, edges straight toward the middle weak convex. Styli (Fig. 180) short, 2× longer than wide, conical, inserted at the tip of the apical lobes pointing backward and upward.

Titillator (Figs 196, 211) symmetrical, apical arms in dorsal/ventral view in lower half narrow, almost stalk-like apically widening, swollen, two apical arms diverging into two very long and slender hooks, smooth except for some wrinkles, near the apex curved inward; in profile stalk like basal half weakly S-curved, transition with apical half distinct, apical hooks evenly curved backward and upward, reaching or extending above the anal tergite.

General colouration (based on specimens kept in alcohol) yellowish brown. Head with the frontal part below antennae and eyes pale with two black dots (Fig. 23), the lower part frons and upper part clypeus with large transverse black patch, upper part around the eyes and antennal sockets black, occiput behind the eyes and in the middle with black patches. Pronotal disc (Fig. 37) pale yellowish with central black markings at best resembling an open “W” or frontal half with large central black patch forming transverse or V-shaped border with pale rear half; lateral lobes with wide black dorsal fascia, not sharply delineated ventrally, posteriorly narrowing not reaching the hind margin. Elytra black. Fore and middle legs with few black dots and stripes, concentrated around the knee. Hind femur dorsally with black basal patch and apical black stripe, outer side near the base with or without a series of transverse stripes, pre- and post-genicular part black. Anal tergite black, subgenital plate laterally below the dorsal margin black.

**Female.** General appearance (Figs 238, 239) as male. Elytra clearly visible in profile. Cercus conical, hairy, bent inward, narrowing in apical fifth, apex pointed. Subgenital plate (Figs 51, 65) shiny, smooth with very few hairs, 1.5× wider than long, greatest width in proximal third; in ventral view, convex with a median ridge, proximally with two distinct, dark coloured concavities, apically flattened, hind margin with a wide acute V-shaped median excision along one third of the length, corners obtuse rectangular; in profile oblong, ventrally with a distinct basal depression and more shallow apical depression, lower edge convex, distally upturned, tip obtuse angular. Ovipositor 2.0–2.4× as long as pronotum, straight, in apical quart weakly upturned. Colouration as the male, transverse black patch at the transition between frons and clypeus more pronounced; elytra pale; first abdominal segment black.

##### Measurements.

See Tables 6, 7.

##### Bioacoustics.

The song of this species has not yet been recorded.

##### Differential diagnosis.

Males differ from congenerics in the pointed back- and downwardly extended widened lobes of the anal tergite (Figs 81, 95, 109) with tips pointing downward and slightly outward, in the wide upturned, spineless subgenital plate (Figs 152, 166) with a very wide V-shaped excision, in the short, apically inserted styli (Fig. 180) pointing backward, in the stout, weakly inward curved cerci (Figs 124, 138) and the symmetrical apical arms of the titillator (Figs 196, 211), in basal half fused and narrow, in apical half strongly diverging hooks. Females differ from congenerics in the very wide subgenital plate (Figs 51, 65), proximally with two concavities, tips rounded with U-shaped excision along quarter of the length. *Eupholidopteramarietheresae* sp. nov. closely resembles *E.forcipata* but differs in the male by the thin, apically incurved apical arms of the titillator, the compact weakly incurved cercus lacking the subtle bulge halfway the inner side and the anal tergite with wider downward expansion combined with a narrower excision and in the female in a more elongated subgenital plate, with the proximal pits being close together. In colouration, *E.marietheresae* sp. nov. differs from all Cretan congenerics except *E.pallipes* in the large transverse black patch on the lower part of the frons. For more details differentiating *E.marietheresae* sp. nov. from other Cretan *Eupholidoptera* see Table 5.

##### Distribution.

Only known from a single location on Mt. Dikti above the Limnakaro plateau where the species was trapped in pitfall traps (Fig. 255). For a complete list of localities, specimens and repositories see Suppl. material 1.

##### Habitat.

Mountain slopes at 1700 m in phrygana vegetation.

##### Phenology.

Pitfall traps in which the species was found were checked irregularly. Based on the three catching periods, adults can be found prior to early August up to at least early October.

##### Etymology.

The species is named in honour of Marie-Therèse Willemse-Dresen (1929–2017) wife and lifelong companion of Fer Willemse who contributed a large part of his entomological career to the study of the Orthoptera fauna of Greece, describing 40 species new to science from Greece, including four Cretan species of *Eupholidoptera*. In his last, and most challenging, paper on Chorthippus (Glyptobothrus) from Greece (Willemse et al. 2009) he wrote in the acknowledgements:

“*This publication would not have been possible without help from my family. My wife’s patience and tolerance to my single-minded enthusiasm was almost boundless. Both the long hours spent during our travels in seemingly dull and uninteresting areas, as well as at home recording and studying have been accepted without much ado. For that I owe her an enormous amount of gratitude.*”

It is in this spirit that we pay a tribute to Marie-Therèse. The fact that *E.marietheresae* sp. nov. is found on the same mountain and in the vicinity of *E.feri*, a species named after Fer Willemse, is making it even more appropriate.

#### ﻿ Eupholidoptera pallipes

Taxon classificationAnimaliaOrthopteraTettigoniidae

Willemse & Kruseman, 1976

C590E8C4-2654-5503-811C-C9719D1E0287

Figs 9 , 13 , 27 , 41 , 55 , 71 , 85 , 99 , 113 , 128 , 142 , 156 , 170 , 184 , 200 , 252 255, Tables 1 , 2 , 5 , 6 , 7 , 10 , Suppl. materials 1 , 2

Eupholidoptera pallipes Willemse & Kruseman, 1976: 135.
Morphological description. Willemse and Kruseman 1976: 135, 136.

##### Examined specimens.

**Holotype, allotype**, 5 ♂ (**paratypes**) (for details see Suppl. material 2).

##### Diagnostic features.

Frontal part of head (Fig. 13) pale with two large black patches joined or not into a transverse band; pronotum (Fig. 27) pale except for small black patch in the rear of the side flap; abdomen pale, proximal margins tergites black. Male – stridulatory file with 94 teeth (including proximal and distal ones), density of teeth in middle two thirds of the file 22 teeth per mm; anal tergite (Figs 71, 85, 99) wide with central groove, distally bend downward, forming two spines pointing downward and inward almost touching each other separated by rectangular excision; cerci (Figs 113, 128) 5× longer than wide, cylindrical, central third conical, weakly curved inward and upward, armed with sharply pointed inward curved sub-basal tooth; subgenital plate (Figs 142, 156) as wide as long, proximally widest, sides rimmed almost up to apex, in profile distally narrowing, straight, pointing backward, tip apical lobes truncate with protuberance on the inner margin and strong upward and backward pointing curved spine at base of stylus, with V-shaped excision along one third of total length; styli (Fig. 170) long, more than half as long as cerci, 3× longer than wide, cylindrical, inserted at tip of apical lobes, pointing backward and outward; titillator (Figs 184, 200) symmetrical, weakly sclerotised, basal arms short, apical arms completely fused, widening in basal third, gradually narrowing in apical two thirds, near tip widening again, tip rounded at either side with a tiny thorn, in profile equally wide, straight, in apical half weakly curved upward. Female (Fig. 252) – subgenital plate (Figs 41, 55) as long as wide, proximally convex, apical lobes rectangularly rounded separated by wide concave median excision along quarter of total length, in profile rhomboid, apically truncate, upper angle widely rounded.

##### Measurements.

See Tables 6, 7.

##### Bioacoustics.

The song of this species has not yet been recorded.

##### Differential diagnosis.

Males differ from congenerics in the stout, straight cerci (Figs 113, 128) with a sub-basal rectangular side-tooth, in the subgenital plate (Figs 142, 156) narrowing into truncate tips with an upward and backward pointing spine, a protuberance on the inner margin of the excision, in the long, apically inserted, backward pointing styli (Fig. 170), in the anal tergite (Figs 71, 85, 99), medially bent downward forming an excision adjoined by two inward and downward pointing spines and in the titillator (Figs 184, 200) with short basal arms and completely fused apical arms, apically widened, tip rounded at either side with a tiny thorn. Females differ from congenerics in the subgenital plate (Figs 41, 55), as long as wide, convex hind margin centrally with wide excision a deep as quarter of the length. *Eupholidopterapallipes* closely resembles *E.gemellata* but males differ in the apical arms of the titillator apically being fused in *E.pallipes* (Figs 184, 200), its tip with a tiny lateral thorn, in *E.gemellata* (Figs 183, 199) being adjoined the tip being bare. Females of both species differ in the shape and the hind margin of the subgenital plate (compare Figs 40, 41). In colouration *E.pallipes* differs from congenerics, except *E.gemellata*, in the general pale colouration in particular of the legs and the narrow anterior transverse black band in the abdominal tergites (Fig. 9). For more details differentiating *E.pallipes* from other Cretan *Eupholidoptera* see Table 5.

##### Distribution.

The type series was collected in 1973 on Mt. Lefka at the saddle of Linoseli above Xyloskalo between 1600 m and 1800 m. Additional specimens were collected in pitfall traps operated in the summer of 1991 more to the east on Mt. Lefka above Limnia (Fig. 255). On 13 October 2017 a female was photographed just below the Pakhnes peak at 2440 m. For a complete list of localities, specimens and repositories see Suppl. material 1.

##### Habitat.

Rocky mountain slopes with phrygana between 1600 and 2440 m.

##### Phenology.

The type series was collected 5 August 1973. Pitfalls above Limnia trapped adults between early August and early September and during the entire month of October whereas a trap operated between early June and early July only contained nymphs.

#### ﻿ Eupholidoptera smyrnensis

Taxon classificationAnimaliaOrthopteraTettigoniidae

(Brunner von Wattenwyl, 1882)

644A8541-49AD-5A73-A962-430469FC7C3B

Figs 8 , 11 , 25 , 39 , 53 , 69 , 83 , 97 , 111 , 126 , 140 , 154 , 168 , 182 , 198 , 253 , 254 , 256 , 259 , Tables 2 , 5 , 6 , 7 , 9 , 10 , Suppl. materials 1 , 2 , 3 , 4

Thamnotrizon smyrnensis Brunner von Wattenwyl, 1882: 336.
Olynthoscelis smyrnensis (Brunner von Wattenwyl, 1882): Bolivar 1899: 601.
Pholidoptera smyrnensis (Brunner von Wattenwyl, 1882): Ebner 1919: 157.
Eupholidoptera smyrnensis (Brunner von Wattenwyl, 1882): Ramme 1951: 198.
Morphological description. Brunner von Wattenwyl 1882: 336; Willemse 1980: 59.
Bioacoustics. Heller 1988: 132; Çiplak et al. 2009: 27, 54.

##### Examined specimens.

9 ♂, 5 ♀ (for details see Suppl. material 2).

##### Diagnostics features.

Frontal part of head (Fig. 11) pale, dark spots enlarged and merged into a mosaic pattern; pronotal disc (Fig. 25) completely pale chestnut brown; abdomen pale, each tergite with tiny central dot on hind margin. Male (Fig. 253) – stridulatory file with 78 teeth (including proximal and distal ones) (Çiplak et al. 2009 report a stridulatory file with 100 teeth), density of teeth in middle two thirds of the file 19 teeth per mm; anal tergite (Figs 69, 83, 97) oblong, distally bend downward forming two strong spines pointing downward, separated by a very wide semi-ellipsoid excision; cerci (Figs 111, 126) 3× longer than wide, basal half conical, apical half cylindrical, curved inward, in profile straight, armed with strong basal inner side-tooth; subgenital plate (Figs 140, 154) ca. as wide as long, halfway widest, sides unrimmed, in profile distally narrowing, straight, pointing backward, tip apical lobes emarginate with protuberance at inner margin and two small upward pointing teeth, with V-shaped median excision along half the total length; styli (Fig. 168) long, one third as long as cerci, 2–3× longer than wide, cylindrical, inserted at ventro-outer tip of apical lobes, pointing distad and outward; titillator (Figs 182, 198) symmetrical, apical arms mostly fused, from narrow base plate-like expanded, apically divided into two long parallel or diverging spines, in profile moderately upcurved. Female – subgenital plate (Figs 39, 53) as long as wide, in ventral view strongly convex, hind margin converging to two pointed apical lobes separated by wide V-shaped excision a third to a quarter as deep as the total length, in profile triangular, upper and lower margin converging to a pointed apex.

##### Measurements.

See Tables 6, 7.

##### Bioacoustics.

Based upon the sound recordings of one specimen (10 syllables measured) from Crete, the song of *E.smyrnensis*, as in all species of *Eupholidoptera*, consists of isolated syllables produced in long series with the opening hemisyllable much shorter and weaker than the closing hemisyllable. In *E.smyrnensis*, the syllable duration is ~ 40 ms, the shortest in Cretan *Eupholidoptera*. In the present recordings, the syllable repetition rate is 2/s at maximum. The song is also characterised by syllables that are produced in compact series of 3–10s followed by a longer silence after which another series follows. The first few syllables in a series are weaker than the following ones. This pattern has so far not been found in other species of *Eupholidoptera* in Crete. Recordings from other Greek Islands (Rhodes and Naxos) and published by Çiplak et al. (2009) show a comparable syllable duration (35–45 ms) and repetition rate (2–3/s). For details of sound recordings of *Eupholidopterasmyrnensis* from Crete see Suppl. material 3.

##### Differential diagnosis.

Males differ from congenerics in the stout, inward curved cerci (Figs 111, 126) with a strong basal side-tooth, in the subgenital plate (Figs 140, 154) slightly narrowing, tips with two small upward pointing teeth, the inner margin of the excision with a protuberance, in the long, apically inserted, backward pointing styli (Fig. 168), in the anal tergite (Figs 69, 83, 97) medially bend downward forming two strong, widely separated, downward pointing spines and in the fused wing-like expanded basal part of the apical arms of the titillator (Figs 182, 198), apically divided into two long parallel or diverging spines. Females differ from congenerics in the strongly convex subgenital plate (Figs 39, 53) with narrow, acute apical lobes, hind margin with wide excision as deep as a quarter or third of the length. In colouration *E.smyrnensis* differs from its Cretan congenerics in the black dots on the frons of the head merged into a mosaic pattern (Fig. 11), the unicolourous pale pronotal disc and a tiny central black dot along the hind margin of the abdominal tergites (Fig. 8). For more details differentiating *E.smyrnensis* from other Cretan *Eupholidoptera* see Table 5.

##### Distribution.

*Eupholidopterasmyrnensis* is one of the most widespread species of the genus. Its range covers western Anatolia and the southern Balkan (southern Bulgaria, southeastern Republic of North Macedonia, and north-western Greece) (Çiplak et al. 2009). Beside mainland Greece it has also been reported from a number of Aegean islands to the north (Thasos, Limnos), east (Samos, Nysiros, and Rhodes) as well as Evvoia (Monnerat et al. 1999: 65) and more to south from the Cyclades islands of Tzia (Willemse and Willemse 2008) and Naxos (unpublished data 2019). Much to our surprise, a single large population was discovered in 2017 in a small neglected patch of agricultural land full of blackberries (*Rubus*) amidst olive orchards southeast of Doxaro and west of the hamlet of Makrigiannis, central Crete in the lowlands south of the Taleon Mts. The species was collected again in 2019 in the southwestern most corner of Crete near the village of Lagkadas. Furthermore, pitfall catches from 1996 and 1997 revealed the presence of the species near Kavallos at the edge of Limni Kourna (Fig. 254). Cretan *E.smyrnensis* are smaller than mainland *E.smyrnensis*, but overall colour pattern and shapes of cercus, anal tergite, subgenital plate, and titillator fit with *E.smyrnensis* from other parts of Greece. For a complete list of localities, specimens, and repositories see Suppl. material 1.

##### Habitat.

Although on the Greek mainland *E.smyrnensis* is found up to 1200 m, in Crete, it has only been found between 25 m and 340 m. Unlike most other *Eupholidoptera* species in Crete, *E.smyrnensis* is not found in spiny shrubs on the ground but lives in taller spiny bushes like blackberries (*Rubus*).

##### Phenology.

Based on hand and pitfall catches *E.smyrnensis* becomes adult in June and near Limni Kourna adults have still been trapped after 20 August, probably being active well into September and possibly October.

### Phylogenetic analysis

The aligned concatenated dataset, which consisted of 1684 bp including 375 variable and 292 parsimony informative sites, involved ten ingroup and two outgroup taxa (respectively 36 ingroup and two outgroup haplotypes). The NADH2 fragment consisted of 855 bp including 353 variable and 279 parsimony informative sites, and the ITS had 829 bp with gaps, with 22 variable and 13 parsimony-informative sites. No numt signs were detected in alignments of protein-coding sequences, and the saturation tests did not show signs of significant saturation. Best substitution models for the partitioned dataset were as follows: NADH2, positions 1 and 2 – gamma, position 3 – gamma+ invariable sites; ITS – proportion of invariable sites.

**Figure 256.**
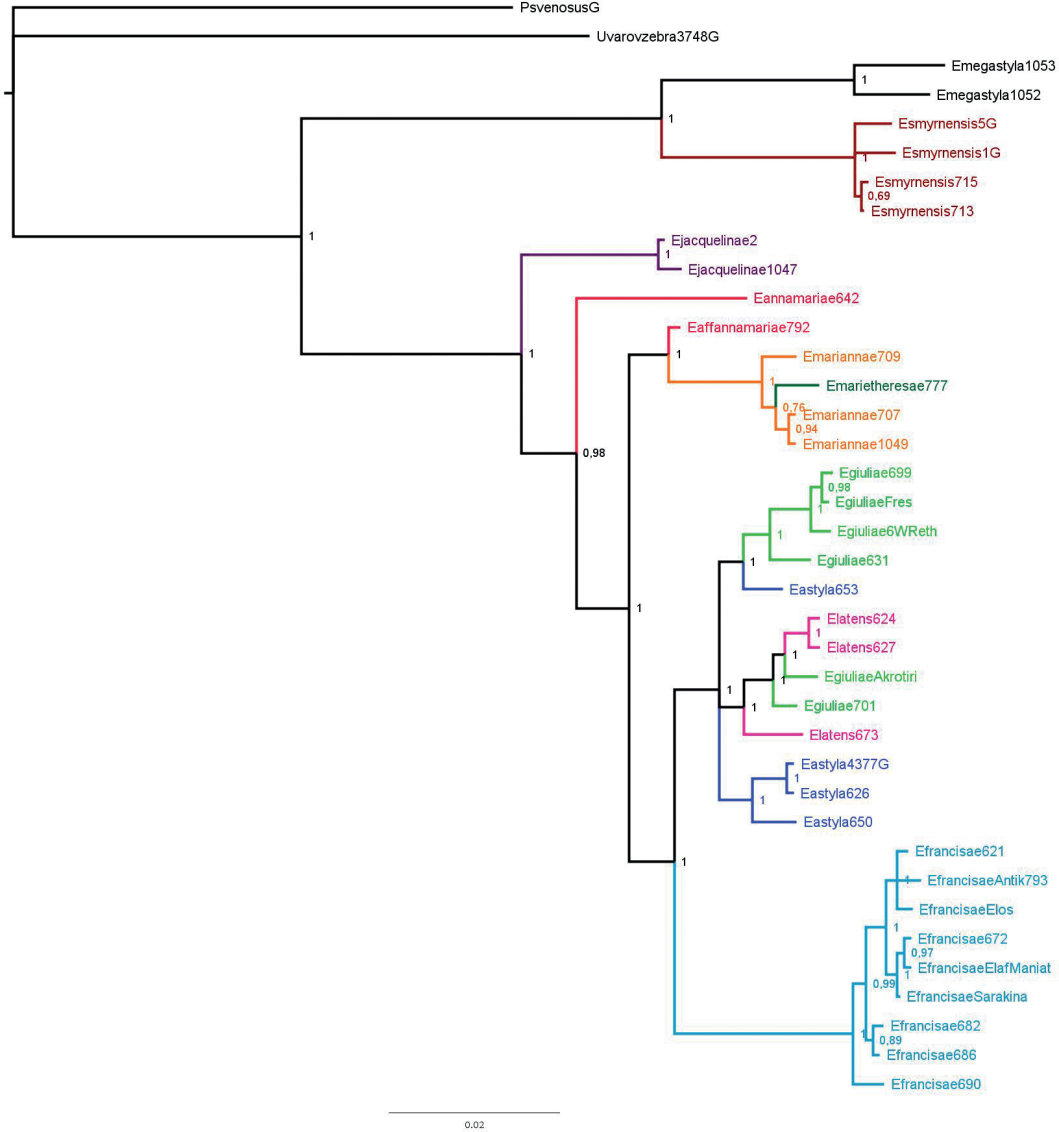
Bayesian inference phylogenetic tree of *Eupholidoptera* of Crete, Andikithira and Gavdos, including two outgroups and congenerics from the Greek and Turkish mainlands, based on a 1684bp concatenated alignment of the NADH2 + ITS fragments. Node values at branches show node support with BI posterior probabilities. Different colours correspond to the different morphological species. See Suppl. material 4 for details of the specimens and Suppl. material 5 for details of NADH2 and IT IS sequences.

Our phylogenetic analysis provided well resolved phylogeny of the studied taxa, showing strong support for all nodes (Fig. 256).

The taxa *E.latens*, *E.giuliae*, *E.francisae*, and *E.astyla* form a monophyletic lineage that splits into two major clades. The first clade contains all specimens occurring in western and southwestern Chania and Andikithira, showing very low genetic distances to each other (see also Fig. 258: yellow dots). The second clade is formed by three sub-clades: (1) most of *E.giuliae* samples (Fig. 258: dark blue dots), (2) *E.latens* s.str. (Fig. 258: purple dots) + some *E.giuliae* samples (Fig. 258: light blue dots), and (3) the *E.astyla* samples.

Some Cretan species appear to be paraphyletic: *E.marietheresae* is nested within *E.mariannae* despite their large morphological differences; an *E.annamariae* specimen found at the boundary of the *E.annamariae* and *E.mariannae* distributions appears to be more closely related to *E.mariannae* than to *E.annamariae*, despite its distinct morphology; and an *E.astyla* specimen is more closely related to *E.giuliae* than to other *E.astyla’s*.

**Figure 257.**
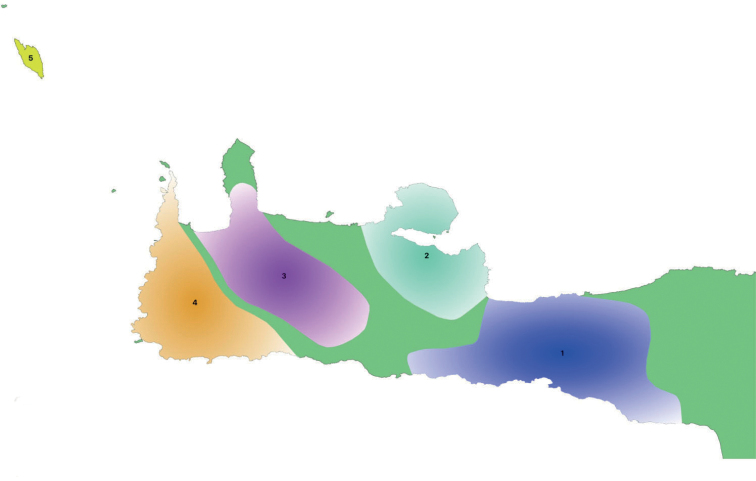
The *Eupholidopteragiuliae*-*latens*-*francisae* complex: geographic distribution of five morphological entities in the *Eupholidopteragiuliae*-*latens*-*francisae* complex: (1) eastern Chania + western Rethimno (*E.giuliae*), (2) northeastern Chania including Akrotiri peninsula (atypical *E.giuliae*), (3) northern and central Chania (*E.latens*), (4) western and southwestern Chania (*E.francisae*), (5) Andikithira (*E.francisae*).

**Figure 258.**
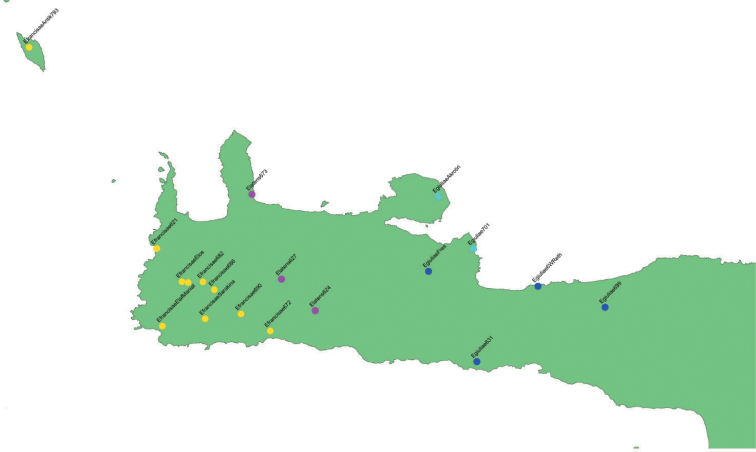
Distribution results genetic analysis: geographic pattern of analysis of genetic data of *Eupholidoptera* populations in western Crete. yellow dots: *E.francisae*, purple dots: *E.latens*, blue dots: *E.giuliae*, light blue dots: atypical *E.giuliae*.

The position of *E.marietheresae* inside the *E.mariannae* clade is an interesting observation, given their distinct morphological differences. Additional study is required, including the incorporation of *E.forcipata* sequences in the phylogenetic tree (the species morphologically closest to *E.marietheresae*), of more *E.marietheresae* specimens, and additional molecular (mostly nuclear) markers, in order to decipher the relationship between these species. Here it should be mentioned that the molecular results from Çiplak et al. (2021, 2022) do not support all hypotheses. From the few species studied there they suggest a relationship ((*latens*, *forcipata*)+(*giuliae*, *astyla*)), with a split between both groups ~ 2.5 Mya (Çiplak et al. 2022).

*Eupholidopterajacquelinae*, the endemic of the island of Gavdos, is the basalmost taxon of the Cretan clade. The only non-endemic *Eupholidoptera* on Crete, *E.smyrnensis*, is nested firmly within conspecifics from Anatolia. It should be noted that the nuclear fragment (ITS) showed very low interspecific variability. As a result, the topography of the concatenated tree is mostly influenced by the mitochondrial gene NADH2.

## ﻿Discussion

Together with western and southern Anatolia, Crete is a biodiversity hotspot for *Eupholidoptera* (Çiplak et al. 2009, 2010). This publication adds to the systematics, taxonomy, and faunistics of the genus in Crete and adjacent islands. For all species many new specimens, sound recordings, and distribution data have become available. This made it possible to describe all known species in detail, among which are two new species. The distribution patterns resulting from the new data confirmed disjunct distribution areas between some species but also provided evidence for the sympatric occurrence of others, and differences in altitudinal preferences became more evident. New data allowed for a better assessment of both infraspecific and intraspecific variation. This in turn led to questions about the species concept and potential evolutionary driving forces behind the present variation. These and other aspects of *Eupholidoptera* from Crete and adjoining islands are discussed in more detail below.

### ﻿The *E.latens–E.giuliae–E.francisae* complex

The current study revealed that based on morphological traits *E.giuliae*, *E.latens* and *E.francisae* may share a complex phylogeographical relationship.

Based on the shape of the anal tergite, subgenital plate, stylus and titillator of the male, five subgroups can be distinguished in western Crete and Andikithira (Table 8, Fig. 257). The first subgroup is found along coastal areas in the municipality of Sfakia in the southeastern part of Chania extending east into southern Rethimni and reaching northwards across the island to the region around the town of Rethimni (Fig. 257: area 1). Males are characterised by a wide central excision in the hind margin of the anal tergite bordered by short teeth, a wide subgenital plate with short inward pointing styli and compact apical arms of the titillator ending in long hooks. This group matches the description of *E.giuliae*. Populations in the municipalities of Chania and Apokoronas in the North (Drapanos, Garipa, Skloka) (Fig. 257: area 2) form a second subgroup. Males differ from the first group in the narrow excision in the hind margin of the anal tergite bordered by long teeth, the slimmer subgenital plate with long downward pointing styli and slender apical arms of the titillator. From the five subgroups, females in this region are atypical for *E.giuliae*, when compared to the first subgroup, in having slender subgenital plates which are distinctly longer than wide. Morphological characters in the third subgroup match the description presented for *E.latens*. It occurs at higher elevations on Mt. Lefka and in mid-level elevations and lowlands in the municipality of Platanias (Kolympari, Rodhopos, Prases, and Kato Kefalia) in central and northern Chania (Fig. 257: area 3). It is characterised by the wide central excision in the hind margin of the anal tergite bordered by short teeth, a slender subgenital plate with short to long downward pointing styli and compact apical arms of the titillator with short hooks. Populations found in the western and southwestern part of the Chania region, southwest from the line Gramvousa peninsula on the northwest coast to Livadas near the south coast (Fig. 257: area 4), form the fourth subgroup. They show some similarities with *E.latens* but differ in the very slender subgenital plate with apical lobes which in little more than half of the specimens carry a tiny spine at one or both tips. The populations in the fourth region very closely resemble *Eupholidoptera* populations found on Andikithira (Fig. 257: area 5) which possess an extremely slender subgenital plate with the tip of the apical lobes always provided with a spine, forming the fifth subgroup.

**Table 8.** Morphological differences across the *E.latens*-*E.giuliae*-*E.francisae* complex (see Fig. 257).

Subgroups	Anal tergite excision-teeth	Subgenital plate	Styli	Titillator
1. Eastern Chania + western Rethimni	wide-short	compact	short-inward	compact – long hooks
2. Northeastern Chania incl. Akrotiri	narrow-long	intermediate	long-downward	slender – long hooks
3. Northern and central Chania	wide-short	slender	long-downward	compact – short hooks
4. Western and southwestern Chania	narrow-short	very slender	short to long, downward	compact – short hooks
5. Andikithira	narrow-short	extremely slender	short, downward	compact – short hooks

Following the above pattern, populations in the western and southwestern corner of Crete (subgroup 4) represent a genetically well-outlined lineage, sharing the nuclear ITS fragment and its mitochondrial genome with the population on Andikithira (subgroup 5) (genetic distances of the nuclear internal transcribed spacers show very low variation among *Eupholidoptera*). The two islands have been isolated since the beginning of the Pleistocene (2.6 Mya), with only a narrow length of sea separating them during the Last Glacial Maximum (Lykousis 2009; Simaiakis et al. 2017; Fassoulas 2018; Bailey et al. 2022). This, combined with the small but stable morphological differences between the two isolated lineages and the deep marine strait between Crete and Andikithira suggest that the *Eupholidoptera* populations of Crete and Andikithira, have been isolated for a considerable length of time. The populations on Andikithira are morphologically very uniform with very little variation whereas populations from western and southwestern Chania show considerable morphological variation even within a single population. The reason for this could be the isolation of the Andikithira population, which did not permit genetic flow between it and other *Eupholidoptera* populations. This would lead to low genetic variability within the island of Andikithira, and, therefore, uniform morphological traits. A founder effect could have also taken place on the island with similar results. On the contrary, the populations from western and southwestern Chania were less isolated from each other and maintained a high genetic variability, that lead to the morphological diversity observed today. Interspecific gene exchange with populations of neighbouring taxa such as *E.giuliae* could have also taken place.

Based on the molecular results and the low morphological differences between populations from Andikithira and western and southwestern Chania (subgroups 4 and 5 in Table 8, Fig. 257), the decision was made to assign the populations from both areas, although closely related to *E.latens*, to *E.francisae* sp. nov.

The second clade may be regarded as a monophyletic mitochondrially-defined species complex of three subclades – the typical *E.giuliae* (morph-group 1), a clade formed by morph-groups 2 and 3, and a clade formed by *E.astyla* samples. The very low genetic mitochondrial distances between morphologically well outlined taxa (i.e., *E.astyla* and the rest) may point to former population crises (bottlenecks), where mitochondrial genome was shared between two or more taxa. At the same time, genetic drift caused by genetic bottlenecks may have contributed to unique characteristics (for instance, the peculiar shape of the titillators in *E.astyla*). On the other hand, the existence of specimens with symmetrical titillators and the intermixture of a tentatively identified female of *E.astyla* within the *E.giuliae* s.str. subclade (Fig. 256), calls for possible hybridisation in zones of syntopic occurrences.

An alternative scenario of the systematics of this western species complex may be proposed. Since the clade of morph-groups 2 and 3 show intermediate and variable morphology between specimens of *E.francisae* (morph-group 4) and *E.giuliae* (morph-group 1), and they intermix at the phylogenetic tree, those populations may represent a hybridogenic lineage of two formerly partially speciated taxa or may still be in the ‘grey zone’ of their evolutionary differentiation where they can either become distinct taxa or merge back into a common genetic pool in future.

The above scenario may not be unique and could be expected in other sibling species of Cretan *Eupholidoptera* that express intermediate or variable morphological characters (e.g., *E.mariannae* and *E.annamariae*). As results based on morphology and genetics are not unequivocal and boundaries do not match, as yet no taxonomic decision has been taken for morph-groups 1, 2, and 3. For the time being, both the eastern groups (morph-groups 1 and 2) have been assigned to *E.giuliae*, whereas the central group (morph-group 3) matches *E.latens*.

### ﻿Titillators

The diversity of male insect genitalia is well known (Simmons 2014). They are even considered as “the most variable and divergent of all morphological structures”. In *Eupholidoptera* mainly four different parts are involved: cercus, titillator, subgenital plate and last abdominal tergite. In the Cretan species all these structures vary between the species, but the most variable ones are the titillators. While the other three structures seem to be connected with some kind of fit between male and female genitalic organs, the function of the titillators remained unclear for a long time because they are typically concealed within the abdomen during copulation. Just recently, Wulff et al. (2017) demonstrated by in vivo X-ray cineradiography in the related *Roeselianaroeselii* that the titillator is moved rhythmically during mating. The authors assume that it is “initially used for stimulation” and perhaps later also involved in spermatophore transfer. In *Eupholidoptera* rhythmical movements of the titillator have also been observed (Dagmar von Helversen, pers. comm., probably before 2000), possibly visible externally due to its large size. The rapid evolution in shape may occur under sexual selection (see Simmons 2014; Wulff et al. 2017) and *Eupholidoptera* may be an interesting study object where species-specific differences of varying magnitude in size, curvature and symmetry can be found.

### ﻿Asymmetry

Although in some orders of insects, asymmetry of genitalia is the ground plan, in most insects, including Orthoptera, it is very rare (Huber et al. 2007). In Cretan *Eupholidoptera* asymmetric titillators are found in *E.annamariae*, *E.feri* and by far the most evident example is *E.astyla*. In male *E.astyla* titillators are antisymmetric asymmetric, a quite unique and rare feature (Schilthuizen 2013). Of 13 males studied, the tip of the titillator is pointing to the right in 8, and to the left in 5 males. This category of asymmetry has been coined “pure antisymmetry”. Sexual selection has played a crucial role in the evolution of insect genital asymmetry (Huber et al. 2007; Huber 2020) via the route of mating positions. Available data strongly corroborate a correlation between morphological asymmetry and one-sided mating positions, whereas symmetric genitalia allow for random-side positions. The few images available on *Eupholidoptera* mating indicate that their mating position is end-to-end with the heads of the male and female pointing in opposite directions, that of the male pointing to the female ovipositor. It would be interesting to know whether in *E.astyla* mating positions differ from those in *Eupholidoptera* species with symmetric genitalia. In this context it is striking that the titillator in two male *E.astyla* from the Asterousia Mts (Fig. 192) was symmetrical. None of the 30 other specimens trapped at the same site showed this anomaly.

### ﻿Bioacoustics

**Figure 259.**
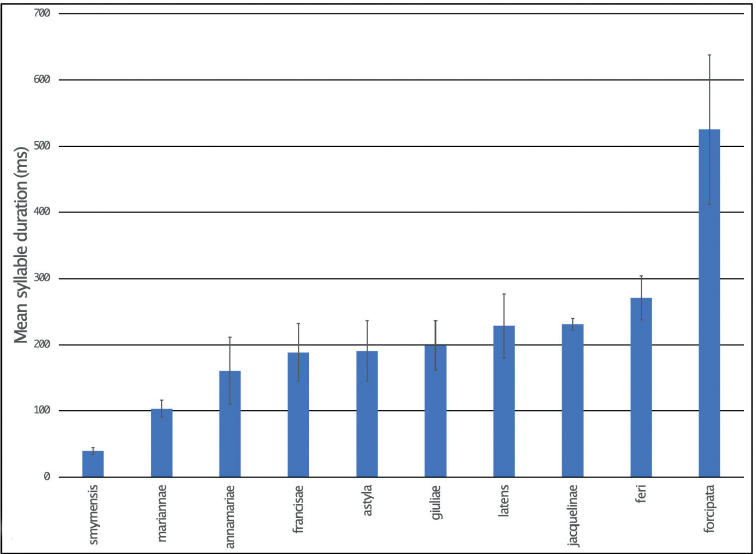
Mean syllable duration in recorded species from Crete (incl. Gavdos and Andikithira). See Suppl. material 3 for measurements per recorded specimen.

The song of *Eupholidoptera* is quite uniform throughout the genus and is even considered a not very reliable character for discerning species (Çiplak et al. 2009). It consists of isolated syllables produced in long series with the opening hemisyllable much shorter and weaker than the closing hemisyllable. In some cases, the opening hemisyllable is partly or not visible in the recordings, which may be a character of the song of a specimen. It may, on the other hand, also reflect the quality of the sound recording. In the known Cretan species, syllable duration is between 40 ms and 600 ms (Fig. 259). During active singing the syllable repetition rate may be as fast as 2/s but is often much slower. In the best available recordings, single tooth impacts may be visible in the closing hemisyllable, each corresponding with one tooth on the stridulatory file.

In quite some recordings it is not easy to clearly discern the opening hemisyllable, leaving discussion whether it is just weak and may be not well recorded or it is absent, with no sound produced during this wing movement. As a main character of the species’ song may be the duration of the syllable, the absence of the opening hemisyllable in some recordings or specimens accounts for inaccurate measurements of this song character. The same holds for the effect of temperature on the duration of syllables, which may last twice as long with temperatures differing only as little as 2–5 °C. This adds to the fact that in many cases not so many specimens and not so much time (actively) singing has been available to us to compare the song of the species thoroughly. Bioacoustic measurements presented in Fig. 259 include all measurements regardless of their quality or ambient temperature. Fig. 259 shows that *E.smyrnensis* and *E.forcipata* produce a song with a syllable duration clearly different from all other Cretan species analysed. When considering the relatively small differences in syllable duration in the other species these could be species-specific but could also be explained by differences in temperature during the recording or the low number of recordings. No statistical analysis has been performed and no attempt has been undertaken to correct for differences in temperature. Within the species that show morphological variation throughout their range (*E.giuliae*, *E.francisae*, and *E.latens*) no variation in song characters could be found. Between the morphologically related *E.latens* and *E.francisae* no systematic difference in song characters could be demonstrated. Maybe a larger number of standardised sound recordings (especially standardised for temperature) may yield in more systematic differences between taxa.

Most Cretan species of *Eupholidoptera* are allopatric and a female will only hear the calling song of conspecific males. The syllable duration between most Cretan species is largely overlapping and not distinctive (but long for *Eupholidoptera* in general; Çiplak et al. 2009) and differences in morphology fit into the conclusion by Heller (2006) that regarding differentiation of allopatric populations, changes in calling songs seem to appear more slowly than changes in morphology. In some locations Cretan species of *Eupholidoptera* occur sympatrically (Table 9). Interestingly, nine out of ten sympatric occurrences detected included a species with an unusual song pattern, either *E.smyrnensis* with shorter syllables than all others or *E.forcipata* (respectively, the similar *E.marietheresae* sp. nov. with probably similar songs) with distinctly longer syllables than all others (see Fig. 259). Besides *E.latens*, the 10^th^ example included *E.cretica* for which the calling song is not yet recorded. Obvious song differences between sympatric *Eupholidoptera* species were also listed in Çiplak et al. (2009). If male calling songs in two sympatric species differ this of course helps females to locate conspecific males. Concerning the evolution of these differences, the Cretan recordings of the wide-spread *E.smyrnensis* do not differ from recordings made elsewhere in its range so the species has obviously not changed its song when coming to Crete. In contrast, the extraordinarily long syllables of *E.forcipata* may be an important factor allowing the co-existence with other Cretan *Eupholidoptera*.

**Table 9.** Examples of syntopic occurrences of *Eupholidoptera* species in Crete.

Trap no.	Coll. date	Location	GPS Coordinates	Alt. (m)	Species 1	Species 2
FC111	30/10/1996	Limni Kourna	35.3269, 24.2790	25	*giuliae*	*smyrnensis*
FC495	10/07/1997	Limni Kourna	35.3269, 24.2790	25	*giuliae*	*smyrnensis*
FC70	20/08/1996	Limni Kourna	35.3269, 24.2790	25	*giuliae*	*smyrnensis*
FC1536	05/08/2000	Dikti Mt.	35.1107, 25.4779	1715	*astyla*	*marietheresae* sp. nov.
FC1606	02/10/2000	Dikti Mt.	35.1107, 25.4779	1715	*astyla*	*marietheresae* sp. nov.
FC1655	09/01/2001	Dikti Mt.	35.1107, 25.4779	1715	*astyla*	*marietheresae* sp. nov.
FC1602	15/09/2000	Idi Mt.	35.1973, 24.7920	1910	*forcipata*	*gemellata*
FC1651	30/10/2000	Idi Mt.	35.1973, 24.7920	1910	*forcipata*	*gemellata*
FC1916	12/06/2001	Idi Mt.	35.1973, 24.7920	1910	*forcipata*	*gemellata*
FC17807	19/10/2018	Lefka Mt.	35.3524, 23.9050	1200	*cretica*	*latens*

### ﻿Geographical distribution

Findings presented here, collected over the past 30 years, show that the known distribution range for five *Eupholidoptera* species in Crete (*E.annamariae*, *E.astyla*, *E.giuliae*, *E.latens*, and *E.mariannae*) is larger than previously known (Fig. 254). *Eupholidoptera* has now been found in most areas of Crete, occurring nearly everywhere on the island. Up to now *Eupholidoptera* has been overlooked at low altitudes due to its early appearance. For instance, recent findings of *E.latens* indicate that at lower altitudes animals are already adult at the beginning of May, while in the mountains adults only appear at the end of July or early August.

### ﻿Altitudinal preferences

Of the 14 species of *Eupholidoptera* treated in this paper, six species have only been found at higher altitudes (above 1000 m): *E.pallipes*, *E.cretica*, *E.gemellata*, *E.forcipata*, *E.marietheresae* sp. nov., and *E.feri*, five species are restricted to lower altitudes (below ca. 500 m): *E.francisae* sp. nov., *E.giuliae*, *E.jacquelinae*, *E.annamariae*, and *E.smyrnensis*, whereas data presented here indicate that *E.astyla*, *E.latens*, and *E.mariannae* are found across a wide altitudinal range from sea level to higher altitudes.

### ﻿Traps

Pitfall traps are widely used to monitor ground dwelling invertebrates. For Orthoptera trapping results provide a contradictory picture (Schirmel et al. 2010). Tomar et al. (2017) compared bioacoustics and pitfall traps to monitor the Ensifera in suburban Delhi, India. In this study bioacoustics resulted in more species, but fewer specimens compared to pitfall traps. Differences were explained in the way bioacoustics (active) and pitfall traps (passive) operated and in possible microhabitat preferences, which affect pitfall trap results. The fact that quite a number of *Eupholidoptera* species (and other bush-cricket and cricket genera for that matter) across Crete have been trapped in pitfall traps (often in large numbers), is a strong indication that during the night their natural behaviour includes walking over the ground. This applies to species found in phrygana, inhabiting low prickly bushes. Some species like *E.cretica* and *E.mariannae* were caught using fermenting traps that hung from trees and tall bushes indicating these species prefer to live in higher shrubs and trees in which they probably hide during the day. Summarising, the results presented here for trap catches provide a strong indication that traps may provide a valuable addition to monitoring techniques used for Orthoptera in general and *Eupholidoptera* in particular.

### ﻿Sympatry

Little information was available on the sympatric occurrences of different *Eupholidoptera* species. The syntopic occurrence of *latens/pallipes* on Mt. Lefka and *gemellata/forcipata* on Mt. Idi was conjected but not proven. Çiplak et al. (2009) describe the syntopic (and synchronic singing, unpublished) occurrence of *astyla* and *forcipata* on Mt. Idi. Results from pitfall catches provide further proof of syntopic occurrences. Of a total of some 150 pitfall events (location + collecting date), ten events at four separate locations resulted in two *Eupholidoptera* species ending up in the same trap (Table 9).

However, one must bear in mind that the period between activating the traps and collecting the accumulated specimens was at least two months. Therefore, it cannot be ruled out that despite the sympatry, species do not (completely) overlap in their phenology or daily activity patterns. Interestingly, in nine of the ten trapping events a species with unusual song pattern is involved, either *smyrnensis* with shorter syllables than all others or *forcipata* (respectively the similar *marietheresae* sp. nov. with probably similar song) with distinctly longer syllables than all others (see Fig. 259). It would probably be interesting to study their coexistence in more detail by an intense field survey with observations of interspecific behaviour.

### ﻿Conservation status

Recently, a Red List for all European Orthoptera species has been compiled, including the *Eupholidoptera* species of Crete (Hochkirch et al. 2016). Except for *E.smyrnensis*, all Cretan species are classified as threatened (Critically Endangered, Endangered, or Vulnerable) (Table 10). Based on the information presented in this publication the status for widespread species like *E.astyla*, *E.giuliae*, *E.latens*, and *E.annamariae* is likely to change to Least Concern. For other species with a (very) restricted area of occurrence, the Red List Status is expected not to change, underpinning the necessity to protect the habitats of these species. The two new species still have to be assessed. Most likely *E.francisae* will be evaluated as Least Concern based on its geographic range and *E.marietheresae* as Vulnerable based on its very restricted geographic range.

**Table 10.** Current IUCN Red List Status for *Eupholidoptera* species of Crete and adjacent islands.

Species	Common name	IUCN RLA status*
*E.annamariae*	Annamaria’s Marbled Bush-cricket	VU
*E.astyla*	Mount Ida Marbled Bush-cricket	EN
*E.cretica*	Cretan Marbled Bush-cricket	VU
*E.feri*	Fer’s Marbled Bush-cricket	CR
*E.forcipata*	Idi Marbled Bush-cricket	VU
*E.francisae* sp. nov.	Francis’s Marbled Bush-cricket	Not Evaluated
*E.gemellata*	Skaronero Marbled Bush-cricket	VU
*E.giuliae*	Giulia’s Marbled Bush-cricket	VU
*E.jacquelinae*	Jacqueline’s Marbled Bush-cricket	VU
*E.latens*	Hidden Marbled Bush-cricket	VU
*E.mariannae*	Marianne’s Marbled Bush-cricket	VU
*E.marietheresae* sp. nov.	Marietherese’s Marbled Bush-cricket	Not Evaluated
*E.pallipes*	Pale-legged Marbled Bush-cricket	VU
*E.smyrnensis*	Smyrna Marbled Bush-cricket	LC

^*^: CR – Critically Endangered, EN – Endangered, VU – Vulnerable, LC – Least Concern.

### ﻿Citizen scientists and observations

To further close the gaps in our knowledge, observations made during excursions by tourists in Crete can be very useful especially if these are uploaded to online platforms like iNaturalist and Observation.org. This paper includes nine observations on *Eupholidoptera* from Crete (Suppl. material 1), which based on images were assigned to *E.giuliae* (7), *E.pallipes* (1), and *E.jacquelinae* (1). The importance of social media and citizen science cannot be emphasised enough. They may help to solve systematic puzzles like in the case of the Australian pygmy grasshoppers (Skejo et al. 2020). However, one must bear in mind that in case of *Eupholidoptera*, species are nocturnal and less likely to be observed during the day, and that caution is advised when identifying species from photos, since important diagnostic characters might not be visible.

### ﻿Phylogeography

Fourteen species of *Eupholidoptera* in the Cretan area is a remarkable gathering of taxa, albeit not unique across various invertebrate orders. Other examples include for instance *Dendarus* (Coleoptera: Tenebrionidae) with 13 species (Trichas 2008), *Mastus* (Gastropoda: Enidae) with 16 species (Parmakelis et al. 2005) and *Albinaria* (Gastropoda: Clausiliidae) with 31 species (Welter-Schultes 2010). Although the taxonomy in *Albinaria* (Dimopoulou et al. 2017) and *Dendarus* (Trichas et al. 2020) is still a matter of debate, these large numbers of unique endemic taxa are indicative of the isolation and “speciation dynamics” of Crete.

This high concentration of endemic species of *Eupholidoptera*, and especially the presence of some primitive representatives (*E.gemellata*, *E.pallipes*), has led previous researchers to propose the southeastern Aegean plate, possibly including Crete, as the origin place of the last common ancestor of *Eupholidoptera* (Çiplak et al. 2009, 2010). Phylogenetic analyses by Çiplak et al. (2021, 2022) based on molecular markers confirmed this hypothesis, suggesting that *Eupholidoptera* split from its closest relative (*Parapholidoptera*) 12 Mya, during the Serravallian. The last common ancestor of *Eupholidoptera* was dated to the Tortonian during the regression of the Mid-Aegean Trench (12–9 Mya), in an area around south Anatolia and Crete. This ancestor probably possessed a larger range that was split due to the Aegean tectonic movements, and then later dispersed to the rest of Greece and the Balkans (Çiplak et al. 2022).

Of the fourteen species of *Eupholidoptera* only one, *E.smyrnensis*, has a large distribution area. The finding of *Eupholidopterasmyrnensis* on Crete adds new information to the phylogeography of the *Eupholidopterachabrieri* group of species as presented by Çiplak et al. (2010). Studies with genetic markers should elucidate whether *E.smyrnensis* reinvaded Crete using terrestrial corridors that existed during the Messinian Salinity Crisis (5.96–5.33 Mya) or whether its presence is linked to recent (human?) introduction. According to Çiplak et al. (2022) the branch with *E.smyrnensis* separated from the other studied Cretan *Eupholidoptera* species ~ 8 Mya.

Only one *Eupholidoptera* species is not found on Crete itself but restricted to surrounding islands. Gavdos and the small islet of Gavdopoula are home to *E.jacquelinae*. The islands are surrounded by a fairly extensive shelf that was exposed above sea level during Mio-Pliocene but were never connected to Crete during the Quaternary (2.58 Mya up to now) (Kasapidis et al. 2005; Sakellariou and Galanidou 2015). Estimates of the isolation of Gavdos and Gavdopoula from Crete range from 2.4 to 4.8 Mya, depending on author (Anastasakis 1987; Van Hinsbergen and Meulenkamp 2006; Broggi 2015; Poulakakis et al. 2015; Fassoulas 2018). Despite their long isolation, lineages of various terrestrial groups found on Gavdos and Gavdopoula are heavily influenced by the Cretan terrestrial counterparts. Examples are the land-snail genus *Mastus* (Welter-Schultes 2000; Parmakelis et al. 2005), beetle genera like *Dendarus* (Trichas et al. 2020) and *Drilus* (Kundrata et al. 2015), as well as the scorpion *Mesobuthus* (Parmakelis et al. 2006), where the populations on Gavdos and Gavdopoula are Pleistocene descendants from Cretan terrestrial taxa. Given the amount of diversification of *E.jacquelinae*, only an old vicariant event (somewhere between 2.4 and 4.8 Mya) or a quite old dispersal scenario (towards the upper half of Pleistocene) could explain the presence of the ancestors of *E.jacquelinae* on Gavdos and Gavdopoula. But one must have in mind that both explanations seek for closest relatives on the Cretan land, while so far, they are found only more remotely (Tilmans 2002). A more complete phylogeny of all Cretan *Eupholidoptera* taxa with several genetic markers, better time estimations and a more precise evaluation of the relationships between them, could provide more definitive insights on the *E.jacquelinae* origin and its relationship with the other Cretan taxa.

Another interesting phylogeographical matter is that of *Eupholidopterafrancisae* occurring on the island of Andikithira (32 km NW of western Crete) and in the extreme western/southwestern part of Crete. The faunal affinities between Andikithira and W. Crete and their biogeographical implications in the area, have a long story of theories and debates, starting from “Boettger’s line” (between Kithira and Andikithira) and the Peloponnese-Kithira-Andikithira-Crete relations (Boettger 1894; Gittenberger 1990; Gittenberger and Goodfriend 1993). These authors, based on the distributions of terrestrial gastropods, concluded that Andikithira was not connected to Kithira or to Crete during the entire Pleistocene which at the time these papers were published was considered to have started ~ 1,8 Mya ago. Sfenthourakis (1993), in an analysis of the isopod fauna of Andikithira (plus the surrounding satellite islets of Pori and Poreti), postulated older connections between Andikithira and Crete, but also disconnections and reconnections with both Kithira and Crete during the Pliocene/Pleistocene. All of the above authors, more or less agree that the geological separation between Kithira and Andikithira is a very old one, that between Andikithira and Crete younger, and the one between Kithira and the Peloponnese Peninsula the youngest (Gittenberger and Goodfriend 1993). Moreover, a recent discovery of a unique lizard species on the small Andikithira satellite islets of Pori and Poreti (*Podarcislevendis*Lymberakis et al., 2008), is also indicative of the amount of time isolation of the small Andikithira archipelago, although does not elucidate the Andikithira-Crete geological relationship. Similarly, *Dendarusantikythirensis* from Andikithira and Pori islet is very closely related to *D.graecus* from Crete, but again, not informative enough on the Andikithira-Crete relationship (Trichas et al. 2020). Both taxa were interpreted as long-distance dispersals from their Cycladic (central Aegean) ancestors to Andikithira and Crete respectively. From a geological/paleogeographic point of view it seems very probable that there were no solid land connections between Andikithira and Crete at least for the last 2 Mya, but many authors (e.g., Lykousis 2009) postulate very narrow sea strait between Andikithira and Crete, at the period of Late Pleistocene (480–350 Kya). Given the amount of geological isolation of Andikithira and the genetical characteristics of the two populations of *E.francisae* on Andikithira and W. Crete, we can only speculate on a dispersal event from western/southwestern Crete to Andikithira, with the Late Pleistocene period as a good candidate for that event.

One more important question is, what caused the diversification of the 11 endemic *Eupholidoptera* species restricted to Crete itself and what factors contributed to their current distribution patterns? Although phylogeographic studies over the past 30 years revealed two major geological events that contributed to the diversification in the Aegean – the formation of the mid-Aegean trench (MAT) at the end of the Miocene (12–9 Mya) that separated Crete from the west Aegean and Anatolia, and the final isolation of Crete from Peloponnisos after the Messinian Salinity Crisis (MSC, 5.33 Mya) – solid evidence of exact patterns of diversification via in situ speciation within individual islands in the Aegean, i.e. Crete, remains still scarce (Poulakakis et al. 2015).

Several studies on monophyletic lineages of invertebrates and vertebrates (Table 11) in Crete using mitochondrial and nuclear markers indicate the existence of two major east-west clades and possible subclades, depending on the age of the arrival of the taxon in question. Pliocene paleogeography contributed to these well separated east-west units in many animal groups that arrived (or left?) on that south Ägäis piece of land after the Zanclean Flood (Krijgsman et al. 1999; Blanc 2002; Lymberakis and Poulakakis 2010).

**Table 11.** Cretan invertebrate and vertebrate monophyletic lineages and time of divergence of East-West clades.

Taxon	Order	Number of subclades	Diverged approximately at	Reference
*Cyrtocarenumcunicularium*	Araneae	2	3.3 Mya	Kornilios et al. (2016)
*Poecilimoncretensis*	Orthoptera	2	0.6 – 1.2 Mya	Borissov et al. (2020)
*Thaumetopoeawilkinsoni*	Lepidoptera	2	< 1 Mya	Petsopoulos et al. (2018)
*Reticulitermes*	Blattodea	4	< 1 Mya	Fig. 2B in Velonà et al. (2010)
*Carabusbanoni*	Coleoptera	2	1.47 Mya	Vlachopoulos et al. (pers. Comm.)
*Podarciscretensis*	Squamata	2	5.42 – 4.36 Mya	Psonis et al. (2021)*

*: but see also Kyriazi et al. 2013; Spilani et al. 2019 for much smaller divergence time estimations for *Podarciscretensis* east-west clades, 2.9–0.48 Mya.

East-west divergences are also evident in Cretan *Eupholidoptera* lineages, but the question whether the species found in Crete and the adjacent islands are polyphyletic as concluded by Çiplak et al. (2010) or, apart from *E.smyrnensis*, are monophyletic still needs a more definite answer. The study of 2010 did not use any molecular data but only morphological traits and song data, while the most recent molecular study (Çiplak et al. 2022) covers only four Cretan taxa (*E.forcipata*, *E.giuliae*, an unidentified species which is most likely *E.latens*, and *E.astyla*, in addition to *E.smyrnensis*). Lastly, the phylogenetic tree presented here does not include some crucial taxa such as *E.gemellata*, *E.pallipes*, *E.cretica*, and *E.forcipata* which, according to Çiplak et al. (2010), are the most distinct morphologically and are placed in clades distant from the rest of the Cretan taxa. Additional samplings of these rare species will provide a clearer picture on the monophyly of the Eupholidoptera of Crete.

**Figure 260.**
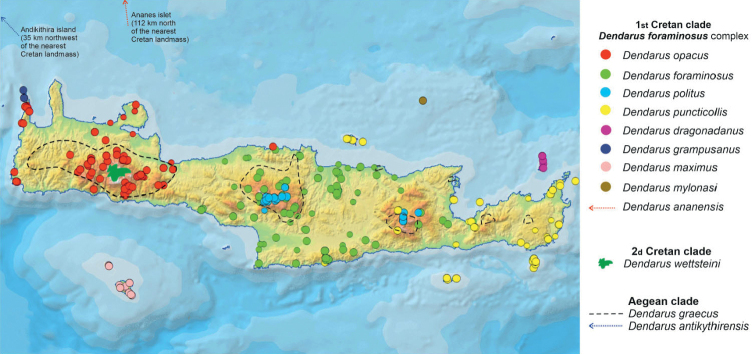
Distribution pattern of Cretan *Dendarus* taxa (Coleoptera), map visualised from data obtained from Trichas et al. (2020) and Trichas (2008).

An example of another polyphyletic genus under investigation on Crete is *Dendarus* (Coleoptera: Tenebrionidae) that consists of three distinct lineages on the island (two mitochondrial, one nuclear gene locus, and 61 morphological characters; Trichas et al. 2020) and includes 13 species. Distribution patterns in wingless *Dendarus* darkling beetles (Fig. 260) are quite similar in some extend to those found in *Eupholidoptera*. They share, for instance, high mountain taxa with similar distributions *as E.forcipata*, *E.gemellata*, and *E.pallipes* (*D.politus* and *D.wettsteini* are distributed on the same mountaintops, Fig. 260), as well as lowland species, with *E.astyla* most strikingly similar in distribution to *D.foraminosus*. *Eupholidopteragiuliae* occupies only west Cretan lowlands much like *D.opacus*. Also, *E.jacquelinae* is distributed on both Gavdos and Gavdopoula islets, exactly as *Dendarusmaximus* does, while the morphologically distinguishable number of taxa in the area is almost the same. Is there any ground to consider a similar number of different lineages, too? Given the new findings of *E.smyrnensis* populations on Crete, one can speculate even on six different groups at least on Crete mainland alone, but how many clades do we have? Are the west (*E.latens*+*E.giuliae*+*E.francisae*+*E.cretica*), central/central-east (*E.astyla*+*E.forcipata*+*E.marietheresae*), and east groups (*E.annamariae*+*E.mariannae*+*E.feri*) monophyletic like the *Dendarusforaminosus* complex? Are *E.pallipes*+*E.gemellata*, like *Dendaruswettsteini*, relictual equivalents and is *E.smyrnensis* a recent Aegean dispersal (much like *Dendarusgraecus*)? Or are we facing a totally different range of evolutionary events? Along with the interrelations between groups, it would be interesting to investigate further the depth and time of divergence between lowland and high-altitude taxa in each group, i.e., how deep is the distance between *E.astyla* and *E.forcipata* (and when did they diverge)? Are the timings of divergence of Pleistocene age or even shallower? Answering all these questions raised by the unique diversity and complicated distribution patterns of *Eupholidoptera* in Crete requires a thorough phylogenetic/phylogeographic study.

## Supplementary Material

XML Treatment for Eupholidoptera annamariae

XML Treatment for Eupholidoptera astyla

XML Treatment for Eupholidoptera cretica

XML Treatment for Eupholidoptera feri

XML Treatment for Eupholidoptera forcipata

XML Treatment for Eupholidoptera francisae

XML Treatment for Eupholidoptera gemellata

XML Treatment for Eupholidoptera giuliae

XML Treatment for Eupholidoptera jacquelinae

XML Treatment for Eupholidoptera latens

XML Treatment for Eupholidoptera mariannae

XML Treatment for Eupholidoptera marietheresae

XML Treatment for Eupholidoptera pallipes

XML Treatment for Eupholidoptera smyrnensis

## Supplementary materials

Supplementary material 1 List of localities, specimens, and repositories of *Eupholidoptera* from Crete, Gavdos, Gavdopoula, and Andikithira Data type occurrence data (excel document) Explanation note The table contains all published and unpublished localities, specimens collected and their repositories for all *Eupholidoptera* species from Crete and its satellite islands known to the authors. https://binary.pensoft.net/file/817964This dataset is made available under the Open Database License (http://opendatacommons.org/licenses/odbl/1.0/). The Open Database License (ODbL) is a license agreement intended to allow users to freely share, modify, and use this Dataset while maintaining this same freedom for others, provided that the original source and author(s) are credited.Luc Willemse, Jos Tilmans, Nefeli Kotitsa, Apostolos Trichas, Klaus-Gerhard Heller, Dragan Chobanov, Baudewijn Odé

Supplementary material 2 Examined specimens Data type specimen studied (excel document) Explanation note This table lists examined specimens with an indication of how they are stored (D: dry pinned; W: in alcohol), whether they have been measured (mm), photographed (image), sampled for DNA analysis (DNA), or audio recorded (audio). Abbreviations used: √: done; S: stacked images(s); F: field image from life specimen; H: habitus image from collection specimen; FC: trap. For more details of location and trap see Suppl. material 1. Specimens are arranged in alphabetical order of species and within species on region and location. https://binary.pensoft.net/file/817965This dataset is made available under the Open Database License (http://opendatacommons.org/licenses/odbl/1.0/). The Open Database License (ODbL) is a license agreement intended to allow users to freely share, modify, and use this Dataset while maintaining this same freedom for others, provided that the original source and author(s) are credited.Luc Willemse, Jos Tilmans, Nefeli Kotitsa, Apostolos Trichas, Klaus-Gerhard Heller, Dragan Chobanov, Baudewijn Odé

Supplementary material 3 Sound recordings and song data Data type bioacoustic recordings (excel document) Explanation note table with detailed information on sound recordings and song data. The tab sound recordings summarises details of sound recordings of Eupholidopteraspecies from Crete, Andikithira, and Gavdos. Recordings are in the collection of Jos Tilmans, Baudewijn Odé, and Klaus-Gerhard Heller. The tab song details provides information about the ambient temperature and mean syllable duration for individual specimen. https://binary.pensoft.net/file/817966This dataset is made available under the Open Database License (http://opendatacommons.org/licenses/odbl/1.0/). The Open Database License (ODbL) is a license agreement intended to allow users to freely share, modify, and use this Dataset while maintaining this same freedom for others, provided that the original source and author(s) are credited.Baudewijn Odé, Jos Tilmans, Klaus-Gerhard Heller

Supplementary material 4 Details of specimens used in phylogenetic analysis Data type table (excel document) Explanation note Details of specimens used for the Bayesian inference phylogenetic tree of 9 *Eupholidopteraspecies* from Crete, Andikithira, and Gavdos (see Figs 256, 258). https://binary.pensoft.net/file/817967This dataset is made available under the Open Database License (http://opendatacommons.org/licenses/odbl/1.0/). The Open Database License (ODbL) is a license agreement intended to allow users to freely share, modify, and use this Dataset while maintaining this same freedom for others, provided that the original source and author(s) are credited.Nefeli Kotitsa, Dragan Chobanov

Supplementary material 5 NADH2___ITIS_sequences Data type table (excel document) https://binary.pensoft.net/file/817968This dataset is made available under the Open Database License (http://opendatacommons.org/licenses/odbl/1.0/). The Open Database License (ODbL) is a license agreement intended to allow users to freely share, modify, and use this Dataset while maintaining this same freedom for others, provided that the original source and author(s) are credited.Nefeli Kotitsa, Dragan Chobanov
